# Experienced fatigue in people with rare disorders: a scoping review on characteristics of existing research

**DOI:** 10.1186/s13023-021-02169-6

**Published:** 2022-01-10

**Authors:** Trine Bathen, Heidi Johansen, Hilde Strømme, Gry Velvin

**Affiliations:** 1 TRS Resource Centre for Rare Disorders, Sunnaas Rehabilitation Hospital, 1450 Nesoddtangen, Oslo, Norway; 2 Library of Medicine and Science, University of Oslo, Oslo, Norway

**Keywords:** Experienced fatigue, Subjective fatigue, Perceived fatigue, Rare disease, Rare disorder, Rare developmental defect, Rare genetic disorder, Scoping review

## Abstract

**Background:**

Experienced fatigue is an under-recognized and under-researched feature in persons with many different rare diseases. A better overview of the characteristics of existing research on experienced fatigue in children and adults with rare diseases is needed. The purpose of this review was to map and describe characteristics of existing research on experienced fatigue in a selection of rare diseases in rare developmental defects or anomalies during embryogenesis and rare genetic diseases. Furthermore, to identify research gaps and point to research agendas.

**Methods:**

We applied a scoping review methodology, and performed a systematic search in March 2020 in bibliographic databases. References were sorted and evaluated for inclusion using EndNote and Rayyan. Data were extracted on the main research questions concerning characteristics of research on experienced fatigue (definition and focus on fatigue, study populations, research questions investigated and methods used).

**Results:**

This review included 215 articles on ten different rare developmental defects/anomalies during embryogenesis and 35 rare genetic diseases. Of the 215 articles, 82 had investigation of experienced fatigue as primary aim or outcome. Included were 9 secondary research articles (reviews) and 206 primary research articles. A minority of articles included children. There were large differences in the number of studies in different diseases. Only 29 of 215 articles gave a description of how they defined the concept of experienced fatigue. The most common research-question reported on was prevalence and/ -or associations to fatigue. The least common was diagnostics (development or validation of fatigue assessment methods for a specific patient group). A large variety of methods were used to investigate experienced fatigue, impeding comparisons both within and across diagnoses.

**Conclusion:**

This scoping review on the characteristics of fatigue research in rare diseases found a large variety of research on experienced fatigue. However, the minority of studies had investigation of experienced fatigue as a primary aim. There was large variation in how experienced fatigue was defined and also in how it was measured, both within and across diagnoses. More research on experienced fatigue is needed, both in children and adults with rare diseases. This review offers a basis for further research.

**Supplementary Information:**

The online version contains supplementary material available at 10.1186/s13023-021-02169-6.

## Introduction

Experienced fatigue is an important, under-recognized and perhaps under-researched feature of many rare diseases. Through our work in a resource center for rare diseases, we frequently encounter clients who report experienced fatigue as a serious problem in their daily life. Research has also shown that people with rare diseases often meet health professionals who lack knowledge and understanding of their diagnosis and symptoms [1, 2]. There is also a risk that health professionals do not pay attention to fatigue because it is overshadowed by other, possibly life-threatening disease symptoms. Moreover, there is a risk that by ascribing all symptoms to the rare disease; other medical causes of fatigue may be overlooked.

In Europe, a rare disease is defined as any disease affecting less than one person in 2000 people [3]. There are between 6000 and 7000 different rare diseases known today, approximately 70% are genetic in origin and 70% with childhood onset [4]. In 2016 the Orphanet database listed 3551 different rare genetic diseases [5]. Including all rare diseases in this review would be difficult. In order to capture the characteristics of fatigue research in a broad range of rare diseases we wanted to include diseases both genetic and not genetic of origin, diseases of child and of adult onset, and diseases with both visible and non-visible disabilities. We therefore chose to include a selection of diseases in the following groups of diseases according to the Orphanet classification of rare diseases [6]: rare developmental defects or anomalies during embryogenesis (e.g. spina bifida and congenital limb deficiency), and rare genetic diseases (e.g. Marfan syndrome, osteogenesis imperfecta, Duchenne muscle dystrophy and haemophilia).

Fatigue is a complex phenomenon lacking a clear and unanimous definition or overarching theory [7]. According to Kluger et al. [8] the lack of a clear definition of fatigue may impose a problem, also because many studies fail to explain how they define fatigue. Zwarts et al. [9] divide fatigue into physiological fatigue and experienced fatigue. They define physiological fatigue as a loss of the will-powered ability to produce power or energy under training and physical activity. Physiological fatigue is often investigated by testing muscle function and physical endurance.

Experienced fatigue is often also called subjective fatigue or perceived fatigue [9, 10], and can be defined as “an overwhelming sense of tiredness, lack of energy and feeling of exhaustion; mental, physical or both” [9, 11]. According to White et al. [12], physiological fatigue can contribute to subjective experienced fatigue, but people can experience subjective fatigue in the absence of physiological factors and physiological fatigue does not necessarily result in subjective fatigue. The scope of this review is experienced fatigue in persons with rare diseases.

Experienced fatigue is not specific to people with rare diseases. Research shows a high prevalence of experienced fatigue in adults with many different diseases and chronic conditions [11], and also in general populations [10, 13]. Research on experienced fatigue in general has been scarce across population groups and conditions. A review of published articles on fatigue and pain published between 2002 and 2011 showed an increase in publications on both domains, but fatigue represented only 15% of these publications, while pain was the focus of 85% [7]. Nonetheless, research shows that experienced fatigue can significantly impair adults’ ability to work [14] and their quality of life [15]. Although research on experienced fatigue in children is reported to be limited [16], a high prevalence of fatigue has been described both in children with physical disabilities [17], with chronic health problems [18], and in general populations [19]. Fatigue can reduce children’s participation in school and daily activities [16, 18], and their quality of life [16, 20]. Research in chronic diseases describe multiple associated or contributing factors to experienced fatigue (e.g. sleep disorders, pain, reduced physical activity, depression, and pharmacotherapy) [21, 22].

Being able to understand and accept experienced fatigue has been shown to be an important factor in enabling patients to manage and live with fatigue [16, 23]. Experienced fatigue has been described as a challenge in everyday life for people with some rare diseases [1, 24–26]. In research setting priority partnerships between patients and researchers, experienced fatigue has also been determined as one of the ten most important research questions in several rare disorders: rare inherited anemias [27], rare bleeding disorders [28], rare musculoskeletal disorders [29], and mitochondrial diseases [30]. This indicates a need for more knowledge about the status of fatigue research in rare diseases.

When investigating experienced fatigue, research questions used in evidence-based health practice [31] are relevant. Questions like: 1. How common is the condition (i.e. prevalence), 2. Which factors contribute to fatigue (i.e. associated, correlated or predicting factors), 3. What are reported interventions and intervention effects on experienced fatigue, 4. How can prevalence, associations and changes in experienced fatigue be investigated (i.e. development or validation of diagnostic methods), 5. How do patients describe their views and opinions of living with experienced fatigue (i.e. meaning)?

The first four questions are often investigated by asking the patient their opinion with some kind of quantitative questionnaire, either study-specific questions [32], or some kind of standardized fatigue instrument [33]. Standardized fatigue instruments range from unidimensional scales like visual analogue fatigue scales to multidimensional fatigue scales (e.g. Multidimensional Fatigue Inventory) [32]. Health-related quality of life (HRQoL) questionnaires, like Short Form 36 (SF36), have also been used to measure fatigue [34]. However, there are reports showing that SF36 may not capture higher levels of fatigue [33, 35].

The fifth question concerning how patients themselves describe, perceive and cope with experienced fatigue are often investigated with different qualitative methods such as individual or focus group interviews [36].

According to the International Rare Diseases Research Consortium (IRDIRC), measuring patient-reported outcomes is especially challenging in people with rare diseases [37]. The main challenge is to agree on the relevant aspect to measure for different rare diseases and for different age groups. It is also a statistical challenge to construct reliable measurement methods in small patient groups [37]. We think this also applies for experienced fatigue. Increasing knowledge about methods used to study experienced fatigue in persons with rare disorders can improve the quality and usefulness of further studies.

To our knowledge, an overview of the characteristics of fatigue research in children and adults with rare diseases is lacking. Our impression is that many people with different rare diseases experience fatigue and that research on this topic is scarce.

A baseline for further studies is to have an overview of the characteristics of existing research. This may include an overview of how studies define and describe experienced fatigue, the amount of primary research studies versus secondary studies (systematic reviews), and if investigation of experienced fatigue is primary or secondary aim. Furthermore an overview of characteristics of investigated patient populations, the amount of studies investigating different research questions and what kind of methods that have been used to investigate experienced fatigue in rare diseases may be of importance. Therefore the aims of this scoping review were:

- To systematically identify, map and describe the characteristics of existing research investigating patient-reported experienced fatigue in a selection of diseases in rare developmental defects or anomalies during embryogenesis and rare genetic diseases.
- To identify research gaps and point to research agendas related to experienced fatigue in people with rare diseases.

## Methods

### Study design and research questions

A scoping review methodology was applied because this is a suitable method for mapping findings from a research area that is heterogeneous in methods or disciplines [38]. Scoping reviews are also suitable for examining the extent, range, variety and characteristics of evidence on a topic, and also for identifying research gaps to aid future research [38–40]. The scoping review method is still new and is different from systematic reviews in the way that is does not provide an assessment of methodological quality of included studies [40]. This scoping review was performed according to the Joanna Briggs Institute and Collaborating Centers’ guidance for conducting scoping reviews [40] and the PRISMA Extension for Scoping Reviews [39] (shown in Additional file 1). A protocol for this review is available on request.

The review was guided by the question “What are the characteristics of research on experienced fatigue in rare developmental defects or anomalies during embryogenesis and rare genetic disorders?” Our specific research questions were:

1. How is the concept of experienced fatigue defined by the study authors? (i.e. in the introduction to the article).
2. What is the extent of secondary research articles (i.e. systematic reviews) versus primary research articles describing experienced fatigue in different rare diseases?
3. How much focus is given to experienced fatigue in the studies? (i.e. experienced fatigue primary or secondary aim/outcome),
4. What are the characteristics of study populations? (i.e. types of diseases, sample sizes, adults/children), and when and where have these studies been carried out? (i.e. publication year and country of participants).
5. How many studies investigate the different types of research questions on experienced fatigue? (i.e. prevalence and associations, treatment effects, diagnostics (development or validation of assessment methods), and views and experiences).
6. What types of assessment methods have been used to investigate experienced fatigue in persons with rare diseases? (i.e. study-specific or standardized fatigue questionnaires and qualitative interview methods)

#### Eligibility criteria

This scoping review included secondary and primary research studies aiming to investigate experienced (or subjective or perceived) fatigue in adults and children with rare diseases, both children’s report and parent’s proxy report of the child’s fatigue was included. We included articles that either stated in the article that they aimed to investigate experienced (or subjective or perceived fatigue), or articles that used standardized instruments for measuring experienced fatigue. Table 1 presents inclusion and exclusion criteria for this review.

**Table 1:** Inclusion and exclusion criteria

	Included	Excluded
*Population of interest*	Studies with participants from any country Both children and adults in a selection of rare diseases in the Orphanet rare disorder classification groups: Rare developmental defects / anomalies during embryogenesis and rare genetic diseases Studies including a broader population were included if: (a) presenting separate data on persons with a diagnosis in one of the included diagnostic groups, (b) the mixed populations included ≥ 80% of the study population with a diagnosis in one of the included diagnostic groups	Studies of experienced fatigue in common diseases and other rare diseases than the included diagnostic groups Studies with broader populations not presenting separate results for the included rare disorders or not including ≥ 80% of the study population with a diagnosis in one of the included diagnostic groups
*Publications relevant for inclusion*	Studies published in peer-reviewed journals: - primary research studies with one aim of investigating patient’s experienced fatigue, or using outcome measures for experienced fatigue - secondary research studies like systematic reviews giving data on experienced fatigue	Case-studies with ≤ 5 participants, Conference abstracts, posters, reports, book-chapters, unpublished data (grey literature), study protocols Expert opinions, guidelines and non-systematic reviews
*Topic of interest*	Studies presenting data on patient-reported experienced (or subjective or perceived) fatigue in both children and adults with one of the defined rare diseases. For children both children’s report and parent’s report of the child’s fatigue was included	Studies of: - physiological (muscular) fatigue/muscular endurance - caregiver or parent’s report of their own fatigue in caring for a child with a rare disease - medical professionals’ views of patients with rare diseases’ most important symptoms - fatigue as adverse effect in medication intervention studies, as this is primarily a temporary effect - fatigue reported as a symptom in studies of patients clinical characteristic (i.e. from patient records) Studies primarily investigating quality of life and reporting results of vitality using QoL instruments (e.g. SF-36), as these measures may not capture severe fatigue
*Languages*:	English, German, Danish, Norwegian or Swedish	

### Systematic searches

A research librarian (HS) performed systematic searches on 30 March 2020 in the following databases: MEDLINE (OVID), CINAHL (EBSCO), Embase (OVID), PsycINFO (OVID), AMED (OVID), Cochrane Database of Systematic Reviews, Cochrane Central Register of Controlled Trials, SveMed + , Scopus, and the following Web of Science databases: Science Citation Index Expanded, Social Sciences Citation Index, Arts & Humanities Citation Index, Conference Proceedings Citation Index-Science, Conference Proceedings Citation Index Social Science & Humanities, Emerging Sources Citation Index.

We searched for a combination of subject headings (where applicable) and text words for rare diseases, with the main search words rare disease or rare disorder and a selection of rare diseases in the diagnostic groups rare developmental defects or anomalies in embryogenesis and rare genetic diseases. We also searched for a combination of subject headings (where applicable) and text words for fatigue. See Additional file 2 for full search strategies. In addition, we hand searched the reference lists of included studies. No restrictions were made on publication dates.

### Selection of publications

All review steps were performed by two authors. We divided the review authors into two teams (TB/HJ and TB/GV), each evaluating half of the references. The Rayyan software [41] was used for screening of articles, and the review authors were blinded for each other’s decisions. When agreement was not reached, conflicts was resolved through discussion with a third review author (GV/HJ), using the inclusion and exclusion criteria (Table 1). The screening was done in two steps. In the first step abstracts and titles were reviewed and all articles clearly not meeting inclusion criteria were excluded. In the second step full-texts was collected and reviewed for the remaining articles.

### Data extraction

One researcher extracted data into a priori form, and another checked the accuracy. The following data was collected from each article: Bibliographic data, nationality/country of participants, study aim, participant data (number, diagnosis, gender, age), which research questions on experienced fatigue the study investigated (prevalence and/or associations, diagnostics- development/validation of fatigue assessment tools, intervention effects, patient’s views and experiences), study design, if study authors reported how they defined experienced fatigue and how much focus the study had on experienced fatigue (primary or secondary aim/outcome), and finally how patient’s experienced fatigue was investigated (quantitative methods: study-specific or standardized fatigue questionnaires, or qualitative methods). From papers that included other populations or themes in addition to fatigue in a rare disorder, we selected and presented data on fatigue in the rare disorder only.

### Summarizing and presentation of findings

All included articles were sorted according to diagnostic group and specific diagnosis using EndNote. Extracted data according to the priori form is presented for each diagnostic group and diagnosis in Additional file 3. A full reference list of included articles is presented in Additional file 4. A list of excluded articles with reason for exclusion is given in Additional file 5. Data is presented in a descriptive manner using text and figures.

## Results

**Fig. 1:**
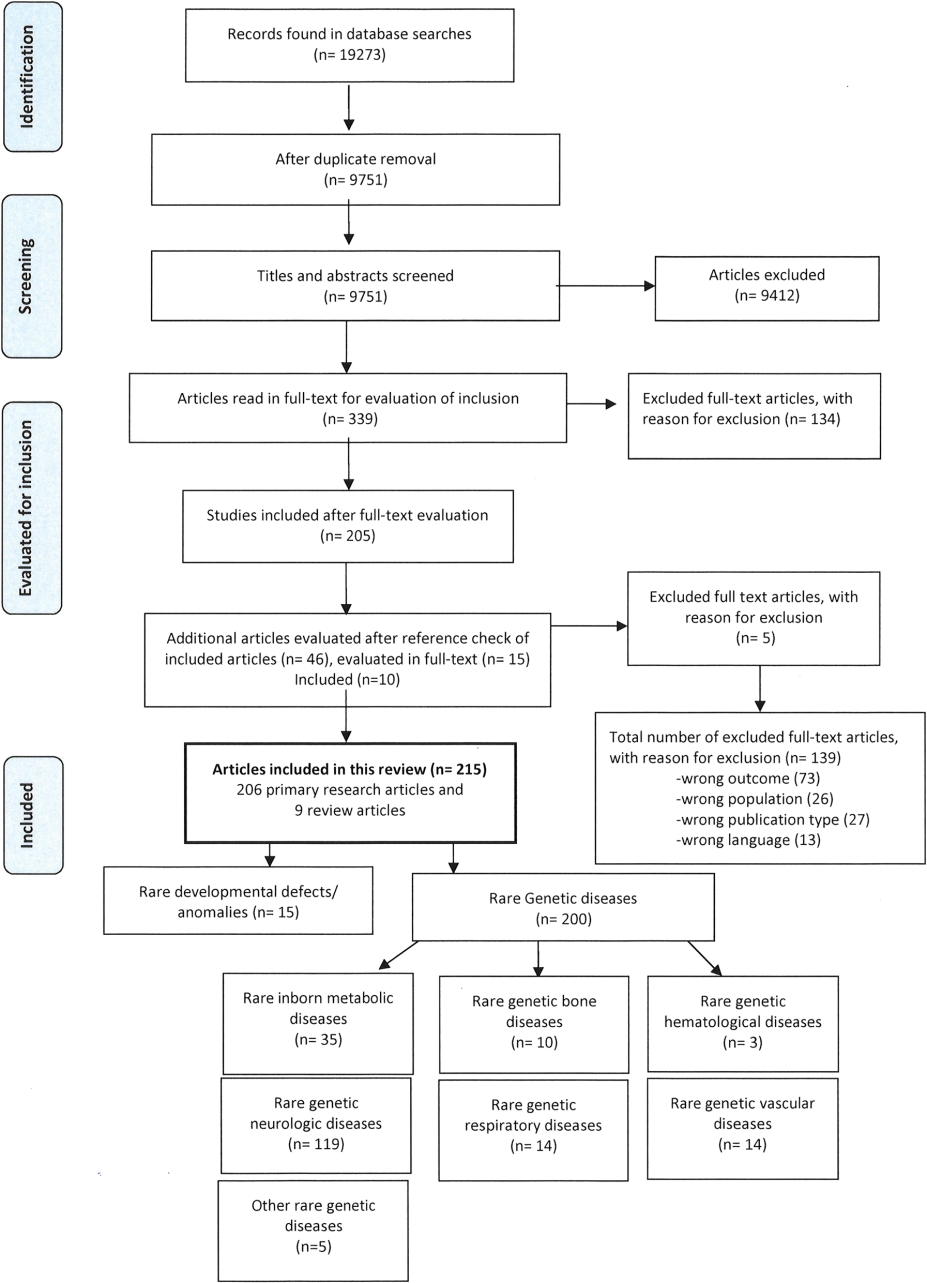
Flowchart of search, screening and inclusion process

The searches resulted in a total of 19,273 hits, which were reduced to 9751 after deduplication [42]. Of these 9412 references were excluded after screening of the titles and abstracts. After full-text reading of the remaining 339 references, 205 articles were included. Ten additional articles were included after reference-check of included articles, giving a total of 215 included articles; 9 secondary research articles (reviews) and 206 primary research articles (Additional files 3 and 4). Figure 1 show a flowchart of the screening and inclusion process.

### Definition of experienced fatigue

Only 29 of 215 articles describe in the introduction how they defined experienced fatigue, one systematic review and 28 primary research articles. They all had investigation of experienced fatigue as a primary aim or outcome. A wide variation of definitions, with references to many different sources were used. In 15 articles authors used the following definition (or a variation of it): “Fatigue can be defined as an overwhelming sense of tiredness, lack of energy and feeling of exhaustion, mental, physical or both” [11]. Other examples illustrate very different ways of defining experienced fatigue, like for instance: “Fatigue is defined clinically as a decline in performance during sustained activity, and can be associated with performance on both motor and cognitive tasks [43]. Another definition used was: “…fatigue is often difficult to describe. To some patients, it represents the inability to perform day to-day activities. To others, it is a combination of tiredness, decreased muscle activity, and lower mood. Therefore, several terms are often used to describe fatigue, such as lack of energy, tiredness, and muscle weakness” [44]. See Additional file 3 for details on each article.

### Secondary research articles, characteristics and investigated research questions

Nine reviews were included, all about rare genetic diseases: six on rare inherited metabolic diseases, two on rare genetic neurologic diseases, and one on rare genetic respiratory diseases. Only three reviews had investigation of experienced fatigue as a primary aim/outcome, and only one were about research on children (Additionals files 3 and 4). The reviews covered all types of research questions: three articles reviewed diagnostic methods for investigating fatigue by doing a validation or appraisal of outcome measures for a specific rare disease (lysosomal storage diseases, myotonic dystrophy type 1, and pyruvate kinase deficiency) [45–47]. Two articles reviewed intervention effects on fatigue in disease-specific medication interventions, (in paroxysmal nocturnal hemoglobinuria and peripheral neuropathy, including the rare disease Charcot Marie-Tooth) [12, 48]. Two articles reviewing literature on the burden of living with Pompe disease gave some data on prevalence of fatigue and associated factors [49, 50]. One article reviewed patient’s experiences of living with cystic fibrosis [51], and one article on Gaucher disease gave data on all research questions [52].

### Primary research articles, characteristics, research questions and methods used to investigate experienced fatigue

We included 206 primary research articles presenting data on experienced fatigue. In only 79 articles (38%), investigation of experienced fatigue was a primary aim/outcome (Additional files 3 and 4).

#### Characteristics of study populations; diagnoses, sample sizes, age groups, publication year and country of participants

Only 15 articles about experienced fatigue were included in the group rare developmental defects or anomalies during embryogenesis, encompassing ten different diagnoses. A total of 191 articles investigated experienced fatigue in approximately 35 different diagnoses, in seven groups of rare genetic diseases (Table 2). Many articles may have been from the same study, but this could not be elucidated exactly as not all articles reported this clearly. A detailed description of data extraction from each article according to the diagnostic groups shown in Table 2, is presented in Additional file 3.

**Table 2:** Diagnostic groups and diagnoses reported on in included articles

Rare developmental defects/ anomalies during embryogenesis	Number of articles	Rare genetic diseases*	Number of articles
		Diverse rare genetic diseases**	
Arthrogryposis	1	Rare genetic diseases, mixed populations	2
Congenital upper-limb deficiency	2	Familial mediterranean fever	1
Hydrocephalus	1	Hereditary angio-oedema	1
Klinefelter syndrome	1	Primary immunodeficiency disorder	1
Neurofibromatosis	3	*Rare inborn metabolism diseases*	
Noonan syndrome	1	Barth syndrome	1
Silver-Russel syndrome	1	Fabry disease	4
Spina Bifida	1	Familial chylomicronemia	1
Turner syndrome	2	Gaucher disease	3
Velocardiofacial syndrome	2	Mevalonate kinase deficiency	1
		Morquio A syndrome, mucopoly-saccharidosis Iva	1
		Mucopolysaccharidosis VII	3
		Paroxysmal nocturnal hemoglobinuria	3
		Pompe disease	8
		Porphyria	4
		*Rare genetic bone diseases*	
		McCune-Albright syndrome	1
		Multiple osteochondroma	1
		Osteogenesis imperfecta	5
		X-linked hypophosphatemia	3
		*Rare genetic hematological diseases*	
		Congenital (hereditary) Thrombotic Thrombocytopenic purpura	1
		Haemophilia	1
		Severe aplastic anemia	1
		*Rare genetic neurologic diseases*	
		Charcot-Marie-Tooth disease (hereditary motor and sensory neuropathy type I)	11
		Duchenne muscular dystrophy	8
		Facioscapulohumeral muscular dystrophy	10
		Hereditary ataxias	6
		Hereditary spastic paraplegia	4
		Limb-girdle muscle dystrophy	1
		Mitochondrial diseases	9
		Muscular dystrophies, mixed populations	26
		Myotonic dystrophies	40
		Oculopharyngeal muscular dystrophy	2
		*Rare genetic respiratory diseases*	
		Cystic fibrosis	10
		Lymphangioleiomyomatosis	3
		*Rare genetic vascular diseases*	
		Ehlers-Danlos syndrome, rare genetic subtypes	3
		Marfan syndrome	11

*Rare genetic diseases are sorted into diagnostic groups according to the Orphanet classification

**Articles including several groups of genetic diseases and genetic diseases not fitting into any of the other diagnostic groups

In primary research articles the size of study-populations varied from five to 2366. In 85% of the articles the study population was 200 or less, with a median of 44 participants. Only 27 primary research articles reported data on experienced fatigue in children, 16 included both children and adults, and 163 included adults only.

The included articles were published between 1988 and 2020, 156 articles (76%) after 2010. In 15 articles participants from several countries were included. The majority of articles included participants from Europe (140 articles), North-America (71), South-America (4), Asia (3), Africa (2), and Middle-East (1), see Additional file 3 for details on each article.

#### Number of studies investigating different research questions

**Fig. 2:**
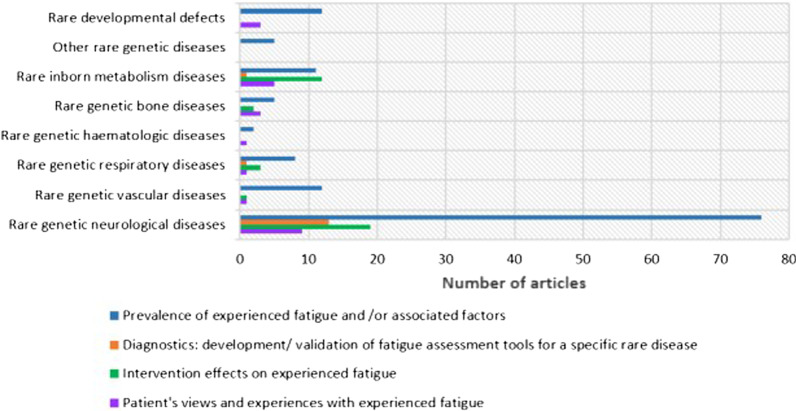
Map of investigated research questions on fatigue in rare disorders. Number of primary research articles according to research questions in each diagnostic group

The research questions investigated most frequently was prevalence and/or associations to experienced fatigue (in 131 articles). Development or validation of fatigue assessment tools for a rare disease (diagnostics), was the least frequent (15 articles). Figure 2 shows a map of investigated research questions on experienced fatigue in the different diagnostic groups.

Articles reporting prevalence and or associations to experienced fatigue were found in all diagnostic groups, the majority (76 articles) in rare genetic neurologic diseases (Fig. 2). Most articles report data on both prevalence and associations, some only on prevalence and others only on associations. Many different methods were used to investigate prevalence of experienced fatigue (see Additional file 3).

**Fig. 3:**
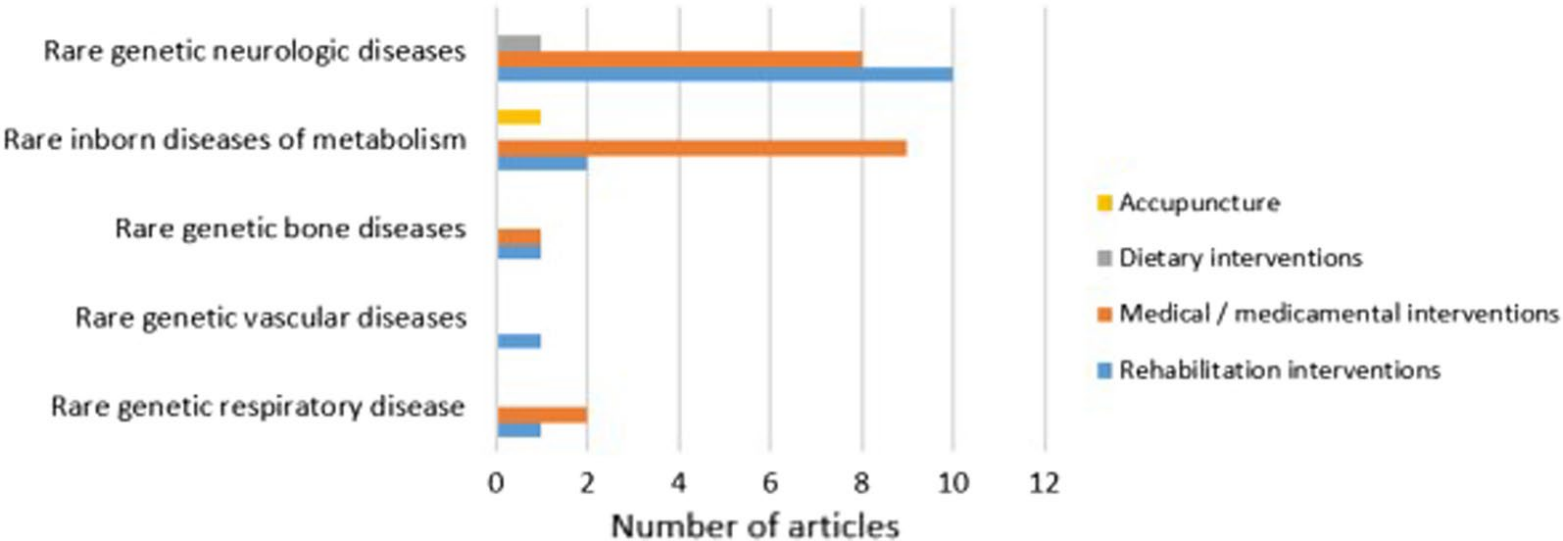
Articles on intervention effects on experienced fatigue in rare disorders. Number of primary research articles reporting different interventions for experienced fatigue in rare genetic diseases

Intervention effects on experienced fatigue was reported in 37 articles. Rare genetic neurologic diseases and rare inborn metabolic diseases had the highest number of articles on intervention effects (Fig. 2). We found no articles reporting intervention effects on experienced fatigue in rare developmental defects or anomalies during embryogenesis. The effect of disease-specific medical or drug interventions on experienced fatigue was reported in 20 articles (Fig. 3). Fifteen articles reported changes in experienced fatigue after some type of rehabilitation intervention: Exercise and or physiotherapy, either alone or in combination with cognitive behavioral therapy or self-management programs. One study investigated effect of a dietary intervention and one effect of acupuncture on disease symptoms. More details in Additional files 3 and 4.

The research question on diagnostics (validating or developing fatigue measures for a specific rare disease), was investigated in 15 articles. One in an inborn metabolic disease, one in a rare respiratory disease, and 13 in rare genetic neurologic disorders. Nine articles investigated validity and or feasibility and reliability of different generic fatigue measures in a rare disease (e.g. PROMIS, Fatigue Severity Scale, PedsQL multidimensional fatigue scale). Six articles described development and investigation of disease-specific outcome measures with fatigue scales (e.g. Chronic Respiratory Disease Questionnaire, Swallowing Quality of Life instrument, and Fatigue and Daytime Sleepiness Scale). More details in Additional files 3 and 4.

Patients with rare disease’s views and experiences concerning fatigue was reported in 23 articles (Fig. 2). Only two articles had investigation of patients’ experienced fatigue as primary aim. The remaining articles investigated experiences of living with the disease with experienced fatigue as one aspect. Only in four articles were investigation of children’s views and experiences included (more details in Additional files 3 and 4).

#### Quantitative methods used to investigate experienced fatigue

In 183 articles some form of quantitative questionnaire was used to investigate prevalence and/or associations or changes in experienced fatigue due to intervention.

**Fig. 4:**
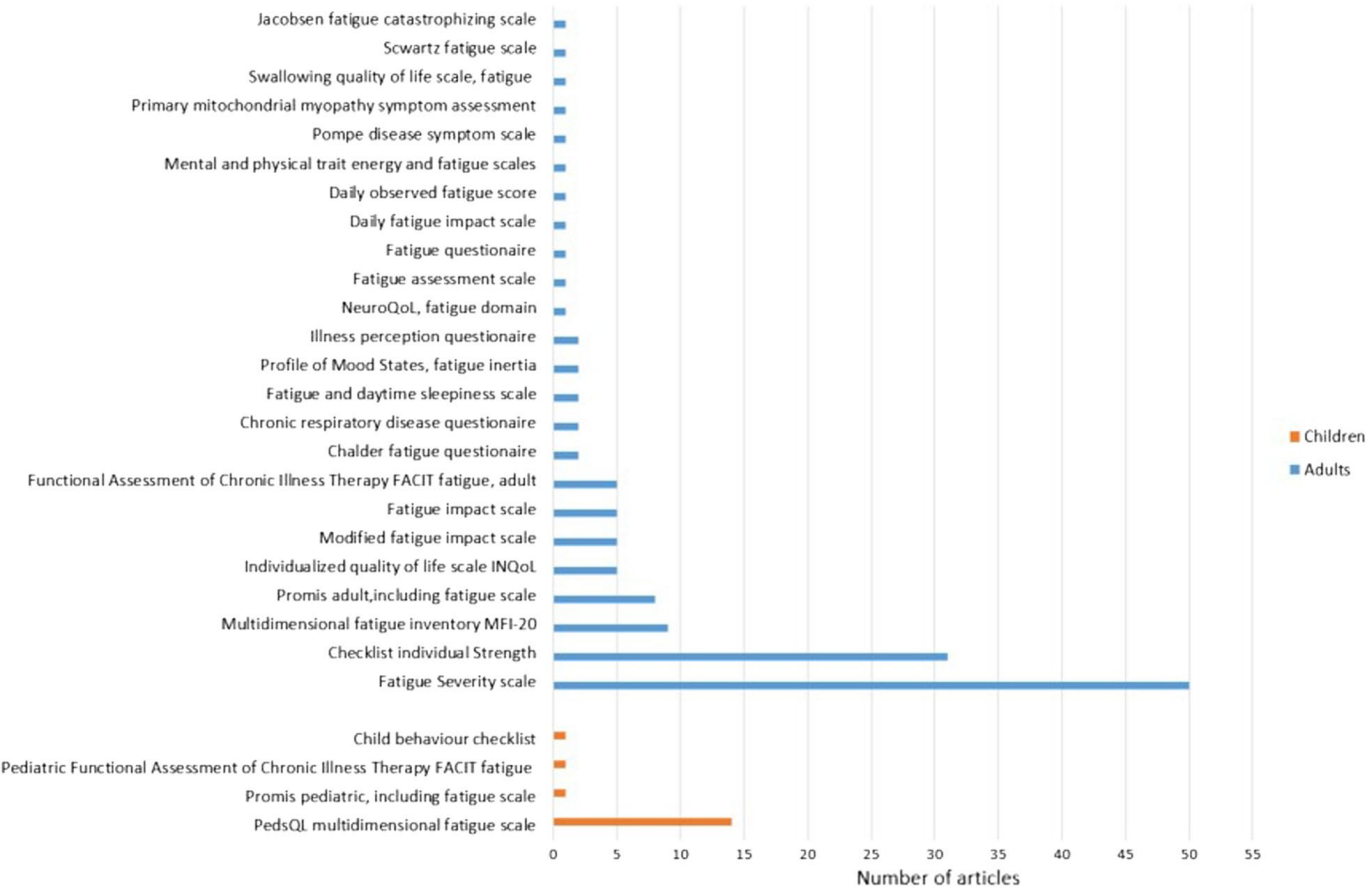
Standardized fatigue questionnaires in rare disorders. Standardized questionnaires used to assess fatigue in included primary research articles. Many articles reporting use of more than one standardized instrument

In 39 articles a study-specific questionnaire was used. In 13 of these a visual analogue or numeric scale was used, asking respondents to rate their fatigue from 0 to 10 with a study specific instruction if it was to rate importance, intensity, worsening etc. A standardized questionnaire was used in 144 articles, either a generic fatigue instrument or a disease specific outcome measure with a fatigue scale. Twenty-eight different standardized instruments were used, 19 only in one or two articles. Fatigue Severity Scale was the most frequent instrument used in adults, PedsQL multidimensional fatigue scale in children (Fig. 4).

There was a wide variation in use of standardized questionnaires in all the different diagnostic groups, such as in rare genetic neurologic diseases were 25 different standardized fatigue questionnaires were used (see Additional file 3 for details on each article).

#### Qualitative methods used to investigate experienced fatigue

Twenty-three articles used qualitative methods to investigate patients’ views and experiences either on coping with fatigue, or on living with the disease with fatigue described as one aspect. A range of different methods were used for gathering information on patient’s views and experiences: Focus group interviews (5 articles), individual interviews; face-to face or by telephone (15 articles), and analysis of recordings from panel discussions, group sessions or online open consultation (3 articles). More details in additional files 3 and 4.

## Discussion

This scoping review found 215 articles reporting data on experienced fatigue in a selection of diseases in the diagnostic groups’ rare developmental defects or anomalies in embryogenesis and rare genetic diseases. This overview of the characteristics of existing research, shows that there is a large variety of research on experienced fatigue in people with rare diseases. The majority of articles investigated the research questions prevalence and or associations to fatigue, few publications dealt with research questions like intervention effects and patients’ experiences of fatigue. Another important finding was the large variation in how experienced fatigue was defined and also in how it was measured.

### Definition of experienced fatigue

Only 29 of 215 articles described in the introduction how they defined experienced fatigue. This may have many causes, the assumption that the term fatigue is clearly known may be one. Another reason may be the limited word count in scientific publications, and many articles probably had a clear definition of which aspects of fatigue they investigated although it was not explained in the article.

Our findings are perhaps not surprising, and in line with Kluger et al. [8] who describe that many studies of fatigue in neurological diseases fail to define fatigue, and that those who do use many different definitions. This was evident also in our findings, illustrating that although authors stated that they either investigated experienced fatigue or used standardized questionnaires for experienced fatigue, there was a very large variation in how they defined fatigue. A possible challenge when fatigue is not defined in a study is that it may not be clear either to researchers or participants what the topic of interest is. This may lead to choosing outcome measures that don’t capture the topic of interest, or what is important to patients, or aren’t able to capture meaningful changes.

According to Friedberg et al. [7] the scientific status for fatigue research is still weak, and there is a lack of an overarching paradigm or theory guiding the study of fatigue. Kluger et al. [8] suggest as a minimum that studies should document how perceptions of fatigue is defined, operationalized and distinguished from related phenomena. We think the understanding and definition of experienced fatigue may be an important topic for further research both in rare diseases and more common diseases.

### Characteristics of included articles and study populations

Our finding of a total of 215 articles on experienced fatigue may seem like a large number. However, in only 38 percent of included articles investigation of experienced fatigue was a primary aim. Also, the included articles describing experienced fatigue covered approximately 45 different rare diseases, for many diseases with only one article. This may reflect true differences in occurrence and importance of experienced fatigue between patient groups, but may also reflect the amount of work done overall in that disorder. It might also be a difference in interest in studying experienced fatigue.

Our findings support the findings from James Lind Alliance partnerships between researchers and patient organization underlining fatigue as one of the 10 most important research questions in several rare disorders [27–30]. There may be reason to investigate experienced fatigue more extensively both in diseases where we found studies and in diseases where we did not.

As expected when describing research in rare diseases many articles had relatively few participants, 85% had a study population of 200 or less, with a median of 44. Rath et al. [53], describe several challenges in rare disease research, for instance: recruitment issues (related to both the limited number of patients and the challenge of correctly diagnosing and identifying rare disease patients), incomplete understanding of natural history of many diseases and need for more sensitive outcome measures. This underlines the need both for multinational cooperation, but also the importance of synthesizing and reviewing the research that already exists on experienced fatigue in rare diseases.

Despite the fact that approximately 70% of rare diseases are pediatric in onset [4], we found only twenty-seven primary research articles reporting data on fatigue in children, and sixteen reporting data both on children and adults. This may reflect that experienced fatigue develop later in life and maybe is related to age-related changes. Associations between experienced fatigue and age has been found in general populations [10, 13], but not in rare disorders like Marfan syndrome [54], congenital limb deficiencies [55], myotonic dystrophies [56] and Pompe disease [57]. On the other hand it may also imply that there is a need for more research on experienced fatigue in children with rare diseases. A review of fatigue in children with chronic health problems found that fatigue was one of the most important symptoms reported by children [16]. Therefore further research on experienced fatigue in children with rare diseases is needed.

### Secondary versus primary research articles

The finding of only nine secondary research articles (systematic reviews), indicates that there is a research gap on the summary and critical evaluation on existing research. Systematic reviews are viewed as essential for both healthcare providers and policy-makers to guide clinical practice and develop trustworthy clinical guidelines. Systematic reviews are also essential to establish what is already known and what is needed in further research [58]. The included 206 primary research articles covered many different diseases, in some rare diseases there were several articles investigating fatigue (e.g. myotonic dystrophies with 40 articles). This may give opportunity and reason to perform systematic reviews on fatigue research in a specific disease or disease group. Our overview of articles covering different research questions also point to areas were systematic reviews of existing research may be indicated, for instance on prevalence of fatigue in different rare diseases.

### Investigated research questions on experienced fatigue

#### Prevalence and associations

The most common research question investigated was prevalence and/or associations to experienced fatigue. This was investigated in 131 of 206 primary research articles, and in two of the included review articles. However, there were wide variations in the number of articles investigating prevalence and/or associations to experienced fatigue in different diseases and diagnostic groups, in sample sizes and also in methods used. Most articles on prevalence and associated factors were about adults. Rath et al. [53] highlight challenges related to rare disease research, like recruitment difficulties related to the challenges of diagnostics, finding the right patients and limited knowledge on clinical history and symptoms. This is also relevant for research on prevalence of fatigue and factors that can contribute to experience of fatigue. As shown in Additional file 3, in many of the included diseases only one or two articles were found giving data on prevalence and or/ associations. This makes generalization of findings from the study population to the whole disease population difficult. Therefore for many of the diseases we included, more primary research on both prevalence and associations to experienced fatigue is warranted. In diagnoses with several articles on prevalence and associations, for instance in cystic fibrosis and muscle dystrophies, a summary and evaluation of existing evidence may be advisable.

#### Intervention effects

Intervention effects on experienced fatigue was the second most investigated research question. However, with only 37 articles. The included articles were all about rare genetic diseases. We did not find articles on intervention effects on fatigue in the group rare developmental defects or anomalies during embryogenesis. The low number of articles imply that there is a need for more primary research on treatment options for experienced fatigue in persons with rare diseases. As experienced fatigue has been shown to have a significant impact on both adult’s [14, 15] and children’s [16, 20] daily living and quality of life, finding treatment alternatives seems imperative also in rare diseases.

The majority of included primary research articles (20 articles) studied medical/drug interventions for the disease. Two of the included review articles [12, 59], reviewed the literature on effects of medical intervention in paroxysmal hemoglobinuria and peripheral neuropathy. Experienced fatigue has been described as a multidimensional problem with need for interventions that address this multidimensionality [8, 21, 60]. In the 15 included articles on different rehabilitation interventions, several articles described interventions on multiple modalities like physical exercise combined with psychosocial support and cognitive-behavioral therapy. Systematic reviews indicate that non-pharmacological and multidisciplinary interventions have an effect on experienced fatigue in different groups, like rheumatoid arthritis [61], older adults [62], and multiple sclerosis [63]. This implies a need for research exploring the effect of multimodal rehabilitation interventions on experienced fatigue, both in children and adults with rare diseases. A review of existing studies on multimodal rehabilitation interventions in different rare diseases may also give guidance for further research and clinical practice.

#### Diagnostics; validation or development of fatigue assessment tools, and quantitative methods used to investigate experienced fatigue

We found only fifteen articles investigating validation or development of fatigue assessment tools for a rare disease patient group. This is in contrast to existing recommendations that emphasize that when choosing patient reported outcome measures (PROMs) it is important to ensure cultural and linguistic validation, and also evaluate the appropriateness for patient, condition and therapy [64]. Even if an outcome measure is found valid in one patient group, it may not necessarily be valid or appropriate for another patient groups. Nine of the included articles investigated disease-specific validity of well-known fatigue measures like PROMIS, Fatigue Severity Scale and PedsQL multidimensional fatigue inventory. Some of these are generic measures and some designed for specific patient groups. The benefit of using generic PROMs are that they are comparable across diseases, and for some reference values to the general populations exist. However it is argued that generic measures may not be sensitive to disease specificities [64, 65]. On the other hand disease-specific measures pose the challenge that they can only make comparisons within the same patient group, and developing new PROMs are time and resource demanding. Some solutions suggested by Whittal et al. [64] is multinational collaboration with multisite data collection, and the use of generic and disease-specific PROMs in a complementary way. Our results also show that the use of many different generic and disease specific fatigue measures within the same diagnostic groups impedes comparisons across studies. Further research, both primary and secondary studies on diagnostic methods to evaluate experienced fatigue in rare diseases is recommended.

In 183 primary research articles some type of quantitative question/ questionnaire was used to investigate experienced fatigue. Most strikingly was the large variety of methods used, making comparisons between different diseases and different study populations challenging, for instance in rare genetic neurologic disorders where 25 different standardized questionnaires were used. On a positive note is the majority of articles using standardized questionnaires as opposed to study-specific questions, this is an improvement compared to an earlier systematic review of fatigue assessment methods in chronic conditions [32]. To overcome the challenge the use of many different assessment methods imposes, it has been proposed that multiple stakeholders like researchers, health professionals and patient organizations cooperate to create standardized sets of outcomes relevant for a rare disease. This enables agreement on what aspects that are important to measure, how it should be measured and how results should be interpreted [37, 53]. One such example can be found in the Key4OI project that has resulted in an international consensus on a standard set of outcome measures, including both disease specific and generic PROMs, for care and follow-up of persons with osteogenesis imperfecta [66]. There is a need for more primary and secondary research and collaboration on the choice, use and comparison of fatigue outcome measures in rare diseases.

#### Meaning of fatigue—patient experiences

The finding of only 23 articles using qualitative methods to investigate patients with rare diseases’ views and experiences concerning fatigue, implies a need for both primary and secondary research on this topic in rare diseases. Benjamin et al. [67] advocate the use of a wide range of methods to get input from patients with rare diseases, on which issues that are important to address in research and clinical trials. Morel et al. [37] state that research on disease impact and treatment benefits in rare disorders should incorporate patient-centered outcome measures that capture what matters to rare disease patients. In order to know which aspects of experienced fatigue that is important to understand, address and treat for a specific rare disease, we need to ask the patients. This can give a better platform for further research on understanding, investigating and treating fatigue. Studies [16, 23] have also shown that being able to understand and accept experienced fatigue may be an important factor in enabling patients to manage and live with fatigue. More evidence on patient’s descriptions on how they live with, understand and cope with experienced fatigue may also help patients with rare diseases to manage fatigue. Conducting a systematic review of patients’ descriptions of living with experienced fatigue across rare diseases may also provide a better insight of differences and similarities between diagnoses. A similar review of experienced fatigue in patients with long-term conditions found both similarities and differences across different chronic conditions [68].

#### Strengths and limitations

Comprehensive searches by a research librarian in a wide range of bibliographic databases, and the use of Rayyan for blinded evaluation between reviewers are strengths of this review. However, as fatigue is not a well-defined concept it was sometimes difficult to distinguish between physical and experienced fatigue as study authors did not always specify this. The use of specified inclusion criteria, and discussion in the review teams when disagreement was a strength and helped to resolve this problem, but we may have missed some relevant articles. Our search strategy provided the opportunity for a wide search on rare diseases and fatigue terms. However, the choice of not searching for all possible disease names may have contributed to us missing out on possibly relevant references.

We chose to focus the review on experienced fatigue in persons with a selection of rare diseases. We believe this restricted focus is a strength as it provided the opportunity to include a wide range of research on experienced fatigue in various rare diseases. However, the choice of not including studies mainly investigating quality of life may also have lost some relevant articles. The choice of not including other languages than English, German, Danish, Norwegian and Swedish languages may also have led to loss of relevant articles. The classification of rare diseases is challenging. The use of the Orphanet classification helped categorizing diagnoses into diagnostic groups, but as many diseases can be categorized into several diagnostic groups in the Orphanet classification we may have misplaced or overlooked some articles.

## Conclusion

This scoping review on characteristics of fatigue research in rare diseases, found a large variety of research on experienced fatigue. However, the minority of studies had investigation of experienced fatigue as a primary aim. An important finding was the large variation in how experienced fatigue was defined and also in how it was measured. The majority of articles investigated the research questions prevalence and or associations to fatigue, few publications dealt with research questions like diagnostics, intervention effects and patients’ experiences of fatigue. There is a need for more research on experienced fatigue in rare diseases, in both in children and adults. This includes both primary and secondary research: on prevalence and or associations to experienced fatigue, on treatment effects, on diagnostics (validation or development of assessment methods for experienced fatigue in rare diseases), and rare disease patients’ views and experiences regarding living with experienced fatigue. This review offers a basis for further research and development of clinical practice.

## Supplementary Information

**Additional file 1**. PRISMA-ScR Checklist.docx. Describes the steps in this article, according to the PRISMA-ScR Checklist.

**Additional file 2**. Search strategies.pdf. Search strategies in different databases for this review.

**Additional file 3**. Data extraction of included articles.pdf. Data is presented for each included reference. References are presented in alphanetical order in diagnsotic grups and diagnoses.

**Additional file 4**. Reference list of included articles.pdf. First, a list of all included articles is given, then references from some specific themes (secondary research articles – reviews, articles on diagnostics – development/ validation of fatigue assessment tools, articles on treatment effects, articles on patient’s views and experiences).

**Additional file 5**. Reference list excluded articles.pdf. List of excluded references with reason for exclusion.

## Data Availability

All data generated or analyzed during this study are included in this published article (and its additional files).
