# Supplementary material for: Experienced fatigue in people with rare disorders: a scoping review on characteristics of existing research
Source: Orphanet J Rare Dis. 2022 Jan 10;17:14. doi: 10.1186/s13023-021-02169-6 (PMC8751355; doi:10.1186/s13023-021-02169-6)
Supplement: Supplementary file 2 — Additional file 2. Search strategies.pdf. Search strategies in different databases for this review. [file 13023_2021_2169_MOESM2_ESM.pdf]

Additional file 2: Search strategies

All searches were performed on 30 March 2020

Ovid MEDLINE(R) ALL 1946 to March 26, 2020 (Ovid)

1	Rare Diseases/ or Osteogenesis Imperfecta/ or Exostoses, Multiple Hereditary/ or exp Fibrous Dysplasia of Bone/ or Achondroplasia/ or Marfan Syndrome/ or Ehlers-Danlos Syndrome/ or Loeys-Dietz Syndrome/ or exp Muscular Dystrophies/ or Glycogen Storage Disease Type II/ or exp Porphyrias/ or Hemophilia A/ or Hemophilia B/ or Cystic Fibrosis/ or exp Neural Tube Defects/ or exp Limb Deformities, Congenital/ or Charcot-Marie-Tooth Disease/ or Spastic Paraplegia, Hereditary/ or DiGeorge Syndrome/ or exp Neurofibromatoses/ or Turner Syndrome/ or exp Mitochondrial Diseases/ or Noonan Syndrome/ or Klinefelter Syndrome/	221112
2	((rare adj3 (disease* or disorder*)) or (orphan adj (disease* or disorder*)) or (osteogenesis adj imperfecta) or brittle bone disease* or fragilitas ossium or osteopsathyrosis or ((lobstein* or bruck*) adj (disease* or syndrome*)) or ((skeletal or fibrous) adj3 (dysplasia* or bone*)) or achondroplas* or Diaphyseal Aclasis or ((Hereditary or Familial or Multiple) adj2 (Exostos* or Chondrodysplas* or Osteochondroma*)) or Bessel-Hagen Disease or (Fibro* adj Dysplasia*) or Jaffe Lichtenstein* or (Marfan* adj2 (syndrome* or disease* or disorder* or abiotrophy)) or (Ehlers adj Danlos) or (Loeys adj Dietz) or (genetic adj3 aortic) or ((musc* or limb-girdle) adj dystroph*) or (Glycogen Storage Disease Type adj (II or "2")) or (glycogenos* adj2 (II or "2" or generali*)) or (Pompe adj (disease* or syndrome* or disorder*)) or porphyria* or (porphyrin adj (disorder* or disease* or syndrome*)) or hemophilia* or haemophilia* or ((cystic or pancreatic) adj2 fibros*) or mucoviscidosis or mucoviscoidosis or (pancrea* adj (fibrocystic or fibros* or cystic)) or myelomeningocele* or (neural tube adj2 defect*) or spina bifida* or (congenital adj3 (limb* or exremit*)) or ((limb* or exremit*) adj3 (deformi* or malform* or anomalit*)) or Arthrogryposis multiplex congenita or Amyoplasia* or (Charcot adj Marie) or (Roussy adj Levy) or Peroneal Muscular Atroph* or Hereditary Areflexic Dystasia* or ("hereditary motor and sensory" adj neuropat*) or (HMSN adj2 (I or II or 1A or 1B or "5" or V)) or HMSN1A or HMSN1B or ((Strumpel* or Struempel*) adj1 (disease or syndrome)) or (familial spastic adj (paralysis or paraparesis or paraplegia)) or (dejerine sottas adj (syndrome or disease)) or (myoton* adj2 (dystroph* or atrophica* or myopathy*)) or (Steinert* adj disease*) or Ricker Syndrome* or PROMM* or (Hereditary adj3 Spastic Paraplegia*) or Spastic Paraplegia Hypertrophic Motor Sensory Neuropathy or "CMT with Pyramidal Features" or (Spastic Paraplegia adj2 ("2" or II)) or SPG2 or (rare adj3 hereditary ataxia*) or ((DiGeorge or Di George) adj (syndrome* or sequence or anomaly)) or ((velocardiofacial or Velo Cardio Facial or 22q11 or vcf or pharyngeal pouch or Thymic Aplasia or Sedlackova or Shprintzen) adj2 syndrome*) or "Autosomal Dominant Opitz G Bbb Syndrome" or "Conotruncal Anomaly Face Syndrome" or Catch22 or neurofibromatos* or recklinghausen* or (multiple adj1 neurofibroma*) or ((Turner* or Ullrich* or XO or 45X) adj3 (syndrome* or disease* or state	268903

	or status or stigma*)) or (Gonadal Dysgenesis adj XO) or Monosomy X or (Bonnevie Ullrich adj (syndrome* or status)) or (mitochondrial adj (disease* or disorder*)) or respiratory chain deficienc* or oxidative phosphorylation deficienc* or (Noonan* adj3 (syndrome* or disease*)) or ((Klinefelter* or XXY or XXYY or XXXXY) adj3 (syndrome* or trisom* or disease*))).tw,kf.	
3	1 or 2	351928
4	Fatigue/	28165
5	(fatigue or lassitude or tiredness or vitality or exhaust*).tw,kf.	157577
6	4 or 5	164453
7	3 and 6	2281

Embase Classic+Embase <1947 to 2020 March 27> (Ovid)

1	rare disease/ or osteogenesis imperfecta/ or hereditary multiple exostosis/ or exp fibrous dysplasia/ or achondroplasia/ or Marfan syndrome/ or Ehlers Danlos syndrome/ or Loeys Dietz syndrome/ or exp muscular dystrophy/ or glycogen storage disease type 2/ or exp porphyria/ or exp hemophilia/ or cystic fibrosis/ or exp neural tube defect/ or exp limb malformation/ or hereditary motor sensory neuropathy/ or DiGeorge syndrome/ or exp neurofibromatosis/ or exp disorders of mitochondrial functions/ or Noonan syndrome/ or exp Klinefelter syndrome/	413608
2	((rare adj3 (disease* or disorder*)) or (orphan adj (disease* or disorder*)) or (osteogenesis adj imperfecta) or brittle bone disease* or fragilitas ossium or osteopsathyrosis or ((lobstein* or bruck*) adj (disease* or syndrome*)) or ((skeletal or fibrous) adj3 (dysplasia* or bone*)) or achondroplas* or Diaphyseal Aclasis or ((Hereditary or Familial or Multiple) adj2 (Exostos* or Chondrodysplas* or Osteochondroma*)) or Bessel-Hagen Disease or (Fibro* adj Dysplasia*) or Jaffe Lichtenstein* or (Marfan* adj2 (syndrome* or disease* or disorder* or abiotrophy)) or (Ehlers adj Danlos) or (Loeys adj Dietz) or (genetic adj3 aortic) or ((musc* or limb-girdle) adj dystroph*) or (Glycogen Storage Disease Type adj (II or "2")) or (glycogenos* adj2 (II or "2" or generali*)) or (Pompe adj (disease* or syndrome* or disorder*)) or porphyria* or (porphyrin adj (disorder* or disease* or syndrome*)) or hemophilia* or haemophilia* or ((cystic or pancreatic) adj2 fibros*) or mucoviscidosis or mucoviscoidosis or (pancrea* adj (fibrocystic or fibros* or cystic)) or myelomeningocele* or (neural tube adj2 defect*) or spina bifida* or (congenital adj3 (limb* or exremit*)) or ((limb* or exremit*) adj3 (deformi* or malform* or anomalit*)) or Arthrogryposis multiplex congenita or Amyoplasia* or (Charcot adj Marie) or (Roussy adj Levy) or Peroneal Muscular Atroph* or Hereditary Areflexic Dystasia* or ("hereditary motor and sensory" adj neuropat*) or (HMSN adj2 (I or II or 1A or 1B or "5" or V)) or HMSN1A or HMSN1B or ((Strumpel* or Struempel*) adj1 (disease or syndrome)) or (familial spastic adj (paralysis or paraparesis or paraplegia)) or (dejerine sottas adj (syndrome or disease)) or (myoton* adj2 (dystroph* or atrophica* or myopathy*)) or (Steinert* adj disease*) or Ricker Syndrome* or PROMM* or (Hereditary adj3 Spastic Paraplegia*) or Spastic Paraplegia Hypertrophic	400157

	Motor Sensory Neuropathy or "CMT with Pyramidal Features" or (Spastic Paraplegia adj2 ("2" or II)) or SPG2 or (rare adj3 hereditary ataxia*) or ((DiGeorge or Di George) adj (syndrome* or sequence or anomaly)) or ((velocardiofacial or Velo Cardio Facial or 22q11 or vcf or pharyngeal pouch or Thymic Aplasia or Sedlackova or Shprintzen) adj2 syndrome*) or "Autosomal Dominant Opitz G Bbb Syndrome" or "Conotruncal Anomaly Face Syndrome" or Catch22 or neurofibromatos* or recklinghausen* or (multiple adj1 neurofibroma*) or ((Turner* or Ullrich* or XO or 45X) adj3 (syndrome* or disease* or state or status or stigma*)) or (Gonadal Dysgenesis adj XO) or Monosomy X or (Bonnevie Ullrich adj (syndrome* or status)) or (mitochondrial adj (disease* or disorder*)) or respiratory chain deficienc* or oxidative phosphorylation deficienc* or (Noonan* adj3 (syndrome* or disease*)) or ((Klinefelter* or XXY or XXYY or XXXXY) adj3 (syndrome* or trisom* or disease*))).tw,kw.	
3	1 or 2	567405
4	fatigue/ or exhaustion/ or lassitude/ or muscle fatigue/	223016
5	(fatigue or lassitude or tiredness or vitality or exhaust*).tw,kw.	252449
6	4 or 5	353655
7	3 and 6	8241

APA PsycINFO 1806 to March Week 4 2020 (Ovid)

1	Muscular Dystrophy/ or Porphyria/ or Hemophilia/ or Cystic Fibrosis/ or Charcot-Marie-Tooth Disease/ or exp Neurofibromatosis/ or Turners Syndrome/ or Klinefelters Syndrome/	19222
2	((rare adj3 (disease* or disorder*)) or (orphan adj (disease* or disorder*)) or (osteogenesis adj imperfecta) or brittle bone disease* or fragilitas ossium or osteopsathyrosis or ((lobstein* or bruck*) adj (disease* or syndrome*)) or ((skeletal or fibrous) adj3 (dysplasia* or bone*)) or achondroplas* or Diaphyseal Aclasis or ((Hereditary or Familial or Multiple) adj2 (Exostos* or Chondrodysplas* or Osteochondroma*)) or Bessel-Hagen Disease or (Fibro* adj Dysplasia*) or Jaffe Lichtenstein* or (Marfan* adj2 (syndrome* or disease* or disorder* or abiotrophy)) or (Ehlers adj Danlos) or (Loeys adj Dietz) or (genetic adj3 aortic) or ((musc* or limb-girdle) adj dystroph*) or (Glycogen Storage Disease Type adj (II or "2")) or (glycogenos* adj2 (II or "2" or generali*)) or (Pompe adj (disease* or syndrome* or disorder*)) or porphyria* or (porphyrin adj (disorder* or disease* or syndrome*)) or hemophilia* or haemophilia* or ((cystic or pancreatic) adj2 fibros*) or mucoviscidosis or mucoviscoidosis or (pancrea* adj (fibrocystic or fibros* or cystic)) or myelomeningocele* or (neural tube adj2 defect*) or spina bifida* or (congenital adj3 (limb* or exremit*)) or ((limb* or exremit*) adj3 (deformi* or malform* or anomalit*)) or Arthrogryposis multiplex congenita or Amyoplasia* or (Charcot adj Marie) or (Roussy adj Levy) or Peroneal Muscular Atroph* or Hereditary Areflexic Dystasia* or ("hereditary motor and sensory" adj neuropat*) or (HMSN adj2 (I or II or 1A or 1B or "5" or V)) or HMSN1A or HMSN1B or ((Strumpel* or Struempel*) adj1 (disease or syndrome)) or (familial spastic adj (paralysis or paraparesis or paraplegia)) or (dejerine sottas adj (syndrome or disease)) or (myoton* adj2 (dystroph* or atrophica* or myopathy*)) or (Steinert* adj disease*)	12641

	or Ricker Syndrome* or PROMM* or (Hereditary adj3 Spastic Paraplegia*) or Spastic Paraplegia Hypertrophic Motor Sensory Neuropathy or "CMT with Pyramidal Features" or (Spastic Paraplegia adj2 ("2" or II)) or SPG2 or (rare adj3 hereditary ataxia*) or ((DiGeorge or Di George) adj (syndrome* or sequence or anomaly)) or ((velocardiofacial or Velo Cardio Facial or 22q11 or vcf or pharyngeal pouch or Thymic Aplasia or Sedlackova or Shprintzen) adj2 syndrome*) or "Autosomal Dominant Opitz G Bbb Syndrome" or "Conotruncal Anomaly Face Syndrome" or Catch22 or neurofibromatos* or recklinghausen* or (multiple adj1 neurofibroma*) or ((Turner* or Ullrich* or XO or 45X) adj3 (syndrome* or disease* or state or status or stigma*)) or (Gonadal Dysgenesis adj XO) or Monosomy X or (Bonnevie Ullrich adj (syndrome* or status)) or (mitochondrial adj (disease* or disorder*)) or respiratory chain deficienc* or oxidative phosphorylation deficienc* or (Noonan* adj3 (syndrome* or disease*)) or ((Klinefelter* or XXY or XXYY or XXXXY) adj3 (syndrome* or trisom* or disease*))).tw.	
3	1 or 2	27020
4	Fatigue/	8968
5	(fatigue or lassitude or tiredness or vitality or exhaust*).tw.	43711
6	4 or 5	43975
7	3 and 6	302

AMED (Allied and Complementary Medicine) <1985 to March 2020> (Ovid)

1	exp Muscular Dystrophy/ or Hemophilia/ or Cystic Fibrosis/ or exp Neural Tube Defects/ or Foot Deformities Congenital/ or exp "Neuropathies Hereditary Motor and Sensory"/ or "Neuropathies Hereditary Sensory and Autonomic"/	1330
2	((rare adj3 (disease* or disorder*)) or (orphan adj (disease* or disorder*)) or (osteogenesis adj imperfecta) or brittle bone disease* or fragilitas ossium or osteopsathyrosis or ((lobstein* or bruck*) adj (disease* or syndrome*)) or ((skeletal or fibrous) adj3 (dysplasia* or bone*)) or achondroplas* or Diaphyseal Aclasis or ((Hereditary or Familial or Multiple) adj2 (Exostos* or Chondrodysplas* or Osteochondroma*)) or Bessel-Hagen Disease or (Fibro* adj Dysplasia*) or Jaffe Lichtenstein* or (Marfan* adj2 (syndrome* or disease* or disorder* or abiotrophy)) or (Ehlers adj Danlos) or (Loeys adj Dietz) or (genetic adj3 aortic) or ((musc* or limb-girdle) adj dystroph*) or (Glycogen Storage Disease Type adj (II or "2")) or (glycogenos* adj2 (II or "2" or generali*)) or (Pompe adj (disease* or syndrome* or disorder*)) or porphyria* or (porphyrin adj (disorder* or disease* or syndrome*)) or hemophilia* or haemophilia* or ((cystic or pancreatic) adj2 fibros*) or mucoviscidosis or mucoviscoidosis or (pancrea* adj (fibrocystic or fibros* or cystic)) or myelomeningocele* or (neural tube adj2 defect*) or spina bifida* or (congenital adj3 (limb* or exremit*)) or ((limb* or exremit*) adj3 (deformi* or malform* or anomalit*)) or Arthrogryposis multiplex congenita or Amyoplasia* or (Charcot adj Marie) or (Roussy adj Levy) or Peroneal Muscular Atroph* or Hereditary Areflexic Dystasia* or ("hereditary motor and sensory" adj neuropat*) or (HMSN adj2 (I or II or 1A or 1B or "5" or V)) or HMSN1A or HMSN1B or ((Strumpel* or Struempel*) adj1 (disease or syndrome)) or (familial spastic adj (paralysis or paraparesis or paraplegia)) or (dejerine sottas adj (syndrome or disease)) or	2238

	(myoton* adj2 (dystroph* or atrophica* or myopathy*)) or (Steinert* adj disease*) or Ricker Syndrome* or PROMM* or (Hereditary adj3 Spastic Paraplegia*) or Spastic Paraplegia Hypertrophic Motor Sensory Neuropathy or "CMT with Pyramidal Features" or (Spastic Paraplegia adj2 ("2" or II)) or SPG2 or (rare adj3 hereditary ataxia*) or ((DiGeorge or Di George) adj (syndrome* or sequence or anomaly)) or ((velocardiofacial or Velo Cardio Facial or 22q11 or vcf or pharyngeal pouch or Thymic Aplasia or Sedlackova or Shprintzen) adj2 syndrome*) or "Autosomal Dominant Opitz G Bbb Syndrome" or "Conotruncal Anomaly Face Syndrome" or Catch22 or neurofibromatos* or recklinghausen* or (multiple adj1 neurofibroma*) or ((Turner* or Ullrich* or XO or 45X) adj3 (syndrome* or disease* or state or status or stigma*)) or (Gonadal Dysgenesis adj XO) or Monosomy X or (Bonnevie Ullrich adj (syndrome* or status)) or (mitochondrial adj (disease* or disorder*)) or respiratory chain deficienc* or oxidative phosphorylation deficienc* or (Noonan* adj3 (syndrome* or disease*)) or ((Klinefelter* or XXY or XXYY or XXXXY) adj3 (syndrome* or trisom* or disease*))).mp.	
3	1 or 2	2374
4	exp Fatigue/	3091
5	(fatigue or lassitude or tiredness or vitality or exhaust*).mp.	7964
6	4 or 5	8114
7	3 and 6	80

Cochrane Database of Systematic Reviews and Cochrane Central Register of Controlled Trials (Cochrane Library, Wiley)

#1	([mh ^"Rare Diseases"] OR [mh ^"Osteogenesis Imperfecta"] OR [mh ^"Exostoses, Multiple Hereditary"] OR [mh "Fibrous Dysplasia of Bone"] OR [mh ^Achondroplasia] OR [mh ^"Marfan Syndrome"] OR [mh ^"Ehlers-Danlos Syndrome"] OR [mh ^"Loeys-Dietz Syndrome"] OR [mh "Muscular Dystrophies"] OR [mh ^"Glycogen Storage Disease Type II"] OR [mh Porphyrias] OR [mh ^"Hemophilia A"] OR [mh ^"Hemophilia B"] OR [mh ^"Cystic Fibrosis"] OR [mh "Neural Tube Defects"] OR [mh "Limb Deformities, Congenital"] OR [mh ^"Charcot-Marie-Tooth Disease"] OR [mh ^"Spastic Paraplegia, Hereditary"] OR [mh ^"DiGeorge Syndrome"] OR [mh Neurofibromatoses] OR [mh ^"Turner Syndrome"] OR [mh "Mitochondrial Diseases"] OR [mh ^"Noonan Syndrome"] OR [mh ^"Klinefelter Syndrome"])	3646
#2	((rare NEAR/2 (disease* OR disorder*)) OR (orphan NEXT (disease* OR disorder*)) OR (osteogenesis NEXT imperfecta) OR (brittle NEXT bone NEXT disease*) OR "fragilitas ossium" OR osteopsathyrosis OR ((lobstein* OR bruck*) NEXT (disease* OR syndrome*)) OR ((skeletal OR fibrous) NEAR/2 (dysplasia* OR bone*)) OR achondroplas* OR "Diaphyseal Aclasis" OR ((Hereditary OR Familial OR Multiple) NEAR/1 (Exostos* OR Chondrodysplas* OR Osteochondroma*)) OR "Bessel-Hagen Disease" OR (Fibro* NEXT Dysplasia*) OR (Jaffe NEXT Lichtenstein*) OR (Marfan* NEAR/1 (syndrome* OR disease* OR disorder* OR abiotrophy)) OR (Ehlers NEXT Danlos) OR (Loeys NEXT Dietz) OR (genetic NEAR/2 aortic) OR ((musc* OR limb-girdle) NEXT dystroph*) OR	10665

	(("Glycogen Storage Disease Type") NEXT (II OR 2)) OR (glycogenos* NEAR/1 (II OR 2 OR generali*)) OR (Pompe NEXT (disease* OR syndrome* OR disorder*)) OR porphyria* OR (porphyrin NEXT (disorder* OR disease* OR syndrome*)) OR hemophilia* OR haemophilia* OR ((cystic OR pancreatic) NEAR/1 fibros*) OR mucoviscidosis OR mucoviscoidosis OR (pancrea* NEXT (fibrocystic OR fibros* OR cystic)) OR myelomeningocele* OR ("neural tube" NEAR/1 defect*) OR (spina NEXT bifida*) OR (congenital NEAR/2 (limb* OR exremit*)) OR ((limb* OR exremit*) NEAR/2 (deformi* OR malform* OR anomalit*)) OR "Arthrogryposis multiplex congenita" OR Amyoplasia* OR (Charcot NEXT Marie) OR (Roussy NEXT Levy) OR (Peroneal NEXT Muscular NEXT Atroph*) OR (Hereditary NEXT Areflexic NEXT Dystasia*) OR ("hereditary motor and sensory" NEXT neuropat*) OR (HMSN NEAR/1 (I OR II OR "1A" OR "1B" OR "5" OR V)) OR HMSN1A OR HMSN1B OR ((Strumpel* OR Struempel*) NEAR/1 (disease OR syndrome)) OR ("familial spastic" NEXT (paralysis OR paraparesis OR paraplegia)) OR ("dejerine sottas" NEXT (syndrome OR disease)) OR (myoton* NEAR/1 (dystroph* OR atrophica* OR myopathy*)) OR (Steinert* NEXT disease*) OR (Ricker NEXT (Syndrome* OR PROMM*)) OR (Hereditary NEAR/2 "Spastic Paraplegia*") OR "Spastic Paraplegia Hypertrophic Motor Sensory Neuropathy" OR "CMT with Pyramidal Features" OR ("Spastic Paraplegia" NEAR/1 ("2" OR II)) OR SPG2 OR (rare NEAR/2 (hereditary NEXT ataxia*)) OR ((DiGeorge OR "Di George") NEXT (syndrome* OR sequence OR anomaly)) OR ((velocardiofacial OR "Velo Cardio Facial" OR "22q11" OR vcf OR "pharyngeal pouch" OR "Thymic Aplasia" OR Sedlackova OR Shprintzen) NEAR/1 syndrome*) OR "Autosomal Dominant Opitz G Bbb Syndrome" OR "Conotruncal Anomaly Face Syndrome" OR "Catch22" OR neurofibromatos* OR recklinghausen* OR (multiple NEAR/1 neurofibroma*) OR ((Turner* OR Ullrich* OR XO OR "45X") NEAR/2 (syndrome* OR disease* OR state OR status OR stigma*)) OR ("Gonadal Dysgenesis" NEXT XO) OR "Monosomy X" OR ("Bonnevie Ullrich" NEXT (syndrome* OR status)) OR (mitochondrial NEXT (disease* OR disorder*)) OR (respiratory NEXT chain NEXT deficienc*) OR (oxidative NEXT phosphorylation NEXT deficienc*) OR (Noonan* NEAR/2 (syndrome* OR disease*)) OR ((Klinefelter* OR XXY OR XXYY OR XXXXY) NEAR/2 (syndrome* OR trisom* OR disease*))):ti,ab,kw	
#3	#1 OR #2	10999
#4	[mh ^Fatigue]	3384
#5	(fatigue OR lassitude OR tiredness OR vitality OR exhaust*):ti,ab,kw	37502
#6	#4 OR #5	37502
#7	#3 AND #6	366
#8	#7 in Cochrane Reviews, Cochrane Protocols	92
#9	#7 in Trials	274

CINAHL (EBSCO)

S1	(MH "Rare Diseases" OR MH "Osteogenesis Imperfecta" OR MH "Fibrous Dysplasia of Bone+" OR MH "Achondroplasia" OR MH "Marfan Syndrome" OR MH "Ehlers-Danlos Syndrome" OR MH "Loeys-Dietz Syndrome" OR MH "Muscular Dystrophy+" OR MH "Glycogen Storage Disease" OR MH "Porphyrias+" OR MH "Hemophilia+" OR MH "Cystic Fibrosis" OR MH "Neural Tube Defects+" OR MH "Limb Deformities, Congenital+" OR MH "Neuropathies, Hereditary Motor and Sensory+" OR MH "DiGeorge Syndrome" OR MH "Neurofibromatoses+" OR MH "Turner's Syndrome" OR MH "Mitochondrial Diseases+" OR MH "Noonan Syndrome" OR MH "Klinefelter's Syndrome")	32485
S2	((rare N1 (disease* OR disorder*)) OR (orphan N0 (disease* OR disorder*)) OR "osteogenesis imperfecta" OR "brittle bone disease*" OR "fragilitas ossium" OR osteopsathyrosis OR ((lobstein* OR bruck*) N0 (disease* OR syndrome*)) OR ((skeletal OR fibrous) N1 (dysplasia* OR bone*)) OR achondroplas* OR "Diaphyseal Aclasis" OR ((Hereditary OR Familial OR Multiple) N1 (Exostos* OR Chondrodysplas* OR Osteochondroma*)) OR "Bessel-Hagen Disease" OR ("Fibro* N0 Dysplasia*) OR "Jaffe Lichtenstein*" OR (Marfan* N1 (syndrome* OR disease* OR disorder* OR abiotrophy)) OR "Ehlers Danlos" OR "Loeys Dietz" OR (genetic N1 aortic) OR ((musc* OR limb-girdle) N0 dystroph*) OR (("Glycogen Storage Disease Type") N0 (II OR 2)) OR (glycogenos* N1 (II OR 2 OR generali*)) OR (Pompe N0 (disease* OR syndrome* OR disorder*)) OR porphyria* OR (porphyrin N0 (disorder* OR disease* OR syndrome*)) OR hemophilia* OR haemophilia* OR ((cystic OR pancreatic) N1 fibros*) OR mucoviscidosis OR mucoviscoidosis OR (pancrea* N0 (fibrocystic OR fibros* OR cystic)) OR myelomeningocele* OR ("neural tube" N1 defect*) OR "spina bifida*" OR (congenital N1 (limb* OR exremit*)) OR ((limb* OR exremit*) N1 (deformi* OR malform* OR anomalit*)) OR "Arthrogryposis multiplex congenita" OR Amyoplasia* OR "Charcot Marie" OR "Roussy Levy" OR "Peroneal Muscular Atroph*" OR "Hereditary Areflexic Dystasia*" OR ("hereditary motor and sensory" N0 neuropat*) OR (HMSN N1 (I OR II OR "1A" OR "1B" OR "5" OR V)) OR HMSN1A OR HMSN1B OR ((Strumpel* OR Struempel*) N1 (disease OR syndrome)) OR ("familial spastic" N0 (paralysis OR paraparesis OR paraplegia)) OR ("dejerine sottas" N0 (syndrome OR disease)) OR (myoton* N1 (dystroph* OR atrophica* OR myopathy*)) OR "Steinert* disease*" OR "Ricker Syndrome*" OR PROMM* OR (Hereditary N1 "Spastic Paraplegia*") OR "Spastic Paraplegia Hypertrophic Motor Sensory Neuropathy" OR "CMT with Pyramidal Features" OR ("Spastic Paraplegia" N1 ("2" OR II)) OR SPG2 OR (rare N1 "hereditary ataxia*") OR ((DiGeorge OR "Di George") N0 (syndrome* OR sequence OR anomaly)) OR ((velocardiofacial OR "Velo Cardio Facial" OR "22q11" OR vcf OR "pharyngeal pouch" OR "Thymic Aplasia" OR Sedlackova OR Shprintzen) N1 syndrome*) OR "Autosomal Dominant Opitz G Bbb Syndrome" OR "Conotruncal Anomaly Face Syndrome" OR Catch22 OR neurofibromatos* OR recklinghausen* OR (multiple N1 neurofibroma*) OR ((Turner* OR Ullrich* OR XO OR S45 X) N1 (syndrome* OR disease* OR state OR status OR stigma*)) OR ("Gonadal Dysgenesis"	14008

	N0 XO) OR "Monosomy X" OR ("Bonnevie Ullrich" N0 (syndrome* OR status)) OR (mitochondrial N0 (disease* OR disorder*)) OR "respiratory chain deficienc*" OR "oxidative phosphorylation deficienc*" OR (Noonan* N1 (syndrome* OR disease*)) OR ((Klinefelter* OR XXY OR XXYY OR XXXXY) N1 (syndrome* OR trisom* OR disease*)))	
S3	S1 OR S2	43174
S4	(MH "Fatigue" OR MH "Mental Fatigue")	19310
S5	(fatigue OR lassitude OR tiredness OR vitality OR exhaust*)	63151
S6	S4 OR S5	63151
S7	S3 AND S6	501

SveMed+

1	(noexp:"Rare Diseases" OR noexp:"Osteogenesis Imperfecta" OR noexp:"Exostoses, Multiple Hereditary" OR exp:"Fibrous Dysplasia of Bone" OR noexp:"Achondroplasia" OR noexp:"Marfan Syndrome" OR noexp:"Ehlers-Danlos Syndrome" OR noexp:"Loeys-Dietz Syndrome" OR exp:"Muscular Dystrophies" OR noexp:"Glycogen Storage Disease Type II" OR exp:"Porphyrias" OR noexp:"Hemophilia A" OR noexp:"Hemophilia B" OR noexp:"Cystic Fibrosis" OR exp:"Neural Tube Defects" OR exp:"Limb Deformities, Congenital" OR noexp:"Charcot-Marie-Tooth Disease" OR noexp:"Spastic Paraplegia, Hereditary" OR noexp:"DiGeorge Syndrome" OR exp:"Neurofibromatoses" OR noexp:"Turner Syndrome" OR exp:"Mitochondrial Diseases" OR noexp:"Noonan Syndrome" OR noexp:"Klinefelter Syndrome")	879
2	noexp:"Fatigue"	246
3	#1 AND #2	4

Scopus (Elsevier)

TITLE-ABS-KEY((((rare W/2 (disease* OR disorder*)) OR (orphan W/0 (disease* OR disorder*)) OR ("osteogenesis imperfecta") OR "brittle bone disease*" OR "fragilitas ossium" OR osteopsathyrosis OR ((lobstein* OR bruck*) W/0 (disease* OR syndrome*)) OR ((skeletal OR fibrous) W/2 (dysplasia* OR bone*)) OR achondroplas* OR "Diaphyseal Aclasis" OR ((Hereditary OR Familial OR Multiple) W/1 (Exostos* OR Chondrodysplas* OR Osteochondroma*)) OR "Bessel-Hagen Disease" OR (Fibro* W/0 Dysplasia*) OR "Jaffe Lichtenstein*" OR (Marfan* W/1 (syndrome* OR disease* OR disorder* OR abiotrophy)) OR "Ehlers Danlos" OR "Loeys Dietz" OR (genetic W/2 aortic) OR ((musc* OR limb-girdle) W/0 dystroph*) OR ("Glycogen Storage Disease Type" W/0 (II OR "2")) OR (glycogenos* W/1 (II OR "2" OR generali*)) OR (Pompe W/0 (disease* OR syndrome* OR disorder*)) OR porphyria* OR (porphyrin W/0 (disorder* OR disease* OR syndrome*)) OR hemophilia* OR haemophilia* OR ((cystic OR pancreatic) W/1 fibros*) OR mucoviscidosis OR mucoviscoidosis OR (pancrea* W/0 (fibrocystic OR fibros* OR cystic)) OR myelomeningocele* OR (neural tube W/1 defect*) OR "spina bifida*" OR (congenital W/2 (limb* OR exremit*)) OR ((limb* OR exremit*) W/2 (deformi* OR malform* OR anomalit*)) OR "Arthrogryposis multiplex congenita OR Amyoplasia*" OR "Charcot Marie" OR "Roussy Levy" OR "Peroneal Muscular Atroph*" OR "Hereditary Areflexic Dystasia*" OR ("hereditary motor and sensory" W/0 neuropat*) OR (HMSN W/1 (I OR II OR 1A OR 1B OR 5 OR V)) OR HMSN1A OR HMSN1B OR ((Strumpel* OR Struempel*) W/0 (disease OR syndrome)) OR (familial spastic

W/0 (paralysis OR paraparesis OR paraplegia)) OR ("dejerine sottas" W/0 (syndrome OR disease)) OR (myoton* W/1 (dystroph* OR atrophica* OR myopathy*)) OR (Steinert* W/0 disease*) OR "Ricker Syndrome*" OR PROMM* OR (Hereditary W/2 "Spastic Paraplegia*") OR "Spastic Paraplegia Hypertrophic Motor Sensory Neuropathy" OR "CMT with Pyramidal Features" OR ("Spastic Paraplegia" W/1 (2 OR II)) OR SPG2 OR (rare W/2 "hereditary ataxia*") OR ((DiGeorge OR "Di George") W/0 (syndrome* OR sequence OR anomaly)) OR ((velocardiofacial OR "Velo Cardio Facial" OR 22q11 OR vcf OR "pharyngeal pouch" OR "Thymic Aplasia" OR Sedlackova OR Shprintzen) W/1 syndrome*) OR "Autosomal Dominant Opitz G Bbb Syndrome" OR "Conotruncal Anomaly Face Syndrome" OR Catch22 OR neurofibromatos* OR recklinghausen* OR (multiple W/0 neurofibroma*) OR ((Turner* OR Ullrich* OR XO OR 45X) W/2 (syndrome* OR disease* OR state OR status OR stigma*)) OR ("Gonadal Dysgenesis" W/0 XO) OR "Monosomy X" OR ("Bonnevie Ullrich" W/0 (syndrome* OR status)) OR (mitochondrial W/0 (disease* OR disorder*)) OR "respiratory chain deficienc*" OR "oxidative phosphorylation deficienc*" OR (Noonan* W/2 (syndrome* OR disease*)) OR ((Klinefelter* OR XXY OR XXYY OR XXXXY) W/2 (syndrome* OR trisom* OR disease*))) AND (fatigue OR lassitude OR tiredness OR vitality OR exhaust*)))

Science Citation Index Expanded, Social Sciences Citation Index, Arts & Humanities Citation Index, Conference Proceedings Citation Index- Science, Conference Proceedings Citation Index Social Science & Humanities, Emerging Sources Citation Index (Web of Science)

TOPIC: ((((rare NEAR/2 (disease* OR disorder*)) OR (orphan NEAR/0 (disease* OR disorder*)) OR ("osteogenesis imperfecta") OR "brittle bone disease*" OR "fragilitas ossium" OR osteopsathyrosis OR ((lobstein* OR bruck*) NEAR/0 (disease* OR syndrome*)) OR ((skeletal OR fibrous) NEAR/2 (dysplasia* OR bone*)) OR achondroplas* OR "Diaphyseal Aclasis" OR ((Hereditary OR Familial OR Multiple) NEAR/1 (Exostos* OR Chondrodysplas* OR Osteochondroma*)) OR "Bessel-Hagen Disease" OR (Fibro* NEAR/0 Dysplasia*) OR "Jaffe Lichtenstein*" OR (Marfan* NEAR/1 (syndrome* OR disease* OR disorder* OR abiotrophy)) OR "Ehlers Danlos" OR "Loeys Dietz" OR (genetic NEAR/2 aortic) OR ((musc* OR limb-girdle) NEAR/0 dystroph*) OR ("Glycogen Storage Disease Type" NEAR/0 (II OR "2")) OR (glycogenos* NEAR/1 (II OR "2" OR generali*)) OR (Pompe NEAR/0 (disease* OR syndrome* OR disorder*)) OR porphyria* OR (porphyrin NEAR/0 (disorder* OR disease* OR syndrome*)) OR hemophilia* OR haemophilia* OR ((cystic OR pancreatic) NEAR/1 fibros*) OR mucoviscidosis OR mucoviscoidosis OR (pancrea* NEAR/0 (fibrocystic OR fibros* OR cystic)) OR myelomeningocele* OR (neural tube NEAR/1 defect*) OR "spina bifida*" OR (congenital NEAR/2 (limb* OR exremit*)) OR ((limb* OR exremit*) NEAR/2 (deformi* OR malform* OR anomalit*)) OR "Arthrogryposis multiplex congenita OR Amyoplasia*" OR "Charcot Marie" OR "Roussy Levy" OR "Peroneal Muscular Atroph*" OR "Hereditary Areflexic Dystasia*" OR ("hereditary motor and sensory" NEAR/0 neuropat*) OR (HMSN NEAR/1 (I OR II OR 1A OR 1B OR 5 OR V)) OR HMSN1A OR HMSN1B OR ((Strumpel* OR Struempel*) NEAR/0 (disease OR syndrome)) OR (familial spastic NEAR/0 (paralysis OR paraparesis OR paraplegia)) OR ("dejerine sottas" NEAR/0 (syndrome OR disease)) OR (myoton* NEAR/1 (dystroph* OR atrophica* OR myopathy*)) OR (Steinert* NEAR/0 disease*) OR "Ricker Syndrome*" OR PROMM* OR (Hereditary NEAR/2 "Spastic Paraplegia*") OR "Spastic Paraplegia Hypertrophic Motor Sensory Neuropathy" OR "CMT with Pyramidal Features" OR ("Spastic Paraplegia" NEAR/1 (2 OR II)) OR SPG2 OR (rare NEAR/2 "hereditary ataxia*") OR ((DiGeorge OR "Di George") NEAR/0 (syndrome* OR sequence OR

anomaly)) OR ((velocardiofacial OR "Velo Cardio Facial" OR 22q11 OR vcf OR "pharyngeal pouch" OR "Thymic Aplasia" OR Sedlackova OR Shprintzen) NEAR/1 syndrome*) OR "Autosomal Dominant Opitz G Bbb Syndrome" OR "Conotruncal Anomaly Face Syndrome" OR Catch22 OR neurofibromatos* OR recklinghausen* OR (multiple NEAR/0 neurofibroma*) OR ((Turner* OR Ullrich* OR XO OR 45X) NEAR/2 (syndrome* OR disease* OR state OR status OR stigma*)) OR ("Gonadal Dysgenesis" NEAR/0 XO) OR "Monosomy X" OR ("Bonnevie Ullrich" NEAR/0 (syndrome* OR status)) OR (mitochondrial NEAR/0 (disease* OR disorder*)) OR "respiratory chain deficienc*" OR "oxidative phosphorylation deficienc*" OR (Noonan* NEAR/2 (syndrome* OR disease*)) OR ((Klinefelter* OR XXY OR XXYY OR XXXXY) NEAR/2 (syndrome* OR trisom* OR disease*))) AND (fatigue OR lassitude OR tiredness OR vitality OR exhaust*)))
Indexes=SCI-EXPANDED, SSCI, A&HCI, CPCI-S, CPCI-SSH, ESCI Timespan=All years
