# Supplementary material for: Experienced fatigue in people with rare disorders: a scoping review on characteristics of existing research
Source: Orphanet J Rare Dis. 2022 Jan 10;17:14. doi: 10.1186/s13023-021-02169-6 (PMC8751355; doi:10.1186/s13023-021-02169-6)
Supplement: Supplementary file 3 — Additional file 3. Data extraction of included articles.pdf. Data is presented for each included reference. References are presented in alphanetical order in diagnsotic grups and diagnoses. [file 13023_2021_2169_MOESM3_ESM.pdf]

Additional file 3. Data extraction of included articles
Data is presented in alphabetical order in diagnostic groups and diagnoses.

**Reference**<br><br>*Author, publication year title, country of included participants*	**Aim of the study**<br><br>*As reported by the authors*	**Participants**<br><br>*Number, diagnosis, Age group (children/ adults) gender*	**Research question on fatigue, reported in the study**<br><br>*-Prevalence/ presence of experienced fatigue*<br>*-Associated factors*<br>*-Diagnostics; development or validation of assessment tools*<br>*-Intervention effects*<br>*-Views and experiences*	**Study design**<br><br>*As reported by the authors*	**Fatigue definition and focus on fatigue.**<br><br>*Was fatigue definition described in introduction?*<br><br>*Investigation of experienced fatigue primary or secondary aim/ outcome?*<br><br>*Fatigue major or medium/ minor focus? Fatigue mentioned how many times? (review authors assessment)*	**Methods used for investigating experienced fatigue**<br><br>*Quantitative studies*<br>*-Use of study-specific questionnaire?*<br>*-Use of a standardized fatigue instrument? (Which instrument?)*<br><br>*Qualitative studies*<br>*-Individual interviews*<br>*-Focus group interviews*<br>*-Other qualitative methods?*
**RARE DEVELOPMENTAL DEFECTS/ ANOMALIES DURING EMBRYOGENESIS**						
Arthrogryposis						
Dai, S., et al. 2018<br><br>Disability in adults with arthrogryposis is severe, partly invisible, and varies by genotype<br><br>France	To understand the disability of adults with arthrogryposis multiplex congenita (AMC), a rare disease spectrum characterized by at least 2 joint contractures at birth in different body areas.	43 participants with AMC, 27 female, mean age 33.2.	Prevalence of fatigue	Retrospective study	Fatigue definition not given<br><br>Investigation of experienced fatigue one secondary aim<br><br>Fatigue minor focus. Fatigue mentioned 4 times	Study-specific.<br>4-day multidisciplinary evaluation. Fatigue assessed by a structured interview of psychological problems.
Congenital limb deficiency						

Johansen, H., et al. 2018a<br><br>Chronic pain and fatigue in adults with congenital unilateral upper limb deficiency in Norway. A cross-sectional study<br><br>Norway	to: 1) Provide a further description of self-reported chronic pain, 2) Study the prevalence of severe fatigue, 3) Explore the associations between chronic pain and demographic and clinical factors and 4) Explore the associations between fatigue and demographic and clinical factors among adults with CUULD in Norway.	77 adults with congenital unilateral upper limb deficiency, 71% women, mean age 42.7 (20-82)	Prevalence and associations to fatigue	Cross-sectional survey	Fatigue definition given in introduction: Fatigue is often defined as an "overwhelming sense of tiredness, lack of energy and feeling of exhaustion, mental, physical or both" (Dittner 2004)<br><br>Investigation of experienced fatigue one primary aim<br><br>Fatigue major focus. Fatigue mentioned > 70 times	Standardized fatigue instrument: Fatigue Severity Scale (FSS)
Johansen, H., et al. 2018 b<br><br>Education and work participation among adults with congenital unilateral upper limb deficiency in Norway: A cross-sectional study<br><br>Norway	to: 1) describe the level of education and work participation among adults with CUULD in Norway, 2) explore associations between work participation and demographic factors and clinical factors; limb deficiency characteristics, comorbidities, pain, fatigue, physical and mental health.	64 adults with congenital unilateral upper limb deficiency in working age (20–67), mean age 43.4 (13.7), 70% women	Prevalence of fatigue in adults in working age<br><br>Associations to work participation	Cross sectional survey	Fatigue briefly mentioned in introduction, no definition given<br><br>Investigation of experienced fatigue one secondary aim<br><br>Fatigue medium focus. Fatigue mentioned 16 times	Standardized fatigue instrument: Fatigue Severity Scale (FSS)
Klinefelter syndrome						
Nielsen, J., et al. 1988. Follow-up of 30 Klinefelter males treated with testosterone.<br><br>Denmark	All Klinefelter males who were given testosterone treatment in our laboratory during the years l970-l982 were studied	30 adult males with Klinefelter's syndrome.	Views/ experiences<br><br>(with fatigue symptoms and how it impacted on their daily life)	Qualitative study	Fatigue not mentioned in introduction, no definition given. Investigation of experienced fatigue one secondary aim. Fatigue minor focus. fatigue mentioned 6 times.	Individual telephone In-depth, semi-structured interviews
Neurofibromatosis						

Lai , J.S., et al. 2019<br><br>Patient Reported Outcomes Measurement Information System and Quality of Life in Neurological Disorders Measurement System to Evaluate Quality of Life for Children and Adolescents with Neurofibromatosis Type 1 Associated Plexiform Neurofibroma<br><br>USA	To assess the health-related quality of life of children with neurofibromatosis type 1-related plexiform neurofibromas (pNF) using a battery of patient-reported outcome measures selected based on a conceptual framework derived from input by patients, parents, and clinicians regarding the most important pNF symptoms and concerns	140 children with NF1, Mean age was 12.5 years (SD, 2.7), 64.3% were boys	Prevalence of fatigue and associations to QoL	Cross-sectional Questionnaire study	Fatigue not mentioned in introduction, no definition given,<br><br>Investigation of experienced fatigue one secondary aim.<br><br>Fatigue minor focus, Fatigue mentioned 14 times	Standardized fatigue instrument:<br>Children completed pediatric measures from the PROMIS, including fatigue scale
Talaei-Khoei, M., et al. 2017<br><br>First use of patient reported outcomes measurement information system (PROMIS) measures in adults with neurofibromatosis<br><br>USA	to broadly describe the distribution of PROMIS scores in a sample of NF patients engaged in care at a major NF center within the US. We also aim to assess for differences by NF type and other clinical-demographic variables, and to make comparisons with general population norms.	86 patients with NF, 39 males, mean age 44.07	Prevalence of fatigue compared to general population norms	Cross sectional Questionnaire study	Fatigue briefly mentioned in introduction, no definition given. Investigation of experienced fatigue one secondary aim.<br><br>Fatigue minor focus. Fatigue mentioned 8 times	Standardized fatigue instrument:<br>PROMIS instruments for physical, emotional and social domains were selected as relevant for the NF population. Measures consisted of Anxiety, Depression, Fatigue, Pain Behaviors, Satisfaction with Social Roles and Satisfaction with Discretionary Activities , Anger and Pain Interference and Physical Function
Vassallo, G., et al. 2020<br><br>Perceived fatigue in children and young adults with neurofibromatosis type 1	To describe the prevalence and severity of perceived fatigue in a young neurofibromatosis type 1 (NF1) population.	91 children (from 71 families) returned a completed questionnaire: 75 children with NF1 and 16 unaffected siblings.	Prevalence<br><br>prevalence and severity of perceived fatigue	Cross sectional questionnaire study	Fatigue definition given in introduction: "Perceived fatigue is defined as an overwhelming sense of tiredness, lack of energy or feeling of exhaustion with the knowledge that multiple factors are often contributory (Kluger 2013, Gorman 2015)".	Standardized fatigue instrument:<br>PedsQL Multidimensional Fatigue Scale Parental and child report

UK					Investigation of experienced fatigue primary aim.<br><br>Fatigue major focus. Fatigue mentioned 70 times.	
Noonan syndrome						
Croonen, E.A., et al. 2016. Perceived motor problems in daily life: Focus group interviews with people with Noonan syndrome and their relatives<br><br>The Netherlands	The aims of this study were to provide insight into the motor performance problems that people with Noonan syndrome and/or their relatives experienced, the major consequences they suffered, the benefits of interventions they experienced, and the experiences with healthcare professionals they mentioned.	10 adults with Noonan syndrome (2 joined by parent), + 23 mothers, 9 fathers and 1 cousin – reporting on 23 children with Noonan syndrome	Views and experiences<br><br>Experiences with manifestations and consequences of motor performance problems – of which fatigue was described as one them	Qualitative study	Fatigue not mentioned in introduction, no definition given<br><br>Investigation of experienced fatigue one secondary aim<br><br>Fatigue medium focus. Fatigue mentioned 15 times.	Individual interviews with open-ended questions
Silver-Russell syndrome						
Ballard, L.M., et al. 2018<br><br>Lived experience of Silver-Russell syndrome: implications for management during childhood and into adulthood<br><br>United Kingdom	to explore the lived experience of people with SRS across the lifespan.	a sample of 15 adults (six women) with genetically confirmed SRS from the UK.	Views/ experiences (Fatigue mentioned as a disabling factor especially by women over 30 years)	Qualitative study	Fatigue not mentioned in introduction, no definition given<br><br>Investigation of experienced fatigue one secondary aim<br><br>Fatigue minor focus. Fatigue mentioned 4 times	One-on-one telephone interviews with a semi-structured guide
Spina Bifida / hydrocephalus						

Murray, C. B. et al 2018. A Multimethod, Case-Controlled Study of Sleep-Wake Disturbances in Adolescents With Spina Bifida.<br><br>USA.	To compare sleep-wake disturbances in adolescents with spina bifida (SB) to typically developing (TD) peers. Exploratory analyses examined sex as moderator of disrupted sleep.	37 adolescents, 16 male, mean age 1.1 (12–18 years) with SB and a matched comparison group of 37 typically developing adolescents.	Prevalence<br><br>(females with SB reported the highest levels of sleep/wake fatigue compared with all other subgroups)	Cross sectional Observational study with sleep questionnaires and 10 days of actigraphy monitoring	Fatigue briefly mentioned in introduction<br><br>Investigation of experienced fatigue one secondary aim<br><br>Fatigue moderate focus. Fatigue mentioned 26 times	Standardized fatigue instrument: adolescent self-report with the PedsQL Multidimensional Fatigue Scale – sleep rest fatigue subscale
Sumpter, R., et al. 2012<br><br>Quality of life and behavioural adjustment in childhood hydrocephalus<br><br>Scotland	to describe parent and teacher reported behavioural outcomes and quality of life in childhood hydrocephalus, and to consider the implications for future service planning.	76 school-aged children with hydrocephalus (5–16 years) 49 male ,	Prevalence and associations<br><br>Childrens level of fatigue, reported by their parents, and correlations to QOL	Cross sectional-Questionnaire study	Fatigue not mentioned in introduction, definition not given.<br><br>Investigation of experienced fatigue one secondary aim<br><br>Fatigue minor focus. Fatigue mentioned 20 times	Standardized fatigue instrument: PedsQL Multidimensional Fatigue Scale, parent proxy report
Turner syndrome						
Savas, M., et al. 2019 Long-term cortisol exposure and associations with height and comorbidities in Turner syndrome<br><br>The Netherlands	To investigate whether TS is associated with increased long-term cortisol concentrations as measured in scalp hair and whether these are linked to cardiometabolic and psychological parameters	55 females with TS, mean age 31 (53% 45,X karyotype), and 110 age-matched female controls from a general population–based study.	Prevalence and associations (relationship with long-term cortisol exposure)	Cross sectional-Questionnaire study	Fatigue briefly mentioned in introduction, no definition given.<br><br>Investigation of experienced fatigue one secondary aim<br><br>Fatigue minor focus. Fatigue mentioned 10 times	Standardized fatigue instrument:<br><br>Checklist Individual Strength–20.
van den Hoven, A.T. et al 2020.<br>A value-based healthcare approach: Health-related quality of life and psychosocial functioning in women with Turner syndrome.	to investigate different aspects of health-related quality of life (HR-QoL) and psychosocial functioning in women with TS in order to establish new possible targets for therapy.	177 women with TS, mean age 33.	Prevalence and associations.	Cross-sectional questionnaire study	Fatigue not mentioned in introduction, no definition given<br><br>Investigation of experienced fatigue one secondary aim<br><br>Fatigue medium focus.<br>Fatigue mentioned 25 times	Standardized fatigue instrument:<br><br>Checklist Individual Strength (CIS-20)

Netherlands						
Velocardiofacial syndrome (DiGeorge syndrome or 22q11.2 deletion syndrome)						
Looman, W.S., et al. 2010<br><br>Quality of Life among Children with Velocardiofacial Syndrome<br><br>USA	To: (1) describe health related QoL among children with VCFS as reported by parents, as well as (2) determine whether QoL among children with VCFS differs by gender, (3) determine whether QoL scores of children with VCFS differ from QoL scores of healthy children and other children with chronic conditions, and (4) to describe a profile of strengths identified among children with VCFS.	Participants were parents (n = 45) of children with VCFS ages 2 to 18 years.	Prevalence of fatigue	Cross-sectional questionnaire study	Fatigue definition thoroughly described in introduction: “Fatigue is defined clinically as a decline in performance during sustained activity, and can be associated with performance on both motor and cognitive tasks” (Schwid et al., 2003).<br><br>Investigation of experienced fatigue one primary aim<br><br>Fatigue major focus. Fatigue mentioned 53 times	Standardized fatigue instrument: Parent proxy report PedsQL™ Multidimensional Fatigue Scale
Vergaelen, E. et al.2017<br>High prevalence of fatigue in adults with a 22q11.2 deletion syndrome.<br><br>Belgium	to address the following three study questions: (i) What is the level of fatigue in (young) adults with a 22q11.2 DS compared to population norms? (ii) Is there an association between the level of fatigue and prevalent somatic and psychiatric disorders in 22q11.2 DS? (iii) Is there a relation between the level of fatigue and quality of life in 22q11.2 DS?	34 adults with a 22q11.2 microdeletion, 18 female, (18-38 years)	Prevalence and associations with somatic and psychiatric disorders and relationship with quality of life	Cross-sectional questionnaire study	Fatigue discussed in introduction, no definition given.<br><br>Investigation of experienced fatigue primary aim<br><br>Fatigue major focus Fatigue mentioned 108 times.	Standardized fatigue instrument: Multidimensional fatigue inventory(MFI-20) and Beck Depression Inventory (BDI) as part of a broader psychiatric assessment

**RARE GENETIC DISEASES**						
***Other rare genetic diseases**: Articles including several groups of genetic diseases or genetic diseases not fitting into any of the other diagnostic groups*						
Bogart, K.R. & Irvin, V.L. 2017.<br><br>Health-related quality of life among adults with diverse rare disorders<br><br>USA	to quantitatively describe HRQL among adults living in the U.S. with a variety of RDs and RD categories using PROMIS, and to compare their scores to those of the general population and people with common chronic health conditions.	1218 participants with rare disorders, including: spinocerebellar ataxia (n =150), Ehlers-Danlos syndrome (n=75) , rare developmental defects (n= 71), rare bone diseases (n= 19).<br>77% female, mean age 51.50 years.	Prevalence of fatigue	Cross-sectional Online survey	Fatigue mentioned once in introduction, no definition given.<br><br>Investigation of experienced fatigue one secondary aim.<br><br>Fatigue minor focus. Fatigue mentioned 16 times	Standardized fatigue instrument:<br><br>PROMIS short form fatigue (4 questions)
Bogart, K.R & Dermody, S.S. 2020.<br><br>Relationship of rare disorder latent clusters to anxiety and depression symptoms.<br><br>USA	to identify meaningful clusters that unify many of these “orphan diseases” based on RD characteristics, and to distinguish clusters that are at risk for anxiety and depression.	1218 participants with rare disorders: including: spinocerebellar ataxia (n =150), Ehlers-Danlos syndrome (n=75) Participants were 77% female, mean age 51.50 years	Prevalence and associations to anxiety and depression	Online survey, cross sectional study	Fatigue mentioned once in introduction, no definition given<br><br>Investigation of experienced fatigue one secondary aim.<br><br>Fatigue minor focus. Fatigue mentioned 11 times	Standardized fatigue instrument:<br><br>PROMIS short form fatigue (4 questions)
Familial Mediterranean fever						
Duruoz, M.T., et al. 2018 Fatigue in familial Mediterranean fever and its relations with other clinical parameters<br><br>Turkey	to investigate fatigue in FMF patients as a disabling symptom and its associations with clinical and demographic variables.	61 patients with FMF, 44 female, mean age 34 and 61 healthy controls	Prevalence and associations<br><br>Prevalence and severity of fatigue and relations to other factors	Cross sectional questionnaire study	Fatigue definition given in introduction: “Fatigue is a common and disabling symptom in many chronic diseases. It can be defined as a state of extreme tiredness that results from mental or physical exertion or from any illness. It is different from normal tiredness in severity, quality, and unpredictability, and affects every aspect of life. Fatigue consists of physiological muscle fatigue, which can	Standardized fatigue instruments:<br>the Multidimensional Assessment of Fatigue (MAF), the Fatigue Severity Scale (FSS), the Fatigue Impact Scale (FIS), and visual analogue score of fatigue

					compromise the performance of prolonged or repeated task" (Stebbings 2010)<br><br>Investigation of experienced fatigue primary aim.<br>Fatigue major focus Fatigue mentioned >90 times	
Hereditary angio-oedema						
Magerl, M. et al. 2014 Characterization of prodromal symptoms in a large population of patients with hereditary angio-oedema<br><br>Germany	To characterize the spectrum of prodromal symptoms in patients with HAE in Germany.	365 patients with HAE, 126 male patients (median age 47 years, range 14–80) and 239 female patients (median age 44 years, range 8–83).	Prevalence of fatigue<br><br>Investigate fatigue as a prodromal symptom to angio-odema episodes	Cross-sectional questionnaire study	Fatigue not mentioned in introduction, no definition given.<br><br>Investigation of experienced fatigue one secondary aim.<br><br>Fatigue minor focus. fatigue mentioned 10 times	Study specific questionnaire: The questions covered epidemiology, triggers, prodromes, symptoms and therapy.
Primary Immunodeficiency Disorders						
Hajjar, J., et al. 2017<br><br>Increased Incidence of Fatigue in Patients with Primary Immunodeficiency Disorders: Prevalence and Associations Within the US Immunodeficiency Network Registry<br><br>USA	to estimate the prevalence of fatigue among PID patients using data from the US Immunodeficiency Network registry and investigate these data for characteristics that may be associated with fatigue.	2366 patients with different PIDs, Most common variable immunodeficiency (987), di George syndrome (393) and several other rare syndromes. Children and adults, with data on fatigue status	Prevalence and associations to fatigue	Retrospective study, using registry data	Self-reported fatigue discussed in introduction, no definition given.<br><br>Investigation of experienced fatigue primary aim.<br><br>Fatigue major focus<br>Fatigue mentioned >100 times	Registry specific questions:<br>The USIDnet uses a patient registry form, which contained on page 6 a question about presence of fatigue as one of the constitutional symptoms.

**Rare inborn metabolism diseases**						
Rare inborn metabolism diseases, mixed populations						
Johnston, B.C., et al. 2016<br><br>Limited responsiveness related to the minimal important difference of patient-reported outcomes in rare diseases<br><br>Country n/a	To systematically review the literature to identify studies involving patients with five LSDs to document the nature and responsiveness of PROs compared to surrogates (e.g., creatinine, glomerular filtration rate, left ventricular mass,<br>forced vital capacity) in interventional studies.	62 included studies addressed patients with Fabry disease (n =34, 55%), Gaucher disease (n = 12, 19%), Pompe disease (n = 10, 16%), MPS-I (n = 4, 6%), and MPS-II (n = 3, 5%).	*-Diagnostics; validation of assessment tools*	Systematic review	Fatigue definition not given<br><br>Fatigue one secondary aim.<br><br>Fatigue minor focus. Fatigue mentioned 2 times	
Barth syndrome						
Mazar, I., et al. 2019<br><br>Understanding the life experience of Barth syndrome from the perspective of adults: a qualitative one-on-one interview study<br><br>Europe and USA	to explore<br>the experience of BTHS from the perspective of adult males at least 35 years of age with the condition via in-depth qualitative interviews.	7 males with Barth syndrome, mean age 51.3.	Views and experiences<br><br>experiences of symptoms – like fatigue, disease related impacts on daily life and coping strategies	Qualitative study	Fatigue briefly mentioned in introduction, no definition given.<br><br>Investigation of experienced fatigue one secondary aim.<br><br>Fatigue minor focus. Fatigue only mentioned 3 times	Individual telephone interviews
Fabry disease						
Guffon, N. & Fouilhoux, A. 2004<br><br>Clinical benefitt in Fabry patients given enzyme replacement therapy. A case series<br><br>The Netherlands	To further ascertain whether GL-3 clearance translates into clinical benefit, a retrospective survey using a more disease-related questionnaire was conducted to determine improvement in the disease symptoms that contribute significantly to the impairment of quality of life of Fabry patients.	17 patients with Fabry disease, 15 male. Mean age 34.7 (16-55)	Intervention effects, questionnaire before and after treatment	Retrospectve survey to patients participating in enzyme replacement studies	No fatigue definition given,<br><br>Investigation of experienced fatigue one secondary aim.<br><br>Fatigue minor focus. Fatigue mentioned 10 times.	Self-constructed questionnaire<br>The questions examined Fabry disease symptoms that may impact on quality of life, including pain in the extremities, heat intolerance, gastrointestinal symptoms and fatigue

						was scored using a visual analogue scale (VAS) with scores ranging from 1 to 10, 1 =none, 10= strong).
Ivleva, A., et al. 2018<br><br>The Influence of Patient-Reported Joint Manifestations on Quality of Life in Fabry Patients<br><br>United Kingdom	to evaluate the prevalence of joint manifestations, limitations in daily life experienced due to pain and joint problems and general quality of life reported by Fabry patients compared to age-matching control healthy subjects.	77 Fabry disease patients, 49 female, median age 54 (18-83).	Prevalence of fatigue and associations to joint manifestations	Cross-sectional questionnaire study	Fatigue definition not given, briefly mentioned in introduction<br><br>Investigation of experienced fatigue one secondary aim.<br><br>Fatigue minor focus. Fatigue mentioned 10 times	Unclear how fatigue was measured.
Ramaswami, U. et al 2006<br>Clinical manifestations of Fabry disease in children: data from the Fabry Outcome Survey.<br><br>11 European countries	To present the clinical manifestations of Fabry disease in children enrolled in FOS--the Fabry Outcome Survey--a European database of the natural history of Fabry disease and the effects of enzyme replacement therapy with agalsidase alfa (Replagal).	82 patients with Fabry Disease under 18 y of age, 40 males. Median age at baseline evaluation was 12.9 y (range 0.7_ 17.9 y),	Prevalence of fatigue	Quantitative analyses of Baseline and follow-up registry data	Fatigue not mentioned in introduction, no definition given<br><br>Investigation of experienced fatigue one secondary aim.<br><br>Fatigue minor focus. Fatigue mentioned 5 times	Study/ registry specific questions:<br>Signs and symptoms are recorded using an extensive checklist, which covers most of the clinical manifestations of Fabry disease
Schmitz, B., et al. 2016<br><br>Physical Exercise in Patients with Fabry Disease - A Pilot Study<br><br>Germany	to assess the extent of exercise intolerance in Fabry disease (FD) patients and to report individual effects of physical exercise.	14 subjects (6 female), mean age 46 (18-66), were	Intervention effects	Clinical trial,with random selection	Fatigue not mentioned in introduction, no definition given<br><br>Investigation of experienced fatigue one secondary aim.<br><br>Fatigue minor focus. Fatigue mentioned 5 times	Study specific questionnaire. including questions on Patients perception of general Improvement of fatigue
Familial chylomicronemia syndrome						
Gelrud, A., et al. 2017<br><br>The burden of familial chylomicronemia	to better understand and define the daily clinical and psychosocial	10 FCS patients, six males, median age 48 years (26-67)	Views and experiences	Qualitative study	Fatigue briefly mentioned in introduction, no definition given.<br><br>Investigation of experienced fatigue one secondary aim.	Face-to-face Panel discussions

syndrome from the patients' perspective<br><br>USA	burden of FCS from the patients and caregiver perspective.				Fatigue minor focus. Fatigue mentioned 18 times	
Gaucher disease						
Hayes R.P., et.al. 1998.<br><br>The impact of Gaucher disease and its treatment on quality of life.<br><br>USA	To obtain information about how Gaucher disease and its treatment, specifically enzyme replacement therapy, affect patients' health-related quality of life	16 participants (6 male) in an ongoing study with type 1 Gaucher disease, and symptoms of chronic fatigue, median age 36 (8-67)	Views and experiences	Individual interviews with open-ended questions	Fatigue mentioned once in introduction, fatigue definition not given.<br><br>Investigation of experienced fatigue one secondary aim.<br><br>Fatigue medium focus. Fatigue mentioned 22 times	Individual interviews with structured questions
Samuels, N., et al. 2012.<br><br>Acupuncture for symptoms of Gaucher disease.<br><br>Israel	To examine the effect of acupuncture on bone/joint pain, headache and fatigue, as well as quality of life in patients with Gaucher disease (GD), within the framework of an integrated treatment programme.	12 patients with Gaucher disease, 21 – 62 years, 6 women	Intervention effects	a series of 10—12 weekly acupuncture treatment sessions.	Fatigue mentioned in introduction, no definition given.<br><br>Investigation of experienced fatigue one primary aim.<br><br>Fatigue minor focus. Fatigue mentioned 14 times	Standardized fatigue instrument: Functional Assessment of Chronic Illness Therapy-Fatigue measure (FACIT-F)
Verderese C.L., et.al. 1993. Gaucher's disease: a pilot study of the symptomatic responses to enzyme replacement therapy. J<br><br>USA	To determine the impact of enzyme replacement therapy treatment on perceptions of well-being	12 patients with Gauchers disease type 1, age and gender not reported	Intervention effect	Clinical trial	Fatigue not mentioned in introduction, no definition given.<br><br>Investigation of experienced fatigue one secondary aim.<br><br>Fatigue minor focus. Fatigue mentioned 10 times	Study specific questionnaire: with each clinic visit patients were asked to comment on presence or absence of disease specific questions in a questionnaire. Chronic fatigue one of five symptom categories, 5 questions on fatigue.

Zion, Y.C., et al. 2016<br><br>Rethinking fatigue in Gaucher disease	To provide insight regarding key considerations for fatigue in GD.	Literature review resulted in 19 publications. Of these, 6 were identified that assessed fatigue, including 2 that used specific fatigue assessment instruments.	Give some data on all research questions	Systematic litterature review<br><br>and survey	Fatigue briefly mentioned in introduction, no definition given.<br><br>Experienced fatigue primary aim<br><br>Fatigue major focus. Fatigue mentioned >100 times	
Mevalonate kinase deficiency						
Ter Haar, N.M., et al. 2016<br><br>The Phenotype and Genotype of Mevalonate Kinase Deficiency: A Series of 114 Cases From the Eurofever Registry<br><br>Europe	to describe the clinical and genetic characteristics and the response to treatment in a large, international cohort in order to increase knowledge about this rare disease and hence facilitate diagnosis and inform discussion of treatment and prognosis with affected families.	114 MKD patients. The median age at onset was 0.5 years	Prevalence of fatigue symptoms	Registry study	Fatigue not mentioned in introduction, no definition given.<br><br>Investigation of experienced fatigue one secondary aim.<br><br>Fatigue minor focus. Fatigue mentioned 8 times	Registry specific questions:<br>Clinical symptoms, including fatigue symptoms recorded in registry
Morquio A syndrome (mucopolysaccharidosis IVa)						
Hendriksz, C.J., et al. 2014<br><br>Burden of disease in patients with Morquio A syndrome: results from an international patient-reported outcomes survey<br><br>Brazil, Colombia, Germany, Spain, Turkey and the UK	to assess the global burden of disease among patients with Morquio A (or mucopolysaccharidosis IVa), including the impact on mobility/ wheelchair use, HRQoL, pain and fatigue and the interaction between these factors.	27 adults and 36 children.	Prevalence of fatigue<br><br>And associations to other factors	Cross-sectional paper-based survey	Fatigue briefly descried in intro, no definition given.<br><br>Investigation of experienced fatigue one secondary aim.<br><br>Fatigue minor focus. Fatigue mentioned 14 times	Study-specific questionnaire:<br>Fatigue was assessed by questioning the patients on the number of evenings in a week they felt extremely tired.

Mucopolysaccharidosis VII						
Haller, C., et al. 2019<br><br>Individual heat map assessments demonstrate vestronidase alfa treatment response in a highly heterogeneous mucopolysaccharidosis VII study population<br><br>USA	to evaluate vestronidase alfa treatment response in a highly heterogeneous mucopolysaccharidosis VII study population	12 patients with MPS VII, 4 males, age 8-25.	Intervention effects<br><br>Treatment effect of vestronidase alfa treatment on fatigue scores	phase 3, randomized, placebo-controlled, blind-start (or single-crossover), 48-week study	Fatigue mentioned one time in introduction, no definition.<br><br>Investigation of experienced fatigue one primary aim.<br><br>Fatigue medium focus. Fatigue mentioned approx 40 times	Standardized fatigue instruments:<br>Fatigue assessments included the clinical problem evaluation (CPE) fatigue assessment and all sections of the Pediatric Quality of Life (PedsQL)Multidimensional<br>Fatigue Scale
Harmatz, P., et al. 2018<br><br>A novel Blind Start study design to investigate vestronidase alfa for mucopoly-saccharidosis VII, an ultra-rare genetic disease<br><br>USA	to investigate the effect of vestronidase alfa treatment for mucopolysaccharidosis VII	12 patients with MPS VII	Intervention effects<br><br>Treatment effect of vestronidase alfa treatment on fatigue scores	Phase 3<br>Blind Start study design with a variable placebo run-in period	Fatigue not mentioned in introduction, no definition given.<br><br>Investigation of experienced fatigue one secondary aim.<br><br>Fatigue medium focus. Fatigue mentioned 20 times	Standardized fatigue instrument:<br>(PedsQL)-Multidimensional Fatigue Scale
Wang, R.Y., et al. 2020<br><br>The long-term safety and efficacy of vestronidase alfa, rhGUS enzyme replacement therapy, in subjects with muco-polysaccharidosis VII<br><br>USA	To report the final results of the extension study, up to an additional 144 weeks after completion of the blind-start study	12 enrolled subjects, a majority were female (8 subjects), and < 18 years-old at the<br>start of the extension study<br><br>same study as Haller 2019 and Harmatz 2018	Intervention effects<br><br>Changes in fatigue as effect of enzyme replacement therapy	Phase 3,randomized, placebo-controlled, blind-start, single crossover study	Fatigue briefly mentioned in introduction, no definition given.<br><br>Investigation of experienced fatigue one secondary aim.<br><br>Fatigue minor focus fatigue mentioned 13 times	Standardized fatigue instrument: Pediatric Quality of Life (PedsQL) Multidimensional Fatigue Scale

Paroxysmal nocturnal hemoglobiniuria						
Hillmen, P. et al. 2006.<br><br>The complement inhibitor eculizumab in paroxysmal nocturnal hemoglobinuria.<br><br>United States, Canada, Europe, and Australia	To test the safety and efficacy of eculizumab, a humanized monoclonal antibody against terminal complement protein C5 that inhibits terminal complement activation, in patients with paroxysmal nocturnal hemoglobinuria (PNH).	43 PNH patients in treatment group, median age 41 (20-85), 20 males. 44 PNH patients in placebo group	Intervention effects	Double-blind, randomized, placebo-controlled, multicenter, phase 3 trial.	Fatigue not mentioned in introduction, no definition given.<br><br>Investigation of experienced fatigue one secondary aim.<br><br>Fatigue medium focus. Fatigue mentioned 23 times	Standardized fatigue instrument: Functional Assessment of Chronic Illness Therapy Fatigue instrument FACIT-F
Marti-Carvajal, A.J., et al. 2014<br><br>Eculizumab for treating patients with paroxysmal nocturnal hemoglobinuria	To assess the clinical benefits and harms of eculizumab for treating patients with paroxysmal nocturnal hemoglobinuria (PNH).	Included in the review was one multicenter (34 sites) phase III RCT involving 87 participants from US, Canada, Europe, and Australia = 16 publications (see Hillmen 2006 and Schubert 2008)	Intervention effects	Cochrane systematic review	Fatigue not mentioned in introduction, no definition given.<br><br>Investigation of experienced fatigue one secondary aim/ outcome. Fatigue minor focus. Fatigue mentioned 21 times	
Schrezenmeier, H., et al. 2014<br><br>Baseline characteristics and disease burden in patients in the International Paroxysmal Nocturnal Hemoglobinuria Registry<br><br>Europe, North America, Asia and Africa	to report on the cross-sectional analysis of demographic and clinical characteristics of patients enrolled through June 30, 2012, and describe disease-associated morbidities commonly experienced in patients with PNH.	1610 patients (857 females, mean age 42 (3-99) .	Prevalence and associations<br><br>Prevalence of fatigue as commonly reported symptom, and correlation of fatigue with QoL	Prospective Observational study<br><br>Registry data	Fatigue mentioned In introduction, no definition given.<br><br>Investigation of experienced fatigue one primary aim/ outcome.<br><br>Fatigue medium focus.<br>Fatigue mentioned 31 times.	Standardized fatigue instrument:<br>Functional Assessment of Chronic Illness Therapy (FACIT)Fatigue

Schubert, J., et al. 2008<br>Eculizumab, a terminal complement inhibitor, improves anaemia in patients with paroxysmal nocturnal haemoglobinuria.<br>United States, Canada, Europe, and Australia	to determine the effects of eculizumab treatment on anaemia parameters including endogenous PNH RBC count, haemoglobin levels and transfusion requirements.	TRIUMPH study: 43 PNH patients in treatment group, median age 41 (20-85), 20 males. 44 PNH patients in placebo group<br>SHEPERD study: 97 PNH patients, median age 41 (18-78), 48 males	Intervention effects	Analysis of data from the phase III, double-blind, placebo-controlled, TRIUMPH study, and the open-label SHEPHERD study	Fatigue mentioned in introduction, no definition given<br>Investigation of experienced fatigue one primary aim/ outcome.<br>Fatigue medium focus. Fatigue mentioned 31 times	Standardized fatigue instrument: Functional Assessment of Chronic Illness Therapy Fatigue instrument FACIT-F
Pompe disease						
Favejee, M.M., et al. 2015<br>Exercise training in adults with Pompe disease: the effects on pain, fatigue, and functioning<br>The Netherlands	To assess if a 12-week exercise intervention to improve aerobic fitness, muscle strength, and core stability also had an impact on fatigue, pain, activity, and participation in adults with Pompe disease, an inherited neuromuscular disorder.	23 patients with PD, 12 males, median age 46 (range 20 – 71)	Intervention study	Open-label trial. Patients participated in a 12-week exercise program, which included 36 sessions of standardized aerobic, resistance, and core stability exercises.	Fatigue briefly mentioned in introduction as one factor strongly affecting Pompe patients ability to carry out daily life activities and participation, no further definition given.<br>Investigation of experienced fatigue one primary aim/ outcome.<br>Fatigue major focus. Fatigue mentioned 38 times.	Standardized fatigue instrument: Fatigue severity scale. Scores ≥4 are indicative of fatigue, and scores ≥5 are indicative of severe fatigue
Gungor, D., et al. 2013<br>Enzyme replacement therapy and fatigue in adults with Pompe disease<br>Australia, Canada, Germany, The Netherlands, United States, United Kingdom, and a small number of patients from other countries	To establish whether ERT reduces fatigue in adult Pompe patients. And if potential effect of ERT on fatigue differed between subgroups of patients, and whether it was related to improvements or changes in muscle strength, pulmonary function, and/or depression.	163 patients with PD, 90 female, mean age at start of ERT 50 (24-76)	Intervention study investigating Treatment effect of ERT on fatigue scores	Multicenter observational follow-up study on the clinical course in PD	Fatigue definition discussed in introduction: “The pathophysiology of fatigue in neurological disorders is not fully understood. As well as physiological changes in the muscle or the Central Nervous System (CNS), it may involve respiratory dysfunction and/or inadequate energy expenditure or energy production. Psychological fatigue (‘weariness’) may also be involved” (de Vries 2010, Chaudhuri 2004, Mellies 2005). Investigation of experienced fatigue primary aim.	Standardized fatigue instrument:<br>Fatigue severity scale. Scores ≥4 indicate the presence of fatigue, and scores ≥5 severe fatigue

					Fatigue major focus. Fatigue mentioned 90 times.	
Hagemans, M.L., et al. 2005<br><br>Clinical manifestation and natural course of late-onset Pompe's disease in 54 Dutch patients<br><br>The Netherlands	To obtain more knowledge on clinical manifestation and natural course of this patient population	54 patients with Pompe disease, mean age 48.6	Prevalence<br><br>Prevalence and occurrence of fatigue symptom	Questionnaire study	Fatigue not mentioned in introduction, no definition given<br><br>Investigation of experienced fatigue one secondary aim.<br><br>Fatigue minor focus. Fatigue mentioned 12 times.	Study specific questionnaire developed to cover almost all aspects of the disease (diagnosis, family history, childhood, mobility, specific movements, breathing, sleeping, eating, other complaints, daily activities, job or study, modifications to the home and use of care, hospital stays and treatments).
Hagemans, M.L., et al. 2007<br><br>Fatigue: an important feature of late-onset Pompe disease<br><br>Australia, Canada, Germany, The Netherlands, United States, United Kingdom, and a small number of patients from other countries	To investigate the prevalence and severity of fatigue in adult patients with Pompe disease.	225 patients of 18 years and older. The mean age of the study population was 47 (SD 13) years and 54% were women	Prevalence and severity of fatigue	Multicenter observational follow-up study on the clinical course in PD	Fatigue definition given in introduction: "Fatigue is difficult to define, as it is often a non-specific and subjective complaint. Two suggested definitions are 'extreme and persistent tiredness, weakness or exhaustion, mental, physical or both' [Dittner 2004] and 'difficulty in initiation of or sustaining voluntary activities' [Chaudhuri 2004]"<br><br>Investigation of experienced fatigue primary aim.<br>Fatigue major focus. Fatigue mentioned 58 times	Fatigue severity scale Scores of 4 and higher indicate that patients are suffering from fatigue and scores of 5 and higher that patients are suffering from severe fatigue
Hamed, A., et al. 2019<br><br>Mobility assessment using wearable technology in patients	to: (1) evaluate the willingness to adopt wearable devices for passive and active health monitoring and (2) explore the relationship between	29 patients, 26 females, mean age 43	Prevalence and Associations<br><br>Explore relations between symptoms – fatigue and	Observational study using fitbit and questionnaires	Fatigue mentioned once in introduction, definition not given<br><br>Investigation of experienced fatigue one secondary aim.	Standardized outcomes instrument:<br>Pompe disease symptom scale. It measures severity of breathing difficulties, fatigue and tiredness, muscle

with late-onset Pompe disease<br><br>USA	patient characteristics and disease experience (symptoms and impact) with device-measured mobility.		device-measured mobility		Fatigue minor focus. Fatigue mentioned 5 times	weakness and ache, pain, and headache from the LOPD patient's perspective
Kanters, T.A., et al. 2015<br><br>A conceptual disease model for adult Pompe disease	to develop a conceptual disease model for Pompe disease in adults	379 patients with Pompe disease, 79 female, mean age 49.3 (23-72.6)	Prevalence of fatigue and associations to other factors	Prospective follow-up study. Clinical data was collected during regular standardized follow-up examinations	Fatigue briefly mentioned in introduction, no definition given<br><br>Investigation of experienced fatigue one secondary aim.<br>Fatigue medium focus. Fatigue mentioned 21 times	Standardized fatigue instrument:<br>Fatigue Severity Scale (FSS), a score of 4 or higher indicating that a patient is fatigued
Scheidegger, O., et al. 2018<br><br>36-Months follow-up assessment after cessation and resuming of enzyme replacement therapy in late onset Pompe disease: data from the Swiss Pompe Registry<br><br>Switzerland	to report the 36-months follow-up assessments after resuming ERT.	Seven patients (4 females) with genetically confirmed LOPD	Intervention study<br><br>Changes in fatigue after cessation and resuming of enzyme replacement with follow-up after 36 months	Prospective study	Fatigue mentioned once in introduction, no definition given<br><br>Investigation of experienced fatigue one secondary aim/ outcome.<br>Fatigue minor focus. Fatigue mentioned 9 times	Standardized fatigue instrument:<br>Fatigue severity scale
Schoser, B., et al. 2017<br><br>The humanistic burden of Pompe disease: are there still unmet needs? A systematic review	To characterize the humanistic burden of Pompe disease through a systematic literature review.	17 publications included, all in patients with late-onset Pompe disease (LOPD)	Prevalence of fatigue and associations to other factors	Systematic review	Fatigue not discussed in introduction.<br><br>Investigation of experienced fatigue one secondary aim/ outcome.<br><br>Fatigue medium focus. Fatigue mentioned 31 times	
Tarnopolsky, M., et al. 2016<br><br>Pompe Disease: Diagnosis and Management.	The objective of our review was to systematically evaluate the<br>quality of evidence from the literature to formulate evidence-based guidelines for	No specific numbers are given on search strategy and included/ excluded studies	Prevalence of fatigue and associations to other factors	literature review,<br>search strategy not given,	Fatigue not mentioned in introduction, no definition given<br><br>Investigation of experienced fatigue one secondary aim/ outcome.	

Evidence-Based Guidelines from a Canadian Expert Panel	the diagnosis and management of patients with Pompe disease				Fatigue minor focus.<br>Fatigue mentioned 11 times	
Van der Meijden, J.C., et al. 2015<br><br>Ten years of the international Pompe survey: patient reported outcomes as a reliable tool for studying treated and untreated children and adults with non-classic Pompe disease.<br><br>Australia, Canada, France, Germany, Netherlands, UK, USA, and other countries	To Review the results of the 10 years of follow-up made possible by the Pompe survey.	408 patients with Pompe disease, 208 men. Median age at inclusion 47 (2-81),	Prevalence<br><br>Presence and level of fatigue and changes in fatigue after intervention with ERT treatment	Prospective questionnaire study with yearly questionnaires	Fatigue not mentioned in introduction, no definition given.<br><br>Investigation of experienced fatigue one primary aim/ outcome.<br><br>Fatigue medium focus. Fatigue mentioned 21 times	Standardized fatigue instrument:<br>Fatigue Severity Scale (FSS)
Porphyrias						
Bronish, O., et al. 2019<br><br>Acute porphyrias: a German monocentric study of the biochemical, molecular genetic, and clinical data of 62 families<br><br>Germany	to analyze a large cohort of patients with particular focus upon quality of life aspects	62 adults with acute porphyrias, 52 women.	Prevalence of fatigue symptoms	Questionnaire study	Fatigue not mentioned in introduction, no definition given<br><br>Investigation of experienced fatigue one secondary aim/ outcome.<br><br>Fatigue minor focus.<br>Fatigue mentioned 6 times	Study specific instrument: Porphyria-oriented quality of life questionnaire, consisting of 9 questions: the first part focusing on symptoms and impairments in daily life.
Naik, H., et al. 2016<br><br>Experiences and concerns of patients with recurrent attacks of acute hepatic	to explore patients' disease experience and its impact on their lives.	16 participants, 15 female, 1 male,<br><br>median age was 38 years (range 19–67 years)	Views and experiences	Qualitative study	Fatigue definition not mentioned in introduction<br><br>Investigation of experienced fatigue one secondary aim/ outcome.	Focus group interviews using online video conferencing software GoToMeeting.

porphyria: A qualitative study<br><br>USA					Fatigue minor focus fatigue mentioned 4 times	
Naik, H., et al. 2020<br><br>Evaluating the Patient-Reported Outcomes Measurement Information System scales in acute intermittent porphyria<br><br>USA	to assess the PROMIS-57 in a relatively large sample of AIP patients to determine its effectiveness as a PRO measure and explore associations with clinical and biochemical features of the disease to determine if PROMIS scales capture relevant QoL issues in AIP.	259 patients with acute intermittent porphyria, 213 female, mean age 42.9 (18-78)	Validation of outcome measure	Multicenter questionnaire study	Fatigue not mentioned in introduction, no definition given<br><br>Investigation of experienced fatigue one secondary aim/ outcome. Fatigue minor focus. Fatigue mentioned 12 times	Standardized fatigue instrument: PROMIS-57 Version 1 scale which includes the following domains: Physical Function, Anxiety, Depression, Fatigue, Sleep Disturbance, Satisfaction with Social Roles, and Pain Interference
Simon, A., et al. 2018<br><br>Patient Perspective on Acute Intermittent Porphyria with Frequent Attacks: A Disease with Intermittent and Chronic Manifestations<br><br>USA	to qualitatively characterize the experience of patients with acute intermittent porphyria<br>who have frequent attacks, as well as the impact of the disease on daily living	19 porphyria patients (15 female, mean a6e 40 (24-61)	Views and experiences<br><br>Experiences, impact on daily living	Qualitative study	Fatigue not mentioned in introduction, no definition given.<br><br>Investigation of experienced fatigue one secondary aim/ outcome. Fatigue minor focus. Fatigue mentioned 8 times	One-on-one telephone interviews with a semi-structured guide
Pyruvate kinase deficiency						
Salek, M.S., et al. 2019<br><br>Appraisal of patient-reported outcome measures in analogous diseases and recommendations for use in phase II and III clinical trials of pyruvate kinase deficiency	to (1) identify appropriate PRO instruments,<br>based on analogous diseases with similar symptoms, and compare their psychometric properties, strengths, and weaknesses; (2) build a 'Physico-Psychosocial Model' for PKD;<br>(3) provide recommendations for the choice of PRO tools for use in future clinical trials.	No studies found concerning Pyruvate kinase deficiency.<br><br>16 studies of analogous diseases included	*-Diagnostics; validation of assessment tools*	Systematic review	Fatigue not mentioned in introduction, but in methods.<br><br>Investigation of experienced fatigue one secondary aim/ outcome.<br><br>Fatigue medium focus<br>fatigue mentioned 55 times	

**Rare genetic bone diseases**						
McCune-Albright syndrome						
Akintoye. S.O., et al. 2006. Pegvisomant for the treatment of gsp-mediated growth hormone excess in patients with McCune-Albright syndrome. USA	To test the efficacy of pegvisomant in a population of patients with MAS in normalizing serum IGF-I and, secondarily, its ability to decrease serum IGFBP-3, signs and symptoms of GH excess, bone pain and markers of bone metabolism in the FD associated with MAS.	5 patients with verified MAS, 3 adults 33, 37, 39 years, 2 children 13, 17 years. 3 female	Intervention effects	Double blind, placebo controlled, crossover study.	Fatigue definition not mentioned.<br><br>Investigation of experienced fatigue one secondary aim/ outcome.<br><br>Fatigue minor focus. Fatigue mentioned 9 times	Study-specific instrument: Patients kept a weekly diary to record subjective impressions of fatigue and sweating using a visual analog scale ranging from 0 (none) to 10 (worst).
Multiple osteochondroma						
Bathen, T. et al. 2019<br><br>Fatigue and pain in children and adults with multiple osteochondromas in Norway, a cross-sectional study<br><br>Norway	Our aims were to investigate prevalence of fatigue and pain in<br>Norwegian children and adults with MO. Furthermore to compare prevalence of fatigue with reported prevalence in other groups and explore some factors that may contribute to fatigue in this population.	Patients with confirmed MO diagnosis. 11 children (7 boys and 4 girls, median age 10.7 years, range 6–16 years) and 21 adults (8 men and 13 women median age 37.1, range 21–67 years).	Prevalence of fatigue<br><br>Associated factors	Cross-sectional survey	Fatigue definitions discussed in introduction: In adults- "Extreme and persistent tiredness, weakness or exhaustion – mental, physical or both" (Dittner et al., 2004). A similar definition of fatigue in children emphasise that fatigue in children is multi-dimensional and also includes emotional aspects (McCabe et al. 2009)<br><br>Investigation of experienced fatigue one primary aim/ outcome.<br><br>Fatigue major focus. Fatigue mentioned: > 100 times	Standardized fatigue instruments:<br><br>Fatigue severity scale (adults)<br><br>PEDSQL multidimensional fatigue scale (children)

Osteogenesis imperfecta						
Arponen, H. et al. 2018.<br><br>Fatigue and disturbances of sleep in patients with osteogenesis imperfecta - a cross-sectional questionnaire study.	to examine in an adult OI population the subjective experience of fatigue, affecting the patients' everyday life, and to evaluate in particular the level of pain and prevalence of selfreported sleep disturbances, in order to assess their role in excessive daytime sleepiness and fatigue.	56 persons with OI (41 women), 16- 75 years.	Prevalence and associated factors	Cross –sectional survey	Fatigue briefly mentioned in introduction. Definition of fatigue not given<br><br>Investigation of experienced fatigue one primary aim/ outcome.<br><br>Fatigue major focus. Fatigue mentioned > 60 times	Study-specific questionnaire:<br><br>Self-reported Fatigue measured with VAS scale 0 (no fatigue) -10 (worst possible fatigue)
Harsevoort, a.G.J., et al. 2020<br><br>Fatigue in adults with Osteogenesis Imperfecta<br><br>The Netherlands	to investigate the impact of fatigue on<br>daily functioning in adults with OI compared to control populations.	99 persons with verified OI, mean age 45 (19-80)	Prevalence and associated factors	Cross-sectional cohort study; questionnaire via email/ mobile app or post.	Fatigue briefly mentioned in introduction, no definition.<br><br>Investigation of experienced fatigue primary aim/ outcome.<br><br>Fatigue major focus. Fatigue mentioned > 70 times	Standardized fatigue instrument: Fatigue severity scale
Tolboom, N., et al. 2004<br><br>Osteogenesis imperfecta in childhood: effects of spondylodesis on functional ability, ambulation and perceived competence<br><br>The Netherlands	to evaluate the effects of spondylodesis on spinal curvature, functional outcome, level of ambulation and perceived competence<br>in children with OI.	11 children with OI, 5 boys, mean age at surgical intervention 13.1.	Prevalence and associated factors	Questionnaire study, before surgery and at follow up after surgery	Fatigue not mentioned in introduction, no definition given.<br><br>Investigation of experienced fatigue one secondary aim/ outcome.<br><br>Fatigue minor focus. Fatigue mentioned 10 times	Study-specific questionnaire:<br>Scoring the amount of fatigue at last examination, on a 10-cm visual analog scale on indicating current levels of fatigue. 0=no complaints, 10 = very severe complaints.
Tosi L.L., et al. 2015<br><br>Initial report of the osteogenesis imperfecta adult	to (1) define health care concerns and perceptions of adults with OI; (2) identify health-care related issues that may have been previously missed or under-valued by adults with OI and their	952 adults with OI, 71 % female, mean age 45.1.	Prevalence	Web-based survey	Fatigue not mentioned in introduction, fatigue definition not given.<br><br>Investigation of experienced fatigue one secondary aim/ outcome.	Standardized outcomes instrument:<br><br>PROMIS, including: general physical health, pain, fatigue, physical function, sleep,

natural history initiative<br><br>North America	medical providers; and (3) compare QoL responses by adults with OI with those of benchmark populations without OI.				Fatigue minor focus. Fatigue mentioned 3 times.	depression, anxiety, sexual satisfaction, and social function
Van Brussel, M., et al. 2008<br><br>Physical training in children with osteogenesis imperfecta<br><br>The Netherlands	To study the effects of a physical training program on exercise capacity, muscle force, and subjective fatigue levels<br>in patients with mild to moderate forms of osteogenesis imperfecta (OI).	Thirty-four children with OI type I or IV Intervention group: 16, 6 boys, mean age 12.3 (7.9-17.8). Control group: 17, 5 boys, mean age 13.2 (8.3-18.6)	Intervention effects	Treatment study with randomly assignment to either a 12-week graded exercise program<br>or care as usual for 3 months	Fatigue briefly mentioned in introduction, no definition given.<br><br>Investigation of experienced fatigue one secondary aim/ outcome.<br><br>Fatigue medium focus.<br>Fatigue mentioned 23 times	Standardized fatigue instrument:<br>the subscale subjective fatigue of the self-report questionnaire Checklist Individual Strength-20 (CIS-20).
X-Linked Hypophoshataemia						
Ferizovic, N., et al. 2020<br><br>Exploring the Burden of X-Linked Hypophosphataemia: An Opportunistic Qualitative Study of Patient Statements Generated During a Technology Appraisal<br><br>UK, USA, Australia	To qualitatively determine the real-world symptom<br>and treatment burden of XLH on children, adolescents and adults.	89 Statements from 26 Parents to 32 children with XLH, 13 adolescents with XLH and 33 adults with	Views/ experiences<br><br>(Fatigue as one experienced symptom)	Qualitative study	Fatigue briefly mentioned in introduction as one factor affecting XLH patients<br><br>Investigation of experienced fatigue one secondary aim/ outcome.<br><br>Fatigue minor focus. Fatigue mentioned 6 times	Retrospective qualitative analysis of the burden of XLH using the statements generated during the technology appraisal of burosumab in an online public open consultation.
Lo, S.H., et al. 2020<br><br>Exploring the burden of X-linked hypophosphatemia: a European multi-country qualitative study<br><br>Finland, France, Germany, Luxembourg, UK	to provide<br>an in-depth, qualitative understanding of the nature and<br>impact of pain, stiffness and fatigue symptoms in XLH as well as the psychosocial impact of XLH as a lifelong hereditary<br>condition.	30 adults with XLH, 21 women, mean age 40 (26-69)	Views / experiences (Experience of fatigue and impact on daily life)	Qualitative study	Fatigue mentioned in introduction, no definition given.<br><br>Investigation of experienced fatigue one primary aim/ outcome.<br><br>Fatigue major focus.<br>Fatigue mentioned 55 times	Semi-structured telephone interviews

Theodore-Oklota, C., et al. 2020<br><br>Qualitative Research to Explore the Patient Experience of X-Linked Hypophosphatemia and Evaluate the Suitability of the BPI-SF and WOMAC R as Clinical Trial End Points<br><br>USA	To increase understanding of the patient experience of XLH and establish the main symptom and impact concepts of importance to patients with XLH. Through cognitive debriefing, the study also explored whether the BPI-SF and WOMAC® are relevant and appropriate instruments for measuring pain and stiffness as endpoints in clinical trials with adult patients with XLH,	18 patients with XLH, 15 female, mean age 24 (20-60)	Views / experiences	Qualitative study	Fatigue briefly mentioned in introduction, no definition given.<br><br>Investigation of experienced fatigue one secondary aim/ outcome.<br><br>Fatigue minor/ medium focus. Fatigue mentioned 9 times	Face-to-face, semi-structured interviews
Rare genetic haematological disorders						
Congenital (hereditary) Thrombotic Thrombocytopenic Purpura						
Oladapo, A.O., et al. 2019<br><br>Patient Experience with Congenital (Hereditary) Thrombotic Thrombocytopenic Purpura: A Conceptual Framework of Symptoms and Impacts<br><br>USA	to gain an in-depth understanding of patients' experiences with the congenital form of thrombotic thrombocytopenic purpura, including the most salient symptoms and impacts associated with congenital thrombotic thrombocytopenic purpura and its treatment	11 patients (8 female, mean age, 38.2 years; range 21–52 years)	Views and experiences	Qualitative study	Fatigue definition not discussed in introduction<br><br>Investigation of experienced fatigue one secondary aim/ outcome.<br><br>Fatigue minor focus. Fatigue mentioned 10 times	Concept elicitation telephone interviews
Hemophilia						
Forsyth, A.L., et al. 2014<br><br>Haemophilia Experiences, Results and Opportunities (HERO) Study: survey methodology and population demographics	to describe the multinational HERO quantitative study, with particular focus on the survey methodology and the demographic characteristics of adult PWH (hereafter referred to as PWH) and parents/caregivers (hereafter referred to as parents) of	675 patients with haemophilia, median age 36 (18-86)	Prevalence	Cross-sectional online survey	Fatigue not mentioned in introduction, no definition given<br><br>Investigation of experienced fatigue one secondary aim/ outcome.<br><br>Fatigue minor focus. Fatigue mentioned 2 times	Study- specific questionnaire with some standardized outcome measures. Constructed in cooperation with the patient organization. Asks about fatigue as a symptom and if patients related this to haemophilia or not.

US, China, Algeria, Argentina, UK, Germany, Italy, Spain, Chile	children with haemophilia who participated in the study.					
Severe aplastic anemia						
Pickard, A.S., et al. 2017<br><br>Value of transfusion independence in severe aplastic anemia from patients' perspectives - a discrete choice experiment<br><br>USA, France	to elicit patient preferences for attributes associated with severe aplastic anemia (SAA) treatment, including transfusion independence.	30 patients with SAA, 9 male. Age 18 – 65+ (mean not given).	Prevalence and associations<br><br>Prevalence of fatigue and how this impacted on the patients preference for treatment	one-time, non-interventional anonymous online survey study	Chronic fatigue mentioned once in the introduction as a common symptom in this patient group. No definition given.<br><br>Investigation of experienced fatigue one secondary aim/ outcome.<br><br>Fatigue medium focus. fatigue mentioned 28 times	Study-specific questionnaire: Including one question on fatigue: Fatigue? (none, moderate, severe)
Rare genetic neurologic diseases						
Charcot Marie Tooth disease (hereditary motor and sensory neuropathy 1A)						
Anens, E., et al.2015<br><br>Exploratory Study of Physical Activity in Persons With Charcot-Marie-Tooth Disease<br><br>Sweeden	to explore and describe the perceived facilitators and barriers to physical activity, and to use a quantitative approach to examine physical activity correlates in persons with CMT disease.	44 Adults with CMT, 54,5 % men, 45 – 65 years.	Prevalence of severe fatigue, and association to physical activity	Cross-sectional questionnaire survey with quantitative and qualitative analysis	Fatigue not mentioned in introduction, no definition given<br><br>Investigation of experienced fatigue one secondary aim/ outcome.<br><br>Fatigue medium focus. Fatigue mentioned 31 times	Standardized fatigue instrument: Fatigue Severity Scale
Boentert, M., et al. 2010.<br><br>Fatigue, reduced sleep quality and restless legs syndrome in Charcot-Marie-Tooth disease: a web-based survey.	to determine the prevalence of fatigue, daytime sleepiness, reduced sleep quality and RLS in CMT patients and their impact on health-related quality of life (HRQoL).	227 adults with CMT, 57,1% female. Age 18 – 78, mean 42.1.	Prevalence of severe fatigue, and association to HRQoL	Anonymized Web based, cross sectional survey	Fatigue described as common in CMT patients in the introduction, not defined further.<br><br>Investigation of experienced fatigue one primary aim/ outcome.<br><br>Fatigue major focus<br>Fatigue mentioned 41 times	Standardized fatigue instrument: Multidimensional Fatigue Inventory (MFI-20)

Germany						
El Mhandi L., et al. 2008<br><br>Benefits of interval-training on fatigue and functional capacities in Charcot-Marie-Tooth disease<br><br>France	to test whether 24 weeks of interval-training exercise (ITE) cycling can significantly improve physiological, neuromuscular, and functional capacities and alleviate fatigue in CMT patients.	8 adults with CMT, 4 women, mean age 34 (20-44)	Intervention effect	Intervention study with experimental Design.	Fatigue discussed in introduction, no definition given<br><br>Investigation of experienced fatigue one secondary aim/ outcome.<br><br>Fatigue medium focus. Fatigue mentioned 62 times	Study-specific fatigue measurement: Every morning throughout the training period, subjective levels of perceived soreness and tiredness for the whole body were measured with self-rated visual analog scale (VAS). (0= no fatigue. and 10= extreme fatigue)
Jagersma, E., et al. 2013<br><br>Severe Fatigue and Reduced Quality of Life in Children With Hereditary Motor and Sensory Neuropathy 1A<br><br>Netherlands	the aims of this study in children with hereditary motor and sensory neuropathy 1A are to determine the prevalence of severe fatigue, to assess the self-reported quality of life in comparison to their healthy peers, and to explore the impact of severe fatigue on quality of life.	55 children with hereditary motor and sensor neuropathy 1A, 30 girls , mean age 15.0	Prevalence of severe fatigue and associations to quality of life	cross-sectional questionnaire survey	Fatigue discussed in introduction, no definition given.<br><br>Investigation of experienced fatigue one primary aim/ outcome.<br><br>Fatigue major focus. Fatigue mentioned 101 times	Standardized fatigue instrument: Checklist Individual Strength (CIS).
Johnson, N.E., et. al. 2014<br><br>Quality-of-life in Charcot-Marie-Tooth disease: the patient's perspective<br><br>USA	to determine the prevalence and impact of symptoms that affect QOL in adult CMT.	407 patients with CMT, 63.9 female, mean age 52.3	Prevalence of fatigue symptoms	Questionnaire study	Fatigue briefly mentioned in introduction, not defined<br><br>Investigation of experienced fatigue one secondary aim/ outcome.<br><br>Fatigue minor focus.Fatigue mentioned 6 times	Study-specific questionnaire: With 20 themes representing 214 symptoms. Participants rated each symptom on a 6-point Likert scale. 1) I don't experience this; to 6) It affects my life severely.

Micallef J., et al. 2009.<br><br>Effect of ascorbic acid in patients with Charcot Marie-Tooth disease type 1A: a multicentre, randomised, double-blind, placebo-controlled trial.<br><br>France	to test the safety and efficacy of ascorbic acid in adult patients with CMT1A.	179 patients were treated: with placebo (n=62), 1 g ascorbic acid (n=56), or 3 g ascorbic acid (n=61). 163 patients completed the study. Mean age 45.1, 62% women.	Intervention effects	12-month, randomised, double-blind, placebo-controlled study	Fatigue not mentioned in introduction, fatigue definition not given.<br><br>Investigation of experienced fatigue one secondary aim/ outcome.<br><br>Fatigue minor focus. Fatigue mentioned 4 times.	Study-specific questionnaire with visual analogue scale to assess fatigue
Pareyson, D. et al 2011.<br>Ascorbic acid in charcot-marie-tooth disease type 1A (CMTTRIAAL and CMT-TRAUK): a double-blind randomised trial.<br><br>Italy and England	to test the efficacy and tolerability of ascorbic acid in patients with CMT1A	Patients with clinical and genetic diagnosis of CMT1A Treatment group: 138 patients, 78 women, mean age 43.8, placebo group: 133 patients, 85 women, mean age 41.2	Intervention effects	Double-blind randomised trial	Fatigue not mentioned in introduction, no definition given<br><br>Investigation of experienced fatigue one secondary aim/ outcome.<br><br>Fatigue minor focus<br>Fatigue mentioned 8 times	Study-specific questionnaire, including fatigue visual analogue scale at baseline and at 12 and 24 months
Ramdharry, G.M., et al. 2012<br><br>Comparing gait performance of people with Charcot-Marie-Tooth disease who do and do not wear ankle foot orthoses<br><br>UK	To see if there was a difference in the characteristics regularly use or do not regularly use AFOs. Secondary aims were to ascertain relationships between<br>gait function and clinical presentation in<br>the two groups.	32 CMT patients, 11 AFO users (7 female, mean age 43.8) and 21 non AFO users (13 male, mean age 43.4)	Prevalence of fatigue and association - relationship to walking ability and use or non-use of AFOs	Observational, cross sectional study, using questionnaires	Fatigue not mentioned in introduction, no definition given<br><br>Investigation of experienced fatigue one secondary aim/ outcome.<br><br>Fatigue minor focus.<br>Fatigue mentioned 6 times	Standardized fatigue instrument: Fatigue Severity Scale (FSS).
Ramdharry, G. M. et al. 2012<br>Exploring the experience of fatigue in people with Charcot-Marie-Tooth disease.	This qualitative study explored the phenomenon of fatigue for people with Charcot-Marie-Tooth disease, while acknowledging the triggers, impact and strategies	25 adults with CMT, 8 male, age information not given.	Views and Experiences	Qualitative	Fatigue discussed in introduction. No theoretical description of fatigue<br><br>Described through the participants perspectives – as a phenomenological approach	Focus group interviews

UK	people have developed to manage this symptom in daily life.				Investigation of experienced fatigue one primary aim.<br><br>Fatigue major focus. Fatigue mentioned 129 times	
Reynaud, V. et al. 2020 Multidimensional evaluation is necessary to assess hand function in patients with Charcot-Marie-Tooth disease type 1A<br><br>France	to evaluate the relation between severity of electroneuro-myography impairment and its impact on function and HRQoL in adults with CMT1A.	33 participants with CMT1A (23 females, mean age 47.0 years	Prevalence of fatigue and associations (relationship to electroneuromyogr aphy impairment)	cross-sectional, descriptive, observational, exploratory, prospective, mono-centric study	Fatigue not mentioned in introduction.<br><br>Investigation of experienced fatigue one secondary aim/ outcome.<br><br>Fatigue minor focus<br>Fatigue mentioned 9 times	Standardized fatigue instrument:<br>Fatigue severity scale<br>Patients with a mean score ≥4 were defined as having substantial fatigue
van der Linden, M. H. et al 2007. Ambulatory disabilities and the use of walking aids in patients with hereditary motor and sensory neuropathy type I (HMSN I).<br><br>The Netherlands	To determine the level of ambulatory disability and the use of walking aids in well-ambulant Hereditary Motor and Sensory Neuropathy type I (HMSN I) patients, and to identify the related demographic, physical and psychological variables.	Seventy-five well-ambulant HMSN I patients, aged 20 – 58 years	Prevalence and associations. (Relationship to level of ambulatory disability)	Quantitative cross-sectional	Fatigue not mentioned in introduction. No definition given. In methods section fatigue instrument characterized as measuring perceived fatigue<br><br>Investigation of experienced fatigue one secondary aim/ outcome.<br><br>Fatigue medium focus<br>Fatigue mentioned 32 times	Standardized fatigue instrument: Checklist Individual Strength
White, C.M. et al. 2014<br><br>Interventions for fatigue in peripheral neuropathy	To assess the effects of drugs and physical, psychological or behavioural interventions for fatigue in adults or children with peripheral neuropathy.	The review includes three trials involving 530 people with peripheral neuropathy, one trial with 80 people with Guillain-Barré syndrome (GBS)and two trials with a total of 450 adults with Charcot-Marie-Tooth disease type 1A	Intervention effects	Cochrane Systematic review	Fatigue defined in introduction. The experience of fatigue (or subjective fatigue) has been described as a "an overwhelming sense of tiredness, lack of energy and feeling of exhaustion" that is "not relieved by rest" and is a common sequel of chronic conditions (Bleijenberg 2003; Karlsen 1999; Krupp 2003).<br><br>Investigation of experienced fatigue primary aim/ outcome.<br><br>Fatigue major focus.<br>Fatigue mentioned >100 times	

Duchenne Muscular dystrophy						
Alemdaroglu, I. et al. 2012. Acute effects of different exercises on hemodynamic responses and fatigue in Duchenne muscular dystrophy<br><br>Turkey	to investigate the acute effects of different types of exercises on hemodynamic responses and fatigue in Duchenne muscular dystrophy (DMD).	30 boys with DMD, 6-11 years (mean age 7.87)	Intervention effects<br><br>Effect of exercise on daily activity performance	Telephone interview survey next day after exercise	Fatigue discussed in introduction, no definition given.<br><br>Investigation of experienced fatigue one primary aim/ outcome.<br><br>Fatigue medium focus. Fatigue mentioned 41 times	Study-specific fatigue instrument: The Pictorial Variant of the Children's Effort Rating Table scale which was developed to evaluate fatigue levels of children subjectively with the pictures showing the severity of the fatigue due to effort. The children were asked to rate their fatigue status between 0 – 10.
Alemdaroğlu-Gürbüz, I., et a.l 2019 Reliability and validity of the turkish translation of pedsql multidimensional Fatigue scale in Duchenne Muscular Dystrophy	To perform the Turkish translation, reliability, and validity study of the PedsQLTM-3.0 Multidimensional Fatigue Scale (PedsQL-MFS) in patients with Duchenne Muscular Dystrophy (DMD).	Seventy-one boys with DMD aged 5-12 years, and 69 mothers and 2 fathers whose mean age was 37.05.	Diagnostics/ development or validation of assessment tools<br><br>Translation and validation of assessment tool for fatigue	Prospective, cross-sectional, observational study	Fatigue discussed in introduction. No definition given on experienced fatigue<br><br>Investigation of experienced fatigue primary aim/ outcome.<br><br>Fatigue major focus<br>Fatigue mentioned 65 times	Standardized fatigue instrument: PedsQL Multidimensional Fatigue Scale.
Crescimanno, G., et.al. 2019.<br><br>Quality of life in long term ventilated adult patients with Duchenne muscular dystrophy.<br><br>Italy	to evaluate quality of life (QoL) and its possible determinants in patients affected by Duchenne muscular dystrophy (DMD) in late stages of their disease, when non-invasive ventilation (NIV) is already established	48 DMD patients, mean age 29.1.	Prevalence of fatigue and associations to quality of life	Questionnaire study	Fatigue mentioned once in introduction, fatigue definition not given.<br><br>Investigation of experienced fatigue one secondary aim/ outcome.<br><br>Fatigue minor focus. Fatigue mentioned 4 times.	Standardized disease-specific instrument:<br><br>INQoL, including one part on fatigue.
Crescimanno, G., et al. 2020<br><br>Subjective sleep quality in adult patients affected by Duchenne muscular	To explore how other variables, closely associated with self-reported health complaints, contributed to subjective sleep quality in adult patients with Duchenne muscular	48 patients with DMD, mean age 29.1, range 19-44	Prevalence and associations<br><br>(Relationship to subjective sleep quality)	Cross- sectional questionnaire study	Fatigue definition not described in introduction<br><br>Investigation of experienced fatigue one secondary aim/ outcome.	Standardized fatigue instrument:<br>Fatigue severity scale.<br>A value ≥ 4 is used to identify significant fatigue.

dystrophy. Beyond nocturnal hypoventilation<br><br>Italy	dystrophy (DMD).				Fatigue medium focus. Fatigue mentioned 17 times	
El-Aloul, B., et al. 2020<br><br>Fatigue in young people with Duchenne muscular dystrophy<br><br>Canada	To describe fatigue in DMD from patients' and parents' perspectives, and to explore associations of patient characteristics with child/adolescent self-reported and parent proxy-reported fatigue.	Seventy-one boys with DMD; median age 12y, (range 5–17y, and their parents completed questionnaires	Prevalence and associations (risk factors)	Multi-center cross-sectional questionnaire study	Fatigue definition thoroughly discussed in introduction: Subjective fatigue, the focus of this study, is defined as a lack of energy or the existence of weakness or exhaustion, mentally, physically, or both. (De Groot et al 2011).<br><br>Investigation of experienced fatigue primary aim.<br><br>Fatigue major focus.<br>Fatigue mentioned >100 times	Standardized fatigue instrument: Pediatric Quality of Life Inventory (PedsQL) Multi-dimensional Fatigue Scale (MFS), by child self-report and parent proxy-report.
Messina, S., et al. 2016<br><br>Health-related quality of life and functional changes in DMD: A 12-month longitudinal cohort study<br><br>Italy	To evaluate the relationship between 12 month changes on the Generic Core Scales (GCS), the Multidimensional Fatigue Scale and the Neuromuscular Module of the PedsQLTM with several outcome measures (6 minute walk test, North Star Ambulatory Assessment and timed items) in ambulatory DMD	Ninety-eight DMD patients (mean age = 8.4 years) had both baseline and follow-up data and were included in the analysis. Forty-three patients were aged 7 years or below.	Prevalence of fatigue and associations	prospective longitudinal multi-centric cohort study	Fatigue briefly mentioned in introduction, no definition given.<br><br>Investigation of experienced fatigue one primary aim/ outcome.<br><br>Fatigue medium focus. Fatigue mentioned 20 times	Standardized fatigue instrument: PedsQL multidimensional fatigue scale, both child and proxy reports
Pangalila, R. F. et al 2015<br>Prevalence of fatigue, pain, and affective disorders in adults with duchenne muscular dystrophy and their associations with quality of life.<br><br>The Netherlands	To assess the prevalence of fatigue, pain, anxiety, and depression in adults with Duchenne muscular dystrophy (DMD), and to analyze their relationship with health-related quality of life.	79 DMD patients Mean age 28.2 (20-44)	Prevalence and associations to fatigue	Cross-sectional questionnaire study	Fatigue definition given in introduction: "In neuromuscular disorders, fatigue is described as an overwhelming sense of tiredness, lack of energy, and feeling of exhaustion". (Chaudhuri A 2004, Van Engelen B 2004, Angelini 2012, Zwarts M 2008).<br><br>Investigation of experienced fatigue one primary aim/ outcome. Fatigue	Standardized fatigue instrument: Fatigue Severity Scale.

					major focus. Fatigue mentioned 57 times.	
Wei, Y., et al.2016<br><br>Factors Associated With Health-Related Quality of Life in Children With Duchenne Muscular Dystrophy<br><br>Canada	To investigate clinical and family characteristics associated with health-related quality of life in children with Duchenne<br>muscular dystrophy.	98 participating boys with Duchenne. Mean age of 10.7.	Prevalence of fatigue and association to quality of life	Cross-sectional Questionnaire study	Fatigue not mentioned in introduction, no definition given.<br><br>Investigation of experienced fatigue one secondary aim/ outcome.<br><br>Fatigue medium focus<br>Fatigue mentioned 29 times	Standardized fatigue instrument: Pediatric Quality of Life 3.0 Multidimensional Fatigue module. Both child and parent-proxy versions were used
Facioscapulohumeral muscular dystrophy						
Andersen, G., et al. 2017. High-intensity interval training in facio-scapulohumeral muscular dystrophy type 1: a randomized clinical trial<br><br>Denmark	To explore if HIT could be performed without signs of muscle damages, could effectively improve fitness, and whether the training program was feasible to perform by patients affected by FSHD1	12 patients with FSHD1 (26- 67 years) completed the study, compared to healthy controls	Intervention effects	an 8-week randomized, controlled parallel study (part 1) with an extension of 8 weeks of unsupervised HIT (part 2).	Fatigue briefly mentioned in introduction, no definition given.<br><br>Investigation of experienced fatigue one secondary aim/ outcome.<br><br>Fatigue minor focus. Fatigue mentioned 5 times	Study specific questionnaire: Including fatigue, and muscle pain recorded daily by participants on a visual analog scale (VAS 0–100 mm).
Bankole, L.C., et al. 2016<br><br>Safety and efficacy of a 6-month home based exercise program in patients with facioscapulo-humeral muscular dystrophy. A randomized controlled trial<br><br>France	Therefore, a randomized controlled trial was conducted to investigate the effects of a 24-week adapted home-based exercise training program designed to be compatible with FSHD patients' daily professional, social, and familial activities.	Nineteen FSHD patients were enrolled.<br>In total 16 patients completed, 4 women, mean age 40.	Intervention effects	A randomized controlled trial with home-based exercise intervention	Fatigue mentioned in introduction. No fatigue definition<br><br>Investigation of experienced fatigue one secondary aim/ outcome.<br><br>Fatigue medium focus. Fatigue mentioned 24 times.	Standardized fatigue instrument: Fatigue Severity Scale.

Colson, S.S., et al. 2010<br><br>Neuromuscular electrical stimulation training: a safe and effective treatment for facioscapulo-humeral muscular dystrophy patients<br><br>France	To investigate the feasibility, safety, and effectiveness of neuromuscular electrical stimulation (NMES) strength training in facioscapulo-humeral muscular dystrophy (FSHD) patients.	9 FSHD patients, 3 women, mean age 55.2.	Intervention effects	Prospective uncontrolled before-after trial	Fatigue not mentioned in introduction, no definition given,<br><br>Investigation of experienced fatigue one secondary aim/ outcome.<br><br>Fatigue minor focus. Fatigue mentioned 15 times	Study specific questionnaire, including VAS scale for fatigue, 0mm = no pain and 100mm =unbearable used for fatigue. At the end of the training program, patients graded changes in fatigue, as “worse,” “unchanged,” or “improved.”
Goselink, R.J.M., et al. 2018<br><br>Facioscapulo-humeral Dystrophy in Childhood: A Nationwide Natural History Study<br><br>Netherlands	The objectives of this study are to (1) clinically and genetically characterize FSHD in childhood and (2) estimate the prevalence of FSHD in childhood in The Netherlands.	32 patients with FSHD, 14 male, mean age 12 (4-17).	Prevalence of fatigue	A nationwide, cross-sectional study with prospective enrolment	Fatigue not mentioned in introduction, No fatigue definition given.<br><br>Investigation of experienced fatigue one secondary aim/ outcome.<br><br>Fatigue minor focus. Fatigue mentioned 16 times	Standardized fatigue instrument: the NeuroQol fatigue domain.
Hamel, J., et al 2019<br><br>Patient-Reported Symptoms in Facioscapulo-humeral Muscular Dystrophy (PRISM-FSHD)<br><br>USA	To determine the frequency and relative importance of the most meaningful symptoms in (FSHD) and to identify the demographic and clinical features that are associated with the greatest disease burden in this population.	328 patients with FSHD, 150 males, mean age 54.5 (23-86).	Prevalence of fatigue	Cross sectional questionnaire study	Fatigue not mentioned in introduction, no definition given.<br><br>Investigation of experienced fatigue one secondary aim/ outcome.<br><br>Fatigue minor focus. Fatigue mentioned 19 times	Self-constructed survey Participants used a Likert scale to rate the severity of each potential symptom and theme
Johnson, N.E., et al. 2012<br><br>Patient-identified disease burden in facioscapulo-humeral muscular dystrophy	To identify and quantify the most critical issues and symptoms to FSHD patients.	20 patients with FSHD, age and gender not given	Views and experiences	Qualitative study	Fatigue not mentioned in introduction, no definition given.<br><br>Investigation of experienced fatigue one secondary aim/ outcome.	In-depth interviews with subjects by telephone

USA					Fatigue minor focus. Fatigue mentioned 3 times in article and 10 times in supplementary table	
Koopman, F.S., et al.2017<br><br>Cognitive behavioural therapy for reducing fatigue in post-polio syndrome and in facioscapulohumeral dystrophy: A comparison<br><br>The Netherlands	To compare fatigue-related cognitions between patients with post-polio syndrome (PPS) and facioscapulo-humeral dystrophy. (FSHD)	21 patients with PPS, 12 female, mean age 60.0 (38-72), 24 patients with FSHD, 7 female, mean age 51.3 (24-76)	Associations between cognitions and fatigue	Questionnaire study, data from a previous treatment study	Fatigue discussed in introduction. No fatigue definition given.<br><br>Investigation of experienced fatigue primary aim.<br><br>Fatigue major focus. Fatigue mentioned >100 times	Standardized fatigue measurement: the subscale fatigue severity of the Checklist Individual Strength (CIS). Fatigue-related catastrophizing was measured with the Jacobsen-Fatigue Catastrophizing Scale (J-FCS).
Schipper, K., et al. 2017<br><br>Fatigue in facioscapulohumeral muscular dystrophy: a qualitative study of people's experieramdhnces<br><br>The Netherlands	to describe how fatigue affects the lives of people with facioscapulo-humeral dystrophy (FSHD), how they experience fatigue, and how they deal with it in order to attune rehabilitation care to patients' needs.	25 patients with FSHD and severe fatigue (11 women, age 24 – 77)	Views and experiences	Qualitative study	Fatigue definition given in introduction. "Fatigue is a subjective, unpleasant symptom which incorporates total body feelings ranging from tiredness to exhaustion, creating an unrelenting overall condition which interferes with individuals' ability to function to their normal capacity" (Ream E, 1996)<br><br>Investigation of experienced fatigue primary aim. Fatigue major focus. Fatigue mentioned > 100 times	Individual semi-structured interviews
Van der Kooi, E.L., et al. 2007<br><br>Effects of training and albuterol on pain and fatigue in facioscapulohumeral muscular dystrophy<br><br>The Netherlands	to explore the prevalence, magnitude and other characteristics of pain and experienced fatigue in the group of FSHD patients participating in the previously reported, randomised controlled trial, and (2) to study the effects of albuterol and strength training on self-reported pain, experienced	Sixty-five genetically confirmed, adult FSHD patients 40 female, mean age 38.	Intervention effects	Randomised controlled trial	Fatigue discussed in introduction and In methods section described that they have chosen validated measures to measure experienced fatigue.<br>No further description given in introduction.<br><br>Investigation of experienced fatigue one primary aim.<br>Fatigue major focus.<br>Fatigue mentioned >100 times.	Standardized fatigue instrument: fatigue severity subscale of the Checklist Individual Strength (CIS).<br><br>Experienced fatigue and self-reported activity levels also measured with the daily self-observation list. (DOP), a

	fatigue, functional status and psychological distress in these patients.					Daily Observed Fatigue score (DOF) and a Daily Observed Activity score (DOA) were calculated.
Voet, N., et al. 2014<br><br>Both aerobic exercise and cognitive-behavioral therapy reduce chronic fatigue in FSHD: an RCT<br><br>The Netherlands	To investigate the effect of aerobic exercise training (AET) and cognitive-behavioral therapy (CBT) on chronic fatigue in patients with facioscapulo-humeral muscular dystrophy (FSHD).	57 patients with FSHD type 1 with severe chronic fatigue were randomly allocated to AET: 28 patients CBT 25 patients, or usual care (UC) 24 patients. (ages 20 – 79)	Intervention study	multicenter, assessor-blinded, randomized clinical trial (RCT) using repeated measurement.	Fatigue discussed in introduction. No definition given.<br><br>Investigation of experienced fatigue one primary aim.<br>Fatigue major focus.<br>Fatigue mentioned 58 times	Standardized fatigue measure:<br>the fatigue subscale of the Checklist Individual Strength (CIS-fatigue). A CIS-fatigue score of 35 or more indicates severe fatigue
Hereditary ataxias						
Berntsson, S.G., et al. 2019<br><br>Inherited Ataxia and Intrathecal Baclofen for the Treatment of Spasticity and Painful Spasms<br><br>Sweeden	to evaluate the effectiveness of ITB treatment in patients with hereditary ataxia	5 adults with inherited ataxias, 3 women, age 41- 72.	Intervention effects	Observational treatment study.	Fatigue briefly mentioned in introduction, no definition given<br><br>Investigation of experienced fatigue one secondary aim/ outcome.<br>Fatigue medium focus. Fatigue mentioned 21 times	Standardized fatigue instrument: Fatigue severity scale
Bossie, H.M., et al. 2017.<br><br>Mitochondrial capacity, muscle endurance, and low energy in Friedreich ataxia<br><br>USA	To noninvasively evaluate skeletal muscle mitochondrial capacity, muscle-specific endurance, and energy/fatigue feelings in persons with Friedreich ataxia (FRDA) and able-bodied controls (AB).	16 adults with FRDA, 3 men, mean age 31.8 (10 healthy controls).	Prevalence and associations	Cross-sectional study	Both muscle-specific fatigue and perceived fatigue mentioned in introduction, definition given for the first, but not for the latter.<br><br>Investigation of experienced fatigue one primary aim/ outcome.<br>Muscle-specific fatigue main focus, experienced fatigue medium focus.<br>Fatigue mentioned 46 times	Standardized fatigue instrument:<br>The Mental and Physical Trait Energy and Fatigue Scales (TEF) used to measure 4 perceived psychological traits: mental energy; physical energy; mental fatigue; and physical fatigue.

Brusse, E., et al. 2011<br><br>Fatigue in spinocerebellar ataxia Patient self-assessment of an early and disabling symptom<br><br>The Netherlands	To identify the prevalence and severity of fatigue and predicting factors for severe fatigue in autosomal dominant spinocerebellar ataxia (SCA).	123 patients with spinocerebellar ataxia, 73 female, mean age 57.9	Prevalence and severity of fatigue, associations to other factors	Cross sectional questionnaire study	Fatigue is discussed in introduction, no definition given<br><br>Investigation of experienced fatigue primary aim.<br>Fatigue major focus. Fatigue mentioned ≥100 times.	Standardized fatigue instrument:<br>Fatigue severity scale<br>Cut off for severe fatigue ≥5
Epstein. E., et al. 2008<br><br>Health related quality of life measures in Friedreich Ataxia<br><br>USA	to examine the capacity for HRQOL measures, including the SF-36 and selected symptom-specific scales from the MSQLI, to capture self-reported disease status and activity limitation in FA.	130 patients with FA, 72 women, mean age 37	Prevalence of fatigue and associations to fatigue	Multi-center cross-sectional questionnaire study	Fatigue briefly mentioned in introduction, no definition given<br><br>Investigation of experienced fatigue one secondary aim.<br><br>Fatigue minor focus. Fatigue mentioned 8 times	Standardized fatigue instrument: Symptom-specific scales used in the MS quality of life inventory (MSQLI) were also administered to capture effects on fatigue (Modified Fatigue Impairment Scale — MFIS)
Paulsen, E.K., et al. 2010<br><br>Health-related quality of life in children with Friedreich ataxia<br><br>USA	To assess health-related quality of life scores in children with Friedreich ataxia and their parents using the PedsQL 4.0 Generic Core and Multi-dimensional Fatigue Scales.	The child version of the instruments was completed by 21 children (age 8-12 years) and their proxies; the adolescent version was completed by 22 children (age 13-18 years) and their proxies.	Diagnostics/ validation of assessment tools	Questionnaire study	Fatigue not discussed in introduction, no definition given<br><br>Investigation of experienced fatigue one secondary aim.<br><br>Fatigue medium focus.<br>fatigue mentioned 21 times	Standardized fatigue instrument:<br>PedsQL Multi-dimensional Fatigue Scale. Child self report and parent proxy
Xiong, E., et al. 2020<br><br>Health related quality of life in Friedreich Ataxia in a large heterogeneous cohort<br><br>USA	To assess the Health Related Quality of Life (HRQOL) of individuals with Friedreich Ataxia (FRDA) through responses to HRQOL questionnaires and analyzed the results in the context of disease severity, duration, and other parameters. We have also examined the	805 subjects participated in symptom specific scales testing. (mean age = 32.4), 408 female	Prevalence and associations<br><br>Longitudinal prevalence of fatigue and relation to QOL	Longitudinal Questionnaire study	Fatigue mentioned in introduction and methods, no definition given.<br><br>Investigation of experienced fatigue one secondary aim.<br><br>Fatigue medium focus. Fatigue mentioned 18 times	Standardized fatigue measure Modified Fatigue Impairment Scale (MFIS)

	manner in which such responses change over time in FRDA.					
Hereditary spastic paraplegia						
Kerstens, H., et al. 2019<br><br>Stumbling, struggling, and shame due to spasticity: a qualitative study of adult persons with hereditary spastic paraplegia<br><br>The Netherlands	to study the consequences experienced by and treatment needs in persons with 'relatively isolated' lower limb spasticity – due to hereditary spastic paraplegia (HSP)	14 Adults with HSP, age 26-72, 50% women	Views and experiences<br><br>Experiences with consequences of disease. Fatigue mentioned as a frequent symptom	Qualitative study	Fatigue not mentioned in introduction, no definition given<br><br>Investigation of experienced fatigue one secondary aim.<br>Fatigue medium focus. Fatigue mentioned 20 times	Semi-structured individual interviews
Rattay, T.W., et al. 2020<br><br>Non-motor symptoms are relevant and possibly treatable in hereditary spastic paraplegia type 4 (SPG4)<br><br>Germany	To evaluate non-motor symptoms and their relevance with regard to health-related quality of life	118 SPG4 patients, 59 men, mean age 50.4 (20-64)	Prevalence of fatigue and association to HRQoL	Questionnaire study	Fatigue briefly mentioned in introduction, fatigue definition not described.<br><br>Investigation of experienced fatigue one primary aim.<br>Fatigue medium focus. Fatigue mentioned 22 times	Standardized fatigue instrument:<br>Modified Fatigue Impact Scale (MFI)
Servelhere, K.R., et al. 2016<br><br>Non-motor symptoms in patients with hereditary spastic paraplegia caused by SPG4 mutations<br><br>Brazil	to investigate the frequency and severity of pain, fatigue, depression and daytime sleepiness in a representative cohort of patients with SPG4-HSP	Thirty adult patients with molecular confirmation of SPG4-HSP (17 male, mean age 48.3) and 30 age- and sex-matched healthy controls	Prevalence of fatigue, compared to healthy controls	cross sectional questionnaire study	Fatigue briefly mentioned in introduction, No definition given<br><br>Investigation of experienced fatigue one primary aim.<br><br>Fatigue medium focus<br>Fatigue mentioned 22 times	Standardized fatigue instrument:<br>Modified Fatigue Impact Scale (MFI)
Servelhere, K.R., et al. 2018<br><br>Botulinum toxin for hereditary spastic	To assess the efficacy of Btx-A on motor and non-motor manifestations in HSP patients.	Thirty-three adult patients with a clinical and	Intervention effects	Evaluation before and after Btx-A injections.	Fatigue briefly mentioned in introduction, no definition given.	Standardized fatigue instrument: Modified Fatigue Impact Scale (MFI)

paraplegia: effects on motor and non-motor manifestations<br><br>Brazil		molecular diagnosis of HSP. Mean age was 41.7 years and there were 18 women			Investigation of experienced fatigue one primary aim/ outcome.<br><br>Fatigue medium focus<br>Fatigue mentioned 25 times	
Limb-Girdle muscular dystrophy						
Peric, M., et al. 2018<br><br>Quality of life in adult patients with limb-girdle muscular dystrophies<br><br>Serbia	to evaluate QoL in patients with LGMD and to identify the most significant predictors of worse QoL.	46 patients with LGMD	Prevalence and associations - fatigue as one predictor of worse QoL	Cross-sectional study with questionnaires	Fatigue not mentioned, no definition given in introduction.<br><br>Investigation of experienced fatigue one secondary aim/ outcome.<br><br>Fatigue medium focus<br>Fatigue mentioned 23 times	Standardized fatigue instrument: Krupp's Fatigue Severity Scale (FSS) where a total score ≥ 36 represented marked fatigue
Mitochondrial disease						
Bates, M.G., et al. 2013<br><br>Defining cardiac adaptations and safety of endurance training in patients with m.3243A>G-related mitochondrial disease<br><br>United Kingdom	to characterize the effects of endurance exercise training on disease burden, resting cardiac function, high energy phosphate metabolism, cardio- vascular autonomic function, fatigue and quality of life in a clinically and genetically well-characterized cohort of patients with m.3243A>G- related mitochondrial disease	Ten patients with mitochondrial disease due to the m.3243A>G mutation (6 men). Mean age 42.4.<br><br>10 age and gender matched controls.	Intervention effects	16 weeks intervention study with cycling exercise three times per week	Fatigue briefly mentioned in introduction, no definition given<br><br>Investigation of experienced fatigue one primary aim/ outcome.<br><br>Fatigue medium focus. Fatigue mentioned 23 times	Standardized fatigue instrument: Fatigue Impact Scale (FIS)
Gorman, G.S., et al. 2015<br><br>Perceived fatigue is highly prevalent and debilitating in patients with mitochondrial disease<br><br>United kingdom	to determine the magnitude and impact of self-perceived fatigue in a large, genetically heterogeneous group of patients with mitochondrial disease, whilst evaluating putative biological mechanisms that	132 patients with mitochondrial disease, 91 females, mean age 52 (18-82)	Prevalence of fatigue and associated factors	Cross sectional postal survey	Fatigue definition given in introduction:<br>"We define perceived fatigue as an overwhelming sense of tiredness, lack of energy or feeling of exhaustion (Krupp 1996), employing a multifactorial approach; distinguishing this from physiological fatigue in which the focus is muscle	Standardized fatigue instrument: Fatigue Impact Scale (FIS), score of ≥40 indicates excessive symptomatic fatigue and ≥80 severe, symptomatic fatigue.

	have been recognized in other neurological disorders and chronic disease states.				and its ability to generate and maintain power”<br><br>Investigation of experienced fatigue primary aim.<br>Fatigue major focus. Fatigue mentioned 75 times	
Karaa, A., et al. 2020<br><br>A randomized crossover trial of elamipretide in adults with primary mitochondrial myopathy<br><br>USA	to evaluate the effect of subcutaneous (SC) elamipretide dosing on exercise performance using the 6 min walk test (6MWT), patient-reported outcomes measuring fatigue, functional assessments, and safety to guide the development of the Phase 3 trial	30 patients with mitochondrial myopathy, mean age 45.3 (17-65), 25 female.	Intervention effects	Multi-centre randomized, double-blind, placebo-controlled, crossover trial	Fatigue briefly mentioned in introduction, no definition given.<br><br>Investigation of experienced fatigue secondary aim/ outcome.<br><br>Fatigue medium focus. Fatigue mentioned 34 times	Standardized fatigue instruments: The Primary Mitochondrial Myopathy Symptom Assessment (PMMSA), including a PMMSA Total Fatigue score (assessed tiredness and muscle weakness at rest and during activities)<br><br>The Neuro-QoL Fatigue Item Bank was also completed at the beginning and at the end of each treatment period
Koene, S., et al. 2013<br><br>Developing outcome measures for pediatric mitochondrial disorders: which complaints and limitations are most burdensome to patients and their parents?<br><br>The Netherlands	To investigate which complaints and disabilities patients with mitochondrial disorders and their parents experience in daily life.	78 patients with mitochondrial disorders, and their parents	Diagnostics. Development of diagnosis specific outcome measure	Cross-sectional Questionnaire study	Fatigue not mentioned in introduction, no definition given.<br><br>Investigation of experienced fatigue one secondary aim/ outcome.<br><br>Fatigue medium focus. Fatigue mentioned 39 times	Two study-specific questionnaires.<br>The first was designed to assess which symptoms were most burdensome to patients and their parents.<br>The second assessed what impairment was experienced in body functions and activities and participation in daily life.

Martens, A.M., et al.2014<br><br>Physical activity of children with a mitochondrial disease compared to children who are healthy<br><br>The Netherlands	To compare the physical activity of a group of children with mitochondrial myopathy (MM) with children who are healthy and to evaluate the suitability of different measurement tools.	Six children with MM, 2 boys, mean age 9.8 and 10 healthy children.	Prevalence/ presence of fatigue	explorative observational design with observation, tests and questionnaires	Fatigue very briefly mentioned in introduction, no definition given.<br><br>Investigation of experienced fatigue one secondary aim/ outcome.<br><br>Fatigue minor focus. Fatigue mentioned 13 times	Study-specific: During the test week, the children kept a diary, with the help of their parents and teachers, of the descriptions of their activity and the level of fatigue using smileys. The smileys had a rate from 1 (not tired at all) to 5 (very tired).
Parikh, S., et al. 2019<br><br>Fatigue in primary genetic mitochondrial disease: No rest for the weary<br><br>USA	to assess self-reported or perceived fatigue and fatigue-associated comorbid conditions in a cohort of patients with PMD to better characterize these symptoms and investigate their interrelationships. We also preliminarily assessed whether one specific measure may better gauge fatigue in patients with PMD.	48 adult patients with genetic mitochondrial diseases	Diagnostics - validation of fatigue instruments for patient group	Cross-sectional questionnaire study	Fatigue definition given in introduction:<br>"Perceived fatigue has been described as an overwhelming sense of tiredness, lack of energy, or feeling of exhaustion" (Gorman 2015, Krupp 1996).<br><br>Investigation of experienced fatigue one primary aim<br><br>Fatigue major focus. Fatigue mentioned 48 times	Several standardized fatigue instruments used:<br>Fatigue impact scale,<br>Daily fatigue impact scale<br>Fatigue severity scale,<br>Checklist individual strength
Van de Loo, K.F.E., et al. 2020<br><br>Psychological functioning in children suspected for mitochondrial disease: the need for care	to investigate the psychological well-being of children who are suspected for MD and their parents.	In total 122 children suspected for mitochondrial disease (67 boys, mean age 8.4 (0-17) and their parents participated	Prevalence of fatigue as a child symptom, reported by parents	Cros- sectional questionnaire study	Fatigue mentioned once in introduction, no definition given<br><br>Investigation of experienced fatigue one secondary aim/ outcome.<br><br>Fatigue minor focus. Fatigue mentioned 3 times	Standardized outcome instrument:<br>Behavioral problems of the child were assessed with the Child Behavior Checklist (CBCL) (parent-reported questionnaire
Verhaak, C., et al. 2016<br><br>Quality of life, fatigue and mental health in patients with the m.3243A > G mutation and its correlates with genetic characteristics	to investigate patient reported outcomes in terms of quality of life, functional impairment, fatigue and mental health. In addition, to assess the relationship between these patient reported outcomes and	72 patients with m.3243A > G mutation, 59% women, mean age 45 (19-67)	Prevalence and associations-relationship with disease manifestations and genotype	Cross-sectional questionnaire study	Fatigue discussed in introduction, no definition given.<br><br>Investigation of experienced fatigue one primary aim<br><br>Fatigue one major focus. Fatigue mentioned 58 times	Standardized fatigue instrument:<br>the Checklist Individual Strength (CIS)<br>A CIS-fatigue score of 35 or more was used to identify severe fatigue

and disease manifestation<br><br>The Netherlands	disease manifestation as well as genotype.					
Zweers, H., et al. 2020<br><br>Individual dietary intervention in adult patients with mitochondrial disease due to the m.3243 A>G mutation<br><br>The Netherlands	to evaluate the effect of an individually tailored dietary intervention on personalized goals, body composition (BC), functioning, and quality of life (QoL) in adult patients with mitochondrial disease (MD) due to the m.3243 A>G mutation.	39 patients with MD, 32 female, mean age 47. Intervention group (n = 20) with dietary intervention over a 6-mo period. The control group (n = 19) received standard care 6-mo (control period), then dietary intervention.	Intervention effects	Explorative randomized controlled trial	Fatigue briefly mentioned in introduction.<br><br>Investigation of experienced fatigue one secondary aim/ outcome.<br><br>Fatigue minor focus. Fatigue mentioned 16 times	Standardized fatigue instrument:<br>the Checklist Individual Strength fatigue questionnaire
Muscle dystrophies, mixed populations						
Ahlström, G. et al. 1994<br><br>Respiratory function, electrocardiography and quality of life in individuals with muscular dystrophy<br><br>Sweeden	to gain knowledge about the respiratory and heart functions in MD patients and to assess the extent to which these factors are associated with quality of life.	67 adults with different muscle dystrophies. 30 dystrophia myotonica, 2 myotonica congenita, 8 myopathia distalis tarda hereditaria, 4 myopathy with respiratory failure, 2 Beckers MD, 5 limb girdle MD, 3 fascio-scapulo humeral MD, 1 Emery-Deryfuss MD, 2 other MD.	Prevalence of fatigue	Cross-sectional study with questionaire	Fatigue not mentioned in introduction. No definition given<br><br>Investigation of experienced fatigue one secondary aim/ outcome.<br><br>Fatigue minor focus. Fatigue mentioned 7 times	Study-specific questionnaire. One question on fatigue: Abnormal fatigue yes/ no
Alschuler, K.N., et al. 2012.	to describe the relationship of pain and fatigue to physical functioning and depression in	332 participants with muscular dystrophy:	Prevalence	cross-sectional study, survey	Fatigue thoroughly discussed in introduction, no definition of fatigue given.	Study-specific questionnaire with: Numerical Rating Scales

Effects of pain and fatigue on physical functioning and depression in persons with muscular dystrophy<br><br>USA	persons 20-89 years old with MD.	facioscapulohumeral muscular dystrophy (FSHD) (139) and myotonic muscular dystrophy (MMD) (193). Mean age 53.2 years (20-80).			Investigation of experienced fatigue one primary aim/ outcome.<br><br>Fatigue major focus. Fatigue mentioned 82 times	(NRSs) were used to measure pain intensity and fatigue. Participants were asked to rate the severity of these symptoms over the past week on 0 to 10 scales, with 0 = ''None'' and 10 = ''Very severe.'
Boström K. & Ahlstöm, G. 2004<br><br>Living with a chronic deteriorating disease: the trajectory with muscular dystrophy over ten years<br><br>Sweeden	to elucidate experiences of living with muscular dystrophy (MD) in terms of consequences for activity, its barriers and facilitators, over a period of ten years.	46 adults with MD (13 proximal MD, 25 Myotinic dystrophy, 8 Myopathia distalis tarda hereditaria) 59% women, mean age 51, range 27 – 74.	Views and experiences	Qualitative study	Fatigue not mentioned in the introduction. Fatigue is defined as an important theme in the interviews<br><br>Investigation of experienced fatigue one secondary aim/ outcome.<br><br>Fatigue minor focus. Fatigue mentioned 6 times	Individual interviews
Cook, K.F., et al. 2012<br><br>Six Patient-Reported Outcome Measurement Information System Short Form Measures Have Negligible Age- or Diagnosis-Related Differential Item Functioning in Individuals With Disabilities<br><br>US	To evaluate the measurement invariance of 6 self-report measures selected for an ongoing longitudinal study of individuals with spinal cord injury, muscular dystrophy, postpolio syndrome, and multiple sclerosis.	2479 patients with disabilities. (spinal cord injury, muscular dystrophy, postpolio syndrome and multiple sclerosis). Separate data given on each group. 337 adults with muscular dystrophy, 58% women, mean age 53 (20-89)	Diagnostics - validation of outcome measures for a patient group	Cross- sectional mail survey	Fatigue not mentioned in introduction, no fatigue definition given<br><br>Investigation of experienced fatigue one secondary aim/ outcome.<br><br>Fatigue minor focus. Fatigue mentioned 12 times	Standardized fatigue instrument:<br>PROMIS Short forms to measure: pain interference, sleep disturbance and sleep-related impairment, satisfaction with social roles, depression, and fatigue.
Gallais, B., et al. 2015<br><br>Prevalence and correlates of apathy in	to determine the prevalence of apathy in myotonic dystrophy (DM1), to compare it with facioscapulohumeral	38 patients with DM1, mean age 36, 19 with FSHD and 20 controls	Associated factors.<br><br>Fatigue examined as one possible	Cross sectional observational study	Fatigue mentioned in introduction, no definition given.	Standardized fatigue instrument:<br>Krupp's Fatigue Severity Scale (KFSS).

myotonic dystrophy type 1<br><br>France	dystrophy (FSHD) patients and normal healthy controls, and explore its relationship to psychopathological features and cognitive function.		variable related to apathy		Investigation of experienced fatigue one secondary aim/ outcome.<br><br>Fatigue minor focus. Fatigue mentioned 21 times	Scores of ≥4 indicative of fatigue.
Gonzalez-Perez, P., et al. 2020.<br><br>Clinical and electrophysiological evaluation of myasthenic features in an alpha-dystroglycanopathy cohort (FKRP-predominant)<br><br>USA	to determine if a neuromuscular transmission defect is unique to those with *GMPPB* mutations or is also seen in a cohort of patients with α-dystroglycanopathies, most with *FKRP* mutations. and investigate if myasthenic symptoms and fatigue were associated with electro-physiologic evidence of a NMJ transmission defect in this cohort.	31 patients with alphadystro-glycanopathy (FKRP-predominant). 23 completed a fatigue survey.	Prevalence of fatigue	Cross-sectional study	Fatigue definition not described in introduction.<br><br>Investigation of experienced fatigue one primary aim/ outcome.<br><br>Fatigue medium focus. Fatigue mentioned 30 times.	Standardized fatigue instrument: Patient-Reported Outcomes Measurement Information System (PROMIS) Short Form v1.0-Fatigue 8a survey<br><br>And a modified questionnaire of myasthenic symptoms.
Jacques, M.F., et al. 2019<br><br>Quality of life in adults with muscular dystrophy<br><br>United Kingdom	1) compare the self-reported QoL of adults with different MDs a non-MD (CTRL) group; 2) present and compare between groups measures of Impairment (Muscle Strength and Activities of Daily Living) and Perception (Fatigue, Pain and Self-Efficacy); and 3) identify associations between QoL domains and measures of Impairment and Perception.	75 males with different MDs, (15 Duchenne Muscular Dystrophy, 18 Beckers Muscular Dystrophy, 12 Limb-Girdle Muscular Dystrophy, Facio-scapulohumeral Muscular Dystrophy) and 16 healthy controls	Prevalence - and associations to QoL	Cross-sectional study with questionnaire	Fatigue definition not given in introduction, fatigue briefly mentioned as common and impacting QoL.<br><br>Investigation of experienced fatigue one primary aim/ outcome.<br><br>Fatigue medium focus. Fatigue mentioned 25 times	Standardized fatigue instrument: The 8-item Fatigue Severity subscale of the Checklist Individual Strength (CIS)
Kalkman, J.S., et al. 2005<br><br>Experienced fatigue in facio-scapulohumeral dystrophy, myotonic dystrophy, and	To assess the prevalence of severe fatigue and its relation to functional impairment in daily life in patients with relatively common types of neuromuscular disorders	598 patients; 139 with FSHD (49 female, mean age 43.7, 22-61), 322 with MD (47 female, mean age 43, 18-63), 137	Prevalence of fatigue and associations - relation to functional	Cross-sectional questionnaire study	Fatigue thoroughly defined and discussed in introduction. Defined as: “an overwhelming sense of tiredness, lack of energy, and feeling of exhaustion and is not the same as weakness (Krupp 2003)”.	Standardized fatigue instrument: The Checklist Individual Strength (CIS). CIS score≥35 = severe fatigue

hereditary motor ans sensory neuropathy (HMSN-I)<br><br>The Netherlands		with HMASN-1 (41 female, mean age 42.5, 19-63)	impairment in daily life		Investigation of experienced fatigue primary aim.<br><br>Fatigue major focus. Fatigue mentioned > 70 times	
Kalkman, J.S., et al. 2006<br><br>Influence of relatives on fatigue experienced by patients with facioscapulohumeral dystrophy, myotonic dystrophy and HMSN-I<br><br>The Netherlands	to investigate the level of fatigue experienced by the close relatives and their responses to the fatigue and the neuromuscular disorder of the patient; (2) to determine if the close relatives are able to correctly perceive the level of fatigue experienced by the patient, and (3) to determine the influence of the relatives' responses and level of fatigue on the level of fatigue experienced by the patient.	One hundred and six relative and patient couples participated in this study (33 couples for FSHD patients, 32 for MD patients and 41 for HMSN patients).	Prevalence of fatigue and associations (influence of relatives response to patient fatigue on patients level of fatigue)	Cross-sectional questionnaire study	Fatigue discussed in the introduction, no definition given.<br><br>Investigation of experienced fatigue primary aim.<br><br>Fatigue major focus. Fatigue mentioned >80 times	Standardized fatigue instrument: Checklist individual strength (CIS) was used to assess the fatigue experienced by both the patients and their relatives.
Kalkmann, J.S., et al. 2007<br><br>Psychiatric disorders appear equally in patients with myotonic dystrophy, facio-scapulohumeral dystrophy, and hereditary motor and sensory neuropathy type I<br><br>The Netherlands	to determine the presence of psychiatric disorders, specifically mood disorders, anxiety disorders and substance-related disorders (lifetime and current) in patients with DM, FSHD and HMSN, and to assess if psychiatric comorbidity was related to fatigue severity and/ or muscle strength.	The patient sample consisted of 79 DM patients, 65 FSHD patients, and 73 HMSN patients.	associations between fatigue and psychiatric disorders	Cross-sectional study. Clinical interviews and self-reported questionnaires	Fatigue discussed in the introduction, no definition given.<br><br>Investigation of experienced fatigue one secondary aim.<br><br>Fatigue medium focus. Fatigue mentioned 24 times.	Standardized fatigue instrument: The Checklist Individual Strength (CIS)
Kalkman, J.S., et al. 2007<br><br>The development of a model of fatigue in neuromuscular	To present the longitudinal data of a cohort of patients with three different neuromuscular disorders during an 18-month period. Furthermore, we want to develop a longitudinal model	In total, 198 patients (60 FSHD, 70 MD and 68 HMSN patients) completed the whole study.	Associated – predicting factors to fatigue	Cross-sectional study with questionnaire	Fatigue discussed in introduction, no fatigue definition given.<br><br>Investigation of experienced fatigue primary aim.	Standardized fatigue instrument: The Checklist Individual Strength (CIS)

disorders: A longitudinal study<br><br>Netherland	to predict experienced fatigue. Based on relations between baseline variables at follow-up, we will formulate a model that will predict factors of fatigue				Fatigue major focus. Fatigue mentioned >80 times	
Kalkman, J.S., et al. 2008<br><br>Different types of fatigue in patients with facioscapulohumeral dystrophy, myotonic dystrophy and HMSN-I. Experienced fatigue and physiological fatigue<br><br>The Netherlands	To get an overview of different types of fatigue, we studied the dimensions of experienced fatigue and of physiological fatigue.	The patient sample consisted of 65 FSHD patients, 79 MD patients and 73 HMSN patients. Mean age 42.6 (range 20-61).	Associations-<br><br>(Related factors to fatigue)	Cross-sectional study. Physiological measurements and self-reported questionnaires	Fatigue definition given in introduction: "Experienced fatigue has been defined as an overwhelming sense of tiredness, lack of energy and feeling of exhaustion. It is not the same as muscle weakness or muscle fatigability. Experienced fatigue is present in more than 60% of patients with various neuromuscular disorders (Kalkman 2005)".<br><br>Investigation of experienced fatigue primary aim.<br>Fatigue major focus. Fatigue mentioned 54 times.	Standardized fatigue instrument: fatigue subscale of the Checklist Individual Strength (CIS) questionnaire.
Kuo, A., et al. 2019<br><br>Reliability and Validity of Self-Report Questionnaires as Indicators of Fatigue in RYR1-Related Disorders<br><br>USA	This study assessed the reliability and validity of two self-report questionnaires, the multidimensional fatigue inventory (MFI-20) and adult/pediatric functional assessment of chronic illness-fatigue (FACIT-F/Peds-FACIT-F) as potential fatigue measures in *RYR1*-RD affected individuals.	37 patients with RYR1-related disorder, mean age 31.5, 15 males, 9 children.	Diagnsotics - Validation of fatigue assessment tools	Questionaire study Data presented in this study were obtained during the baseline study visit (n = 37) and at 1-month follow-up (n = 18).	Fatigue definition given in introduction: "Fatigue is a multidimensional symptom that can be defined as an overwhelming, sustained sense of exhaustion, with a decreased capacity for physical and mental work and prolonged recovery after physical activity" (Filler 2014, Lai 2003, Swain 2000, Chandran 2007)<br><br>Investigation of experienced fatigue primary aim.<br>Fatigue major focus. Fatigue mentioned >100 times	Standardized fatigue instruments:<br>the multidimensional fatigue inventory (MFI-20) and adult/pediatric functional assessment of chronic illness-fatigue (FACIT-F/Peds-FACIT-F)
Lindsay, S. et al. 2019<br><br>Meaningful occupations of young adults with muscular	to explore enablers and barriers to engaging in meaningful occupations, from the perspectives of youth, parents, and practitioners	8 youths with neuromuscular disorders (5 Duchenne, 1 SMA, 1 Becker's, 1	Views and experiences	Qualitative study	Fatigue briefly mentioned in introduction, no definition given<br><br>Investigation of experienced fatigue one secondary aim/ outcome.	Semi-structured interviews

dystrophy and other neuromuscular disorders<br><br>Canada		arthrogryposis), all male, ages 19 to 28 mean 22.3 years, 11 parents and 7 practitioners.			Fatigue minor focus. Fatigue mentioned 16 times	
Meilleur, K.G., et al. 2015<br><br>Results of a two-year pilot study of clinical outcome measures in collagen VI- and laminin alpha2-related congenital muscular dystrophies<br><br>USA	to evaluate feasibility, reliability, and validity of various outcome measures, particularly the Motor Function Measure 32, in 33 subjects with COL6-RM and LAMA2-RD.	32 patients with two different muscle dystrophies (17 COL6-RM and 15 LAMA2-RD), 13 male, mean age 9.1 (range 5.0 – 19.3)	Diagnostics - Validation of fatigue assessment tools	two-year pilot study to evaluate feasibility, reliability, and validity of various outcome measures	Fatigue mentioned in introduction, no definition given<br><br>Investigation of experienced fatigue one secondary aim/ outcome.<br><br>Fatigue minor focus. Fatigue mentioned 7 times	Standardized fatigue instrument:<br><br>Patient-Reported Outcomes Measurement Information System (PROMIS) fatigue subscale
Minis M.A., et.al 2010<br><br>Employment status of patients with neuromuscular diseases in relation to personal factors, fatigue and health status: a secondary analysis<br><br>The Netherlands	To determine the number of employed people in a group of patients with neuro-muscular diseases and in 3 separate subgroups (facioscapulo-humeral dystrophy, hereditary motor and sensory neuropathy, and myotonic dystrophy) to investigate any differences in employment status between the patient groups, and to identify factors related to employment status.	591 patients with neuromuscular diseases. 138 with facioscapulo-humeral dystrophy, 135 with hereditary motor and sensory neuropathy, and 318 with myotonic dystrophy.<br>335 employed patients with NMDs, 256 not employed	Prevalence and associations	Cross-sectional questionnaire study	Fatigue definition not discussed in introduction.<br><br>Investigation of experienced fatigue one primary aim/ outcome.<br><br>Fatigue medium focus. Fatigue mentioned 30 times	Standardized fatigue instrument:<br>Checklist Individual Strength (CIS).
Minis, M.A.H., et al. 2014<br><br>How Persons with a Neuromuscular Disease Perceive Employment	to understand how people with a slow progressive adult type neuromuscular disease (NMD) (facioscapulo-humeral dystrophy (FSHD), hereditary motor and sensory neuropathy (HMSN), and myotonic dystrophy (MD))	16 adults with three different neuromuscular disorders,<br>FSHD 5, HMSN 6, MD 5. 5 women, age 24 – 62)	Views and experiences<br><br>Experiences of fatigue in relation to employment	Qualitative study	Fatigue discussed in introduction. No definition given.<br><br>Investigation of experienced fatigue one secondary/ outcome.<br>Fatigue minor focus. Fatigue mentioned 9 times	Open, in-depth interviews

Participation: A Qualitative Study<br><br>The Netherlands	perceive employment participation					
Sansone, V.A., et al. 2012<br><br>Measuring quality of life impairment in skeletal muscle channelopathies<br><br>Italy	to assess QoL in skeletal muscle channelopathies (SMC) using INQoL (individualized QoL) and SF-36 questionnaires.	66 Italian patients with SMC, 25 women and 41 men; mean total age: 43.0. (26: periodic paralysis, 36: myotonia congenita and 4: Andersen-Tawil.	Prevalence of fatigue and associations – (relation with QoL and health status)	Multicenter study – cross sectional comparing fatigue among different diagnoses	Fatigue briefly mentioned in introduction. No definition given<br><br>Investigation of experienced fatigue one secondary aim/ outcome.<br><br>Fatigue minor/ medium focus. Fatigue mentioned 15 times	Standardized fatigue instrument: INQoL, 45 questions within 10 sections covering common muscle disease symptoms like weakness, myotonia (locking), pain and fatigue.
Schillings, M.L., et al. 2007<br><br>Experienced and physiological fatigue in neuromuscular disorders<br><br>The Netherlands	This study measured the level of experienced fatigue and physiological correlates of fatigue in three genetically defined neuromuscular disorders.	Age-matched groups of three neuromuscular disorders: 65 FSHD (58.5% men, mean age 43.1), 79 classical DM (55.7 men, mean age 41), 73HMSN (41.1 % men, mean age 42.4) and 24 neurologically healthy controls.	Prevalence of fatigue and association - correlating factors to fatigue	Cohort study, experimental design	Fatigue definition discussed in introduction. “the term fatigue indicates both experienced fatigue and types of physiological fatigue. Experienced fatigue has been defined as a difficulty in initiation of or sustaining voluntary activities (Chaudhuri & Behan 2004) and an overwhelming sense of tiredness, lack of energy, and feeling of exhaustion” (Krupp & Pollina 1996).<br><br>Investigation of experienced fatigue one primary aim/ outcome.<br>Fatigue major focus. Fatigue mentioned >100 times	Standardized fatigue instruments:<br>the 4-item abbreviated fatigue questionnaire (AFQ), and the checklist Individual strength subscale fatigue (CIS-fatigue)
Smith, A.E., et al. 2014<br><br>Symptom burden in persons with myotonic and facioscapulo-humeral muscular dystrophy<br><br>USA	To examine the prevalence of pain, fatigue, imbalance, memory impairment and vision loss in persons with myotonic and facioscapulo-humeral dystrophy, and their association with functioning.	170 patients with clinically confirmed diagnosis of FSHD or DM1, mean age of the study subjects was 51.9 years (21–90 years).	Prevalence and severity of fatigue, and associations to patient functioning	Cros- sectional Questionnaire study	Fatigue discussed in introduction, no definition given.<br><br>Investigation of experienced fatigue one primary aim/ outcome.<br><br>Fatigue medium focus<br>Fatigue mentioned 20 times	Study-specific questionnaire: Presence, severity and course of five key symptoms (pain, fatigue, imbalance, memory impairment and vision loss). Severity of each symptom was assessed using a 0 (none) to 10 (very severe) Numerical Rating Scale (NRS), and

						symptom course was assessed by asking the participants to indicate, for each symptom, whether it had become worse, become better, or stayed the same since its onset, and during the past six months.
Smits, B.W., et al. 2011<br><br>Disease impact in chronic progressive external ophthalmo-plegia: more than meets the eye<br><br>The Netherlands	To determine the extent of disease impact in patients with genetically confirmed chronic progressive external ophthalmoplegia (CPEO) and compared the outcomes to those of matched myotonic dystrophy type I patients.	28 patients with CPEO, 12 men, mean age 23.6.	Prevalence of fatigue	Questionnaire study	Fatigue not mentioned or defined in introduction.<br><br>Investigation of experienced fatigue one primary aim/ outcome.<br><br>Fatigue medium focus. Fatigue mentioned 37 times.	Standardized fatigue instrument: the Checklist Individual Strength (CIS)
Statland, J.M., et al. 2012<br><br>Mexiletine for symptoms and signs of myotonia in non-dystrophic myotonia: a randomized controlled trial<br><br>4 countries (United States, Canada, England, and Italy)	To determine the effects of mexiletine for symptoms and signs of myotonia in patients with nondystrophic myotonias (NDMs)	59 patients with NDMs (34 chloride channel mutations, 21 sodium channel mutations, 4 had no mutation identified), 3 men and 26 women, with mean age of 42.9 years (range, 16-68 years)	Intervention effects	A randomized, double-blind, placebo-controlled 2-period crossover study	Fatigue briefly mentioned in introduction and methods. No definition given<br><br>Investigation of experienced fatigue one secondary aim/ outcome.<br><br>Fatigue minor focus. Fatigue mentioned 3 times	Standardized outcome instruments: the Individualized Quality of Life questionnaire for neuromuscular disorders (INQOL), including fatigue.<br>Also secondary end points included IVR-reported changes in pain, weakness, and tiredness
Trip, J., et al. 2009<br><br>Health status in non-dystrophic myotonias: close relation with pain and fatigue<br><br>The Netherlands	to investigate the impact of NDM on health status. We additionally examined the presence and effects of painful myotonia and fatigue and, finally, performed regression analyses to investigate the contribution of various determinants	62 patients with NDM (32 with chloride channel-opathies, 20 men, mean age 45.7, 30 with sodium channelopathies, 13 men, mean age 38.7)	Prevalence and associations<br><br>Prescence and level of fatigue, and relationship with physical, psychological and social functioning.	Cross-sectional questionnaire study	Fatigue mentioned in introduction, no definition given.<br><br>Investigation of experienced fatigue one primary aim/ outcome.<br><br>Fatigue major focus.<br>Fatigue mentioned 51 times.	Standardized fatigue instrument:<br>the fatigue assessment scale (FAS), FAS scores equal to or higher than 22 indicative of fatigue.

	assumed to impair the patients' physical, psychological and social functioning.					
Van Ruitenbeek, E., et al. 2019<br><br>Functional impairments, fatigue and quality of life in RYR1-related myopathies: A questionnaire study<br><br>The Netherlands	To obtain baseline data concerning the prevalence of fatigue, the psychological disease burden and quality of life associated with these common conditions,	72 patients with RYR1-related myopathies, 37 males, mean age 49.3.	Prevalence	Cross- sectional Questionnaire study	Fatigue briefly mentioned in introduction, no definition given<br><br>Investigation of experienced fatigue one primary aim/ outcome.<br><br>Fatigue one major focus<br>Fatigue mentioned 48 times	Standardized fatigue instrument:<br>Checklist individual strength CIS<br>A CIS-fatigue score of 35 or more was used to identify severe fatigue
Veenhuizen, Y. et al. 2019<br><br>Self-management program improves participation in patients with neuromuscular disease: A randomized controlled trial<br><br>The<br>Netherlands	To investigate the effectiveness of Energetic, a self-management group program combining aerobic training, energy conservation management, and relapse prevention to improve social participation in patients with neuromuscular disease (NMD) and chronic fatigue.	53 patients with 17 various types of NMD and chronic fatigue were randomly allocated to Energetic, a 4-month group intervention, or to usual care. 29 in intervention group, 8 males, mean age 52 (37-63). 24 in control group, 9 males, mean age 50 (41-60).	Intervention effects	Multicenter, assessor-blinded, 2-armed randomized controlled trial with repeated measurements.	Fatigue discussed given in introduction, no definition given.<br><br>Investigation of experienced fatigue one secondary aim/ outcome.<br><br>Fatigue medium focus<br>Fatigue mentioned 39 times	Standardized fatigue instrument: Secondary outcome- fatigue: Checklist Individual Strength (CIS), subscale fatigue.<br><br>COPM-performance as the primary outcome.<br>One
Vincent, K.A., et al. 2007<br><br>Construction and validation of a quality of life questionnaire for neuromuscular disease (INQoL)<br><br>UK	to develop and validate an individualized muscle disease specific measure of QoL for adults suitable for both clinical and research use.	252 responders with different neuromuscular diseases (congenital myopathies, limbgirdle MD, facioscapulohumer al MD, dystrophic and nondystrophic myotonias, and	Diagnostics - Development and validity of outcome measure (including fatigue), for a patient group.	A literature review, qualitative interviews and a postal survey	Fatigue not mentioned in introduction.<br><br>Investigation of experienced fatigue one secondary aim/ outcome.<br><br>Fatigue minor focus<br>Fatigue mentioned 15 times	Development of a disease specific measure of QoL, INQoL – wich has one section on fatigue- INQoL Fatigue score

		inflammatory myopathies), 183 women, mean age 52.6 (16 – 96).				
Werlauff, U., et al. 2014<br><br>Fatigue in patients with spinal muscular atrophy type II and congenital myopathies: evaluation of the fatigue severity scale<br><br>Denmark	to evaluate whether the fatigue severity scale (FSS) is an appropriate instrument to assess fatigue in patients with spinal muscular atrophy type II (SMA II) and congenital myopathies (CM).	29 SMA II patients (10 F/19 M) and 71 CM patients (36 F/35 M).<br>Recruited from two studies by the Danish National Rehabilitation Center for Neuromuscular Diseases and a neuromuscular research unit	Diagnostics - validation of fatigue instrument<br><br>Evaluation of the appropriateness of FSS for these patient groups	Questionnaire study and focus group interviews<br><br>Mixed methods study	Fatigue definition thoroughly discussed in introduction: "The psychological component, the perception and experience of fatigue, can be defined as an overwhelming feeling of tiredness, lack of energy, and difficulties in initiating or sustaining activities, mentally, physically, or both. " (Chaudhuri 2004, Krupp 19789, ENMC 2011)<br><br>Investigation of experienced fatigue primary aim.<br><br>Fatigue major focus.<br>Fatigue mentioned 186 times	Standardized fatigue instrument: Fatigue severity scale
Myotonic dystrophies						
Baldanzi, S., et al. 2017<br><br>The proposal of a clinical protocol to assess central and peripheral fatigue in myotonic dystrophy type 1<br><br>Italy	to get an overview of central and peripheral components of fatigue in a sample of patients meeting both genetic and clinical criteria for DM1, through a specifically designed multidimensional protocol.	26 DM1 patients (17 males, 9 females, mean age 41.6 years, range 19-67).	Prevalence of both muscle fatigue and experienced fatigue, but main focus on muscle fatigue	Cross-sectional cohort study	Thorough definition on central and peripheral muscle fatigue in introduction, but no definition of experienced fatigue<br><br>Investigation of experienced fatigue one primary aim/ outcome.<br>Patient- perceived experienced fatigue medium focus. Muscular fatigue major focus, fatigue mentioned 60 times.	Standardized fatigue instrument:<br><br>Fatigue Severity Scale
Fujino, H., et al. 2018<br><br>Cognitive impairment and quality of life in patients with myotonic dystrophy type 1	to describe the affected domains of cognitive functioning in patients with DM1 and evaluate the relationships between	60 patients with DM1, 35 men. Mean age 47.1	Prevalence of fatigue and associations to other factors	Cross-sectional study with questionnaire	Fatigue briefly mentioned in introduction, no definition given.<br><br>Investigation of experienced fatigue one secondary aim/ outcome.	Standardized fatigue instrument: Multidimensional Fatigue Inventory (MFI),

Japan	cognitive functioning, psychological factors, and QoL				Fatigue medium focus. Fatigue mentioned 20 times.	
Gagnon, C., et al. 2008<br><br>Predictors of disrupted social participation in myotonic dystrophy type 1<br><br>Canada	To identify personal and environmental predictors of the most disrupted participation domains in people with myotonic dystrophy type 1 (DM1).	200 adults with DM1, 121 women, mean age 47	Fatigue as one associated factor/ possible explanatory variable to participation in DM1	Cross-sectional questionnaire study.	Fatigue briefly mentioned in introduction, no definition given<br><br>Investigation of experienced fatigue one primary aim/ outcome.<br>Fatigue medium focus. Fatigue mentioned 35 times.	Standardized fatigue instrument:<br>Krupp Fatigue Severity Scale
Gallais, B., et al. 2017<br><br>Further evidence for the reliability and validity of the Fatigue and Daytime Sleepiness Scale<br><br>Canada	to gain further evidence of the psychometric properties of the FDSS (intra-rater reliability, internal consistency, the precision (standard error of measurement (SEM)), the minimum detectable change (MDC) and construct validity) in patients affected by DM1.	48 patients with DM1, 22 men, mean age 44,2 (range 20-64)	Diagnostics-Validation of fatigue instrument for a patient group.	Test-retest Reliability study	Fatigue defined in the introduction: “Fatigue is characterized by a subjective lack of physical and/or mental energy and is more common in DM1 than in other neuromuscular disorders” (Kalkman 2005).<br><br>Investigation of experienced fatigue one primary aim/ outcome.<br>Fatigue major focus. Fatigue mentioned 37 times.	Standardized fatigue instrument: Disease specific instrument for DM1: Fatigue and Daytime Sleepiness Scale
Gliem, C., et al. 2019<br><br>Tracking the brain in myotonic dystrophies: A 5-year longitudinal follow-up study<br><br>Germany	to examine the natural history of brain involvement in adult-onset myotonic dystrophies type 1 and 2 (DM1, DM2).	Patients assessed to times: 16 DM1 patients: 6 males, mean age 42,5 and 16 DM2 patients; 9 males, mean age 48.2, and 17 healthy controls	Prevalence of fatigue as one symptom in DM1 and 2	Longitudinal observational study	Fatigue briefly mentioned in introduction, no definition given,<br><br>Investigation of experienced fatigue one primary aim/ outcome.<br>Fatigue minor/ medium focus. Fatigue mentioned 17 times.	Standardized fatigue instrument:<br>Krupp Fatigue Severity Scale
Hagerman, K.A., et al. 2019<br><br>The myotonic dystrophy experience: a North American cross-sectional study<br><br>USA and Canada	to identify and quantitate the issues of greatest importance for patients with myotonic dystrophies and their caregivers	1180 patients with DM, 59% women, mean age 45 (range 0-86)	Prevalence and impact of fatigue symptoms	Questionnaire study	Fatigue briefly mentioned in introduction, no definition given.<br><br>Investigation of experienced fatigue one secondary aim/ outcome.<br>Fatigue minor focus. Fatigue mentioned 4 times.	Study-specific questionnaire. Symptomatic questions covered areas related to muscle, gastrointestinal function, cardio-respiratory function, sleep, fatigue, and cognitive function.

						Respondents rated from 1-4 if the symptom had “no impact,” “minor impact,” “moderate impact,” or “major impact” on their life.
Heatwole, C., et al. 2012<br><br>Patient-reported impact of symptoms in myotonic dystrophy type 1 (PRISM-1)<br><br>USA	To determine the most critical symptoms in a national myotonic dystrophy type 1 (DM1) population and to identify the modifying factors that have the greatest effect on the severity of these symptoms.	Phase 1: 20 adults with DM1<br><br>Phase 2: 278 DM1 patients, 147 male, mean age 47 (21-73)	Prevalence and associated factors<br><br>Prevalence and impact of symptoms and modifying factors	Phase 1. Qualitative interviews and<br><br>Phase 2: Cross sectional survey study	Fatigue briefly mentioned in introduction no definition given<br><br>Investigation of experienced fatigue one secondary aim/ outcome. Fatigue Minor focus. Fatigue mentioned 12 times	Study-specific questionnaire: symptoms of importance recorded with a 6-point Likert scale: 1= I don’t experience this; 2= I experience this but it does not affect my life; 3 = It affects my life a little; 4 = It affects my life moderately; 5= It affects my life very much; and 6 = It affects my life severely
Heatwole, C., et al. 2015<br><br>Patient-Reported Impact of Symptoms in Myotonic Dystrophy Type 2 (PRISM-2)<br><br>USA<br><br>Neurology	To determine the frequency and relative importance of the most life-affecting symptoms in myotonic dystrophy type 2 (DM2) and to identify the factors that have the strongest association with these symptoms.	Phase 1: 15 DM2 patients.<br>Phase 2. 74 DM2 patients, 26 male, mean age 57 (27-82). Recruited from the national registry of patients with DM2	Prevalence and impact of symptoms and associated factors	Phase 1. Qualitative interviews and<br><br>Phase 2: Cross sectional survey study	Fatigue definition not given in introduction.<br><br>Investigation of experienced fatigue one secondary aim/ outcome.<br><br>Fatigue minor focus. Fatigue mentioned 15 times	Study-specific questionnaire: symptoms of importance recorded with a 6-point Likert scale: 1= I don’t experience this; 2= I experience this but it does not affect my life; 3 = It affects my life a little; 4 = It affects my life moderately; 5= It affects my life very much; and 6 = It affects my life severely
Hermans, M.C., et al. 2013<br><br>Fatigue and daytime sleepiness scale in myotonic dystrophy type 1	to construct a combined scale for assessing fatigue and daytime sleepiness [i.e., the Fatigue and Daytime Sleepiness Scale (FDSS)] for DM1	354 patients with DM1. Dutch patients: 167, 81 female, mean age 4.1 (18-69). Canadian patients 187, 115 female,	Diagnostics - Development and validation of fatigue instrument for a patient group	Questionnaire survey	Fatigue definition given in introduction: “DM1-related fatigue is characterized by a subjective lack of physical and/or mental energy. Although fatigue is an important symptom in any progressive physically disabling disease, it is more common	Standardized disease specific fatigue instrument:<br>Fatigue and Daytime Sleepiness Scale (FDSS)]composed of items from the Epworth

Netherlands and Canada		mean age 46 (20-80).			in DM1 than in other neuromuscular disorders and may even be prominent when muscular impairment is relatively mild"(Kalkman 2005).<br><br>Investigation of experienced fatigue one primary aim/ outcome.<br>Fatigue major focus. Fatigue mentioned 57 times	sleepiness scale, the Daytime sleepiness scale and Fatigue Severity scale)
Johnson, N.E., et al. 2016<br><br>Parent-reported multi-national study of the impact of congenital and childhood onset myotonic dystrophy<br><br>USA, Canada, Sweeden	to better understand DM1 disease burden and the relationship between disease manifestations and a patient's quality of life as reported by DM1 parents.	150 parents answered, for 150 children with DM1, 61.3% male, mean age 17.9	Prevalence of fatigue symptoms and impact on child's life	Questionnaire study	Fatigue briefly mentioned in introduction, no definition given.<br><br>Investigation of experienced fatigue one secondary aim/ outcome.<br>Fatigue minor focus. Fatigue mentioned 8 times	Study- specific instrument: Prevalence of each symptom and theme were calculated. Parents were asked to report and rate impact of their child's symptoms on a 0-4 scale: (0='My child experiences this but it does not affect his or her life'; 4='It affects my child's life severely').
Kierkegaard, M., et al. 2011<br><br>Functioning and disability in adults with myotonic dystrophy type 1<br><br>Sweeden	to describe and analyse functioning and the presence of concurrent body-function impairments, activity limitations and participation restrictions Further to explore associations of measures of manual dexterity and of walking capacity with measures of activities and participation.	70 patients with DM1, 41 women and 29 men, mean age was 45 years, SD 13,(19 to 70).	Prevalence of fatigue and associations to other factors	Cross-sectional study, with standardized examinations, tests and questionnaires	Fatigue not mentioned in introduction, no definition given<br><br>Investigation of experienced fatigue one secondary aim/ outcome.<br>Fatigue minor focus. Fatigue mentioned 8 times	Standardized fatigue instrument:<br>Fatigue Severity Scale<br>A score equal to and above four is suggested to indicate the presence of fatigue
Knak, K.L., et al. 2020<br><br>Physical activity in myotonic dystrophy type 1<br><br>Denmark	to investigate physical activity and to investigate the predictors of physical activity in individuals with DM1 compared to healthy controls.	67 patients with DM1, 32 female, mean age 41	Prevalence and associations<br><br>(Fatigue as one predictor to physical activity)	Observational study with one visit	Fatigue not mentioned in introduction, no definition given,<br><br>Investigation of experienced fatigue one secondary aim/ outcome.<br>Fatigue minor focus. Fatigue mentioned 15 times	Standardized fatigue instrument:<br>Fatigue Severity Scale-7

Laberge, L., et al. 2005<br><br>Fatigue and daytime sleepiness rating scales in myotonic dystrophy: a study of reliability<br><br>Canada	To assess the reliability of the Epworth Sleepiness Scale (ESS), Daytime Sleepiness Scale (DSS), Chalder Fatigue Scale (CFS), and Krupp's Fatigue Severity Scale (KFSS) in patients with myotonic dystrophy type 1 (DM1).	27 patients with DM1 (11 men, 16 women) aged 37–66 years (mean 49.6)	Diagnostics- Validation of fatigue instruments for a patient group<br><br>Psychometric properties of fatigue scales	Questionnaire study, psychometric properties	Fatigue briefly discussed in introduction.<br><br>Investigation of experienced fatigue one primary aim/ outcome.<br>Fatigue major focus. Fatigue mentioned 38 times.	Standardized fatigue instruments: Chalder Fatigue Scale (CFS), and Krupp's Fatigue Severity Scale (KFSS
Laberge, L., et al. 2009<br><br>Fatigue and daytime sleepiness in patients with myotonic dystrophy type 1: to lump or split?<br><br>Canada	To assess the relationship and clinical correlates of fatigue and Excessive Daytime Sleepiness (EDS) in 200 myotonic dystrophy type 1 (DM1) patients	The study cohort included two-hundred patients with adult DM1 (79 men, 121 women; 47.0 ± 11.8 years, range 20–81)	Prevalence of fatigue and associations<br><br>correlation to daytime sleepiness	Questionnaire study	Fatigue phenomenon discussed in introduction, no definition given.<br><br>Investigation of experienced fatigue one primary aim/ outcome.<br>Fatigue major focus. Fatigue mentioned >100 times	Standardized fatigue instruments:<br>Krupp's Fatigue Severity Scale (FSS), and a Visual Analog Scale (VAS) for fatigue.
Laberge, L., et al. 2013<br><br>Clinical, psychosocial, and central correlates of quality of life in myotonic dystrophy type 1 patients<br><br>Canada	to compare SF-36 scores of a large cohort of DM1 patients to population specific normative data and to identify demographic, social, clinical, genetic, central, affective, and personality factors that contribute to the SF-36 physical (PCS) and mental (MCS) component summary scores, respectively representing physical and mental health function	200 adults with DM1 (79 men, 121 women; mean age 47.0 years)	Associated factors<br><br>(Correlations of fatigue to quality of life)	Questionnaire study	Fatigue not defined/ discussed in introduction,<br><br>Investigation of experienced fatigue one secondary aim/ outcome.<br><br>Fatigue medium focus. Fatigue mentioned 18 times	Standardized fatigue instrument:<br>Fatigue Severity Scale (FSS), Scores of 4 or higher are considered as indicative of pathological or excessive fatigue
Laberge, L., et al. 2020<br><br>Predicting daytime sleepiness and fatigue: a 9-year prospective study in myotonic dystrophy type 1<br><br>Canada	to determine factors that may influence levels of daytime sleepiness and fatigue in a large cohort of DM1 patients followed for 9 years.	115 DM1 patients, 72 female, mean age T1 43.6, T2 52.3	Prevalence and associations<br><br>Prevalence of excessive fatigue<br>Explore factors that may influence fatigue	A longitudinal design was used to collect data in 115 DM1 patients at baseline T1: (2002–2004) and at follow-up T2: (2011–2013),	Fatigue mentioned in introduction, no definition given.<br><br>Investigation of experienced fatigue one primary aim/ outcome.<br>Fatigue major focus. Fatigue mentioned 75 times	Standardized fatigue instrument:<br>Fatigue Severity Scale (FSS). FSS score of 4 or higher is considered as indicative of excessive fatigue

Lam, E.M., et al. 2013<br><br>Restless legs syndrome and daytime sleepiness are prominent in myotonic dystrophy type 2<br><br>USA	To determine the frequency of sleep disturbances in DM2.	54 patients with genetically confirmed DM2, and 104 controls	Prevalence of fatigue compared to controls	Questionnaire study	Fatigue briefly discussed in introduction, no definition given.<br><br>Investigation of experienced fatigue one primary aim/ outcome.<br>Fatigue medium focus. Fatigue mentioned 24 times	Standardized fatigue instrument: Fatigue Severity Scale, scores of 4 or greater suggest problematic fatigue symptoms.
Landfeldt, E., et al. 2019<br><br>Disease burden of myotonic dystrophy type 1<br><br>United Kingdom	to investigate the disease burden of myotonic dystrophy type 1 (DM1).	60 patients with DM1 (mean age: 45 years; 45% female).	Prevalence and associations impact of fatigue	Questionnaire study,	Fatigue not mentioned in introduction, no definition given,<br><br>Investigation of experienced fatigue one primary aim/ outcome.<br>Fatigue minor/ medium focus. Fatigue mentioned 15 times	Standardized disease specific measure: The Individualized Neuromuscular Quality of Life INQoL, including fatigue.
Lecordier, D., et al. 2017<br><br>Understanding people with Steinert's disease to better care for them<br><br>France	to understand the life experiences of individuals with Steinert's disease, by looking at the limitations of their bodies and the adaptation strategies they develop	23 interviews with adult-onset patients with Steinert's disease (DM1) and their close relations, alone or in groups parent/child, couple), in their homes. Patients were aged between 34 and 73.	Views and experiences	Qualitative study	Fatigue briefly mentioned in introduction, no definition given.<br><br>Investigation of experienced fatigue one secondary aim/ outcome.<br>Fatigue minor focus. Fatigue mentioned 10 times	Individual interviews and observations of multidisciplinary consultations
MacDonald, J.P., et al. 2002. Modafinil reduces excessive somnolence and enhances mood in patients with myotonic dystrophy.<br><br>Canada	To evaluate the potential of modafinil in reducing excessive daytime somnolence (EDS) and enhancing indexes of quality of life and mood in patients with myotonic dystrophy (DM).	Forty DM patients, 27 women, mean age 40.7	Intervention effects	Randomized double-blind, cross-over design	Fatigue not mentioned in introduction, no definition given.<br><br>Investigation of experienced fatigue one secondary aim/ outcome.<br>Fatigue minor focus. Fatigue mentioned 8 times	Standardized fatigue instrument: Fatigue–inertia factors of the Profile of Mood States18 (POMS)

Minnerop, M., et al. 2011<br><br>The brain in myotonic dystrophy 1 and 2: evidence for a predominant white matter disease<br><br>Germany	to analyse if abnormal white matter integrity was associated with distinct clinical parameters and neuro-psychological performance; and whether brain morphological changes play a direct causative role in the development of mood disturbances and increased daytime sleepiness or fatigue	22 patients with myotonic dystrophy type 1 (male/ female: 9/13, mean age 43.1) and 22 patients with myotonic dystrophy type 2 (male/ female: 12/10, mean age 52.5) as well as age- and sex-matched healthy controls	Prevalence of fatigue and associations of fatigue to neuropsychological performance	Cross-sectional observational	Fatigue briefly mentioned in introduction, definition not given.<br><br>Investigation of experienced fatigue one secondary aim/ outcome. Fatigue medium focus. Fatigue mentioned 33 times	Standardized fatigue instrument:<br>Krupps Fatigue Severity Scale
O'Donoghue, F.J., et al. 2017<br><br>Effects of 1-month withdrawal of ventilatory support in hypercapnic myotonic dystrophy type 1<br><br>France	to determine the effects of elective discontinuation of ventilatory support for 1 month in DM1 patients receiving NIV for chronic hypercapnic respiratory failure.	12 DM1 patients, 5 males, mean age 49.53	Intervention effects	A pilot 1-month open-label NIV withdrawal study	Fatigue not mentioned or defined in introduction<br><br>Investigation of experienced fatigue one secondary aim/ outcome. Fatigue minor focus. fatigue mentioned 10 times	Standardized fatigue instrument:<br>Fatigue Severity Scale
Okkersen, K., et al. 2018<br><br>Cognitive behavioural therapy with optional graded exercise therapy in patients with severe fatigue with myotonic dystrophy type 1: a multicentre, single-blind, randomised trial<br><br>France, Germany, Netherlands, UK	To determine whether cognitive behavioural therapy optionally combined with graded exercise compared with standard care alone improved the health status of patients with myotonic dystrophy type 1.	255 DM1 patients: 128 patients in the cognitive behavioural therapy intervention (58 female, mean age 44.8) and 127 in standard care alone (60 female, mean age 46.4)	Intervention effects	Multicenter single-blind, randomized trial	Fatigue discussed in introduction, no definition given.<br><br>Investigation of experienced fatigue one secondary aim/ outcome. Fatigue medium/ major focus. Fatigue mentioned 50 times	Standardized fatigue instruments:<br>Fatigue and daytime sleepiness scale and Checklist-individual strength (CIS)-fatigue

Peric, S., et al. 2013<br><br>Influence of multisystemic affection on health-related quality of life in patients with myotonic dystrophy type 1<br><br>Serbia	To assess health-related quality of life (HRQoL) in patients with DM1, to identify muscular, multisystemic, central and social factors that may affect QoL and to define a DM1 patient in risk of poor QoL	120 DM1 patients, 70 men, mean age 46.4	Prevalence of fatigue and associations (relationship to QoL)	Cross-sectional questionnaire study	Fatigue not mentioned in introduction, no definition given<br><br>Investigation of experienced fatigue one secondary aim/ outcome.<br>Fatigue minor focus. Fatigue mentioned 17 times	Standardized fatigue instrument:<br>Krupp's Fatigue Severity Scale (FSS).<br>A score of 36 and above (out of a maximum of 63) indicated the presence of significant fatigue
Peric, S., et al. 2014<br><br>Transcranial sonography in patients with myotonic dystrophy type 1<br><br>Serbia	To analyze transcranial sonography (TCS) in patients with myotonic dystrophy type 1	61 DM1 Patients (52,5% men, mean age 41.2), and 55 matched healthy controls	Prevalence and associations	Cross-sectional study with questionnaires	Fatigue briefly mentioned in introduction, no definition given.<br><br>Investigation of experienced fatigue one secondary aim/ outcome.<br><br>Fatigue minor focus fatigue mentioned 15 times	Standardized fatigue instrument:<br>Krupp Fatigue Severity Scale (FSS).
Peric, S., et al. 2019<br><br>Fatigue in myotonic dystrophy type 1: a seven-year prospective study<br><br>Serbia	The aim of this research was to assess frequency of fatigue in DM1 patients during a seven-year period.	64 DM1 patients at baseline (50% males, age 42 ± 12 years), and 38 after seven years.	Prevalence and associations<br><br><br>Level of fatigue and development over 7 years, and associations to other variables	Prospective study, measurement with questionnaires at baseline and after 7 years	Fatigue definition given in introduction: "Fatigue may be defined as a subjective feeling of a lack of physical and/or mental energy that only partially withdraws after rest" (Kalkman 2005).<br><br>Investigation of experienced fatigue primary aim.<br>Fatigue major focus. Fatigue mentioned 99 times	Standardized fatigue instrument:<br>Krupp's Fatigue Severity Scale (FSS).
Pincherle, A., et al. 2012<br><br>Sleep breathing disorders in 40 Italian patients with Myotonic dystrophy type 1<br><br>Italy	to estimate the prevalence and nature of sleep breathing disorders in DM1, and determine whether there is a relationship between sleep breathing disorders and clinical parameters such as pulmonary function, degree of neuromuscular impairment, daytime sleepiness, and fatigue	24 men and 16 women with DM1; mean age 38.6.	Prevalence and associations<br>Prevalence of fatigue and relationship with sleep breathing disorders	Cross sectional study Clinical evaluation and questionnaires	Fatigue briefly mentioned in introduction, no definition given.<br><br>Investigation of experienced fatigue one secondary aim/ outcome.<br>Fatigue minor focus<br>Fatigue mentioned 16 times	Standardized fatigue instrument:<br>Krupp's Fatigue Severity Scale (FSS). A cut-off of 36 at FSS was used to define fatigue

Quera Salva, M.A., et al. 2006<br><br>Sleep disorders in childhood-onset myotonic dystrophy type 1<br><br>France	to obtain objective measurements of sleepiness and to look for sleep-related breathing disorders that might cause fatigue and sleepiness in young patients with childhood-onset DM1.	Twenty-one patients were included. Mean age was 15.0 ± 3.0.	Prevalence of fatigue/ somnolence	Prospective study (consecutive enrolment) with sleep study	Fatigue mentioned in introduction, no definition given.<br><br>Investigation of experienced fatigue one secondary aim/ outcome. Fatigue medium focus. Fatigue mentioned 26 times	Study-specific methods: Patients underwent a clinical and neuro-psychological evaluation, and asked an open question if they felt fatigue and/or somnolent on a daily basis.
Rakocevic-Stojanovic, V., et al. 2014<br><br>Significant impact of behavioral and cognitive impairment on quality of life in patients with myotonic dystrophy type 1<br><br>Serbia	To assess an impact of cognitive and behavioral impairment on QoL in a larger cohort of patients with DM1.	22 patients with juvenile DM1 (63.6 % males, mean age 33.2) and 44 patients with adult DM1 (43.2% males mean age 46.2).	Prevalence of fatigue and associations – (relationship to quality of life)	Clinical examinations and questionnaires	Fatigue briefly mentioned in introduction. No definition given.<br><br>Investigation of experienced fatigue one secondary aim/ outcome. Fatigue medium focus. Fatigue mentioned 26 times	Standardized fatigue instrument:<br>Krupps fatigue severity scale.
Rakocevic-Stojanovic, V., et al. 2016a<br><br>Brain sonography insight into the midbrain in myotonic dystrophy type 2<br><br>Serbia	to analyze transcranial sonography (TCS) findings in genetically confirmed myotonic dystrophy type 2 (DM2) patients.	40 genetically confirmed DM2 patients (27.5% men, mean age 51.4)	Prevalence of fatigue and associations- (relationship to brain sonography findings)	Clinical examinations and questionnaires	Fatigue briefly mentioned in introduction. No definition given.<br><br>Investigation of experienced fatigue one secondary aim/ outcome.<br><br>Fatigue medium focus. Fatigue mentioned 24 times	Standardized fatigue instrument: Krupps Fatigue Severity scale. A score ≥36 indicated the presence of significant fatigue.
Rakocevic-Stojanovic, V., et al. 2016b<br><br>Quality of life in patients with myotonic dystrophy type 2<br><br>Serbia	To analyze quality of life (QoL) in a large cohort of myotonic dystrophy type 2 (DM2) patients in comparison toDM1 control group using both generic and disease specific questionnaires. In addition, we intended to identify different factors that might affect QoL of DM2 subjects.	49 DM2 patients (30.6% males, mean age 51.4) were compared with 42 adult-onset DM1 patients (40.5 % males, mean age 47.3).	Prevalence of fatigue and associations- (relationship to quality of life)	Clinical examinations and questionnaires	Fatigue not mentioned in introduction. No definition given.<br><br>Investigation of experienced fatigue one secondary aim/ outcome. Fatigue medium focus<br>fatigue mentioned 17 times	Standardized fatigue instrument: Krupps fatigue severity scale (FSS). A score ≥36 indicated the presence of significant fatigue.

Symonds, T., et al. 2017<br><br>Review of patient-reported outcome measures for use in myotonic dystrophy type 1 patients	To (1) identify available measures for DM1 specific to assessing symptoms, ADL, HRQOL, fatigue, and sleep; and to (2) review evidence of the reliability and validity of these assessments.	11 articles met the inclusion criteria for this review, two regarding fatigue instruments.	Diagnostics; validation of assessment tools	Systematic review of Pubmed/ medline, to Identify available measures for PROs like fatigue used in DM1 populations	Fatigue mentioned in introduction. No definition given<br><br>Investigation of experienced fatigue one primary aim/ outcome.<br><br>Fatigue medium focus.<br>Fatigue mentioned 23 times	
Tieleman , A.A., et al. 2010<br><br>Poor sleep quality and fatigue but no excessive daytime sleepiness in myotonic dystrophy type 2<br><br>The Netherlands	To investigate the frequency of EDS, poor sleep quality and fatigue in DM2.	29 DM2 patients 20 females, mean age 53.2 (28-71), and 29 controls (age and gender matched DM1 patients.	Prevalence of fatigue and associations (relationship with excessive daytime sleepiness)	Cross-sectional Questionnaire study	Fatigue mentioned in introduction. No definition given.<br><br>Investigation of experienced fatigue one primary aim/ outcome.<br><br>Fatigue major focus. Fatigue mentioned 46 times.	Standardized fatigue instrument: Checklist Individual Strength (CIS). Scores of 35 or more are considered to indicate severe fatigue
Tieleman, A.A., et al. 2011<br><br>High disease impact of myotonic dystrophy type 2 on physical and mental functioning<br><br>The Netherlands	to investigate health status in patients with myotonic dystrophy type 2 (DM2) and determine its relationship to pain and fatigue.	29 DM2 patients 20 females, mean age 53.2 (28-71), and 29 controls (age and gender matched DM1 patients.	Prevalence of fatigue and associations to health status	Questionnaire study	Fatigue mentioned in introduction. No definition given<br><br>Investigation of experienced fatigue one secondary aim/ outcome.<br><br>Fatigue medium focus.<br>Fatigue mentioned 39 times.	Standardized fatigue instrument: Checklist Individual Strength (CIS). Scores of 35 or more are considered to indicate severe fatigue
Van der Werf, S., et al. 2003<br><br>The relation between daytime sleepiness, fatigue, and reduced motivation in patients with adult onset myotonic dystrophy	to test the relations between excessive daytime sleepiness, experienced fatigue, and reduced motivation.	32 patients (16 female/16 male), mean age 43.8 years (range 22 to 73).	Associations to excessive daytime sleepiness	Cross-sectional Questionnaire study	Fatigue briefly mentioned in introduction, no definition given<br><br>Investigation of experienced fatigue one secondary aim/ outcome.<br><br>Fatigue medium focus. Fatigue mentioned 20 times	Standardized fatigue instrument: The subscale "fatigue severity" of the checklist individual strength (CIS).

Van Dorst, M., et al. 2019<br><br>Structural white matter networks in myotonic dystrophy type 1<br><br>The Netherlands	To test the hypothesis that altered white matter microstructural integrity and network organization was present in a cohort of individuals with DM1 compared to unaffected controls, which was expected to be associated with CNS related disease manifestations of DM1.	26 adults with DM1, 13 men, mean age 46.	Prevalence and associations<br><br>Presence and level of experienced fatigue and relation to altered brain white matter	Cross-sectional study with neuropsychological assessment and brain MRI	Fatigue mentioned in introduction, no definition given.<br><br>Investigation of experienced fatigue one secondary aim/ outcome.<br><br>Fatigue minor focus, fatigue mentioned 20 times.	Standardized fatigue instrument: Checklist individual strength fatigue subscale: CIS-fatigue
Van Heugten, C., et al. 2018<br><br>Participation and the Role of Neuro-psychological Functioning in Myotonic Dystrophy Type 1<br><br>The Netherlands	To investigate the level of participation of DM1 patients and their partners and the determinants.	Sixty-six patients with DM1, 54.5% men, mean age 47. 55 caregivers also participated.	Prevalence and associations (relationship to participation)	Cross-sectional study	Fatigue mentioned in introduction, no definition given.<br><br>Investigation of experienced fatigue one secondary aim/ outcome.<br><br>Fatigue medium focus. Fatigue mentioned 27 times	Standardized fatigue instrument: Fatigue Severity Scale (FSS). A score equal to or above 4 indicates a moderate to high impact of fatigue on daily living
White, M. 2020<br><br>Patient Input to Inform the Development of Central Nervous System Outcome Measures in Myotonic Dystrophy<br><br>USA	To describe CNS disease symptoms, their impact on quality of life, and potential CNS-related targets that they considered important for drug development consideration.	Some 350 members of the myotonic dystrophy community.	Views and experiences<br><br>Experiences with fatigue and description of symptoms	Qualitative study	Fatigue mentioned in introduction, no definition given.<br><br>Investigation of experienced fatigue one secondary aim/ outcome.<br><br>Fatigue minor focus. Fatigue mentioned 14 times	Analysis of group session reports from a patient association conference
Winblad, S. & Lindberg, C. 2019<br><br>Perceived fatigue in myotonic dystrophy type 1: a case-control study<br><br>Sweeden	to explore perceived fatigue, experienced functional limitations due to fatigue and clinical correlates in patients with Myotonic Dystrophy type 1 (DM1).	32 consecutive patients with DM1 (14 women and 18 men) (age range 23–61 years) and 30 sex, age and education matched healthy control subjects	Prevalence and associations<br><br>Prevalence of perceived fatigue and clinical correlates to fatigue	Cross-sectional, questionnaire study	Fatigue definition given in introduction: "fatigue is commonly defined as an overwhelming sense of tiredness, lack of energy and feeling of exhaustion associated with difficulties in initiating or sustaining voluntary activities (Finsterer 2014)".	Standardized fatigue instrument:<br>Fatigue Impact Scale (FIS).

					Investigation of experienced fatigue primary aim.<br><br>Fatigue major focus.<br>fatigue mentioned 60 times	
Wood, L., et al. 2017<br><br>The UK Myotonic Dystrophy Patient Registry: facilitating and accelerating clinical research<br><br>UK	to report the demographic and clinical findings for the 556 symptomatic DM1 patients registered by july 2016	556 symptomatic DM1 patients. An almost even distribution was seen between genders and a broad range of ages was present from 8 months to 78 years	Prevalence and associations	Cross-sectional study with registry data	Fatigue not mentioned in introduction, no definition given.<br><br>Investigation of experienced fatigue one primary aim/ outcome.<br><br>Fatigue medium focus.<br>fatigue mentioned 25 times	Registry specific questions on Fatigue / sleepiness: - You're your fatigue/ sleepiness. Severe/ Mild/ No. -Does fatigue or daytime sleepiness currently have a negative effect on your normal daily activities? Yes, severely/ Yes, but only mildly/Not at all. -Fatigue medication use?<br>Yes/ No/ Unknown. -Do you currently take any medication to treat or prevent fatigue or daytime sleepiness?<br>Yes (specify or choose from drop down list)/ No/ I don't know
Oculopharyngeal muscular dystrophy						
Van der Sluijs, B.M., et al. 2016<br><br>The Dutch patients' perspective on oculopharyngeal muscular dystrophy: A questionnaire study on fatigue, pain and impairments<br><br>The Netherlands	The principal aim of this study is therefore to assess the prevalence of fatigue and pain, the limitations in daily life activities and social participation, and the psychological wellbeing of OPMD patients	35 patients with OPMD, 18 men and 17 women, the average age 57.7 years	Prevalence and associations<br><br>(relation to functional impairment)	Cross-sectional study	Fatigue discussed in introduction, and defined in methods section as "an overwhelming sense of tiredness, lack of energy and feeling of exhaustion" (Vercoulen 1998, Servaes 2002).<br><br>Investigation of experienced fatigue one primary aim.<br><br>Fatigue one major focus. Fatigue mentioned 68 times	Standardized fatigue instrument:<br>the subscale fatigue severity of the Checklist Individual Strength CIS

Youssof, S., et al. 2017<br><br>Dysphagia-related quality of life in oculopharyngeal muscular dystrophy: Psychometric properties of the SWAL-QOL instrument<br><br>US	to investigate psychometric attributes of the SWAL-QOL when applied to OPMD and thereby assess its utility as an OPMD research outcome measure	113 participants with OPMD, 55 men, mean age 65.8	Diagnostics - Validation of outcome measure for a patient group<br><br>Evaluate validity and reliability of the SWAL-QOL in this patient group	prospective, cross-sectional study	Fatigue not mentioned in introduction, no definition given.<br><br>Investigation of experienced fatigue one secondary aim/ outcome.<br><br>Fatigue medium focus. Fatigue mentioned 20 times.	Standardized outcome instrument:<br>SWAL-QOL (44 items) consists of 10 scales (30 items) – including a fatigue scale.
Rare genetic respiratory disease						
Cystic fibrosis						
Bradley, J., et al. 1999<br><br>The adaptations of a quality of life questionnaire for routine use in clinical practice: the Chronic Respiratory Disease Questionnaire in cystic fibrosis<br><br>Northern Ireland	to demonstrate the methodology involved in the adaptation and shortening of the Chronic Respiratory Disease Questionnaire (CRDQ) in a population of adults with cystic fibrosis (CF).	45 adults with CF, 32 males.	Diagnostics. Validation of outcome measure for a patient group.<br><br>Psychometric testing of disease specific QoL questionnaire, fatigue included as one dimension	Test retest questionnaire study	Fatigue not mentioned in introduction, no fatigue definition described<br><br>Investigation of experienced fatigue one secondary aim/ outcome.<br><br>Fatigue medium focus. Fatigue mentioned 17 times	Standardized outcome instrument: Chronic Respiratory Disease Questionnaire (CRDQ), fatigue one of 4 dimensions.
Dancey, D.R., et al. 2002<br><br>Sleep quality and daytime function in adults with cystic fibrosis and severe lung disease<br><br>Canada	to determine whether adult CF patients with severe lung disease have impaired daytime function, and whether this is related to sleep disruption and nocturnal hypoxaemia.	19 CF patients, 15 male. Mean age 30, and 10 healthy controls.	Prevalence and associations	Cross-sectional cohort study, with polysomno-graphy	Fatigue not mentioned in introduction, no definition given<br><br>Investigation of experienced fatigue one secondary aim/ outcome.<br><br>Fatigue minor focus. Fatigue mentioned 7 times.	Standardized outcome instruments:<br>Profile of Mood States, assessing fatigue, happiness, activation, depression, anger and fear.

De Jong, W., et al. 2001<br><br>Inspiratory muscle training in patients with cystic fibrosis<br><br>The Netherlands	to evaluate the effects of inspiratory-threshold loading at low intensity in patients with CF on strength and endurance of the inspiratory muscles, pulmonary function, exercise capacity, dyspnoea and fatigue.	16 CF patients, 8 men, age 10-25	Intervention effects<br><br>changes in subjective fatigue used as one variable to assess effect of the training program	16 patients assigned to one of two groups using the minimization method: eight patients in the training group and eight patients in the control group.	Fatigue briefly mentioned in introduction, no definition given<br><br>Investigation of experienced fatigue one primary aim/ outcome.<br><br>Fatigue medium focus, fatigue mentioned 20 times	Standardized fatigue instrument: Multidimensional Fatigue Index 20 (MFI-20).
Dwyer, T.J., et al 2015<br><br>Non-invasive ventilation used as an adjunct to airway clearance treatments improves lung function during an acute exacerbation of cystic fibrosis: a randomised trial<br><br>Australia	To investigate if during an acute exacerbation of cystic fibrosis, is non-invasive ventilation beneficial as an adjunct to the airway clearance regimen?	40 adults with moderate to severe cystic fibrosis, 19 in experimental group, 21 in control. 14 female, mean age 30 years	Intervention effects<br><br>Fatigue used as on variable to evaluate if non-invasive beneficial for CF patients	Randomized controlled trial with concealed allocation and intention-to-treat analysis	Respiratory muscle fatigue is described as one feature of CF in the introduction, no definition given on experienced fatigue.<br><br>Investigation of experienced fatigue one secondary aim/ outcome.<br><br>Fatigue medium focus. Fatigue mentioned 27 times	Standardized fatigue instrument:<br>The Schwarz fatigue scale
Jamieson, N., et al. 2014<br><br>Children's experiences of cystic fibrosis: a systematic review of qualitative studies	to describe the experiences and perspectives of children and adolescents with CF to direct care toward areas that patients regard as important.	43 articles involving 729 participants aged from 4 to 21 years across 10 countries were included.	Patient experiences	Systematic review of qualitative studies	Fatigue not mentioned in introduction, no definition given,<br><br>Investigation of experienced fatigue one secondary aim/ outcome.<br>Fatigue minor focus. Fatigue mentioned 3 times	
Jarad, N.A., et al. 2012<br><br>Fatigue in cystic fibrosis: a novel prospective study investigating subjective and objective factors associated with fatigue	This study aimed at formally and prospectively examining factors associated with fatigue in a cohort of adult patients with CF during disease stability.	44 CF patients, 22 males. Men age 26.5, and 34 healthy controls	Prevalence of fatigue and associations	Prospective, case–control study	Fatigue definition thoroughly described and discussed in introduction: “Fatigue is often difficult to describe. To some patients, it represents the inability to perform day to-day activities. To others, it is a combination of tiredness, decreased muscle activity, and lower mood. Therefore, several terms are often	Standardized fatigue instrument: Chalder fatigue questionnaire

United kingdom					used to describe fatigue, such as lack of energy, tiredness, and muscle weakness”.<br><br>Investigation of experienced fatigue one primary aim.<br>Fatigue major focus. Fatigue mentioned >75 times	
Nap-van der Vlist, M., et al. 2018<br><br>Prevalence of severe fatigue among adults with cystic fibrosis: A single center study<br><br>The Netherlands	to investigate the prevalence of severe fatigue among adults with CF and to identify factors associated with fatigue.	77 CF patients (age 19–54 years; 56% males)	Prevalence of severe fatigue and factors associated with fatigue.	Questionnaire study.<br>Cross-sectional, single-center study	Fatigue definition discussed in introduction: “Fatigue is a complex phenomenon, it is likely that a multifactorial model will be needed in order to understand the cause of fatigue in these patients. Fatigue can be influenced by both biological and psychosocial factors, giving rise to the so-called biopsychosocial mode” (Knoop 2010)”.<br><br>Investigation of experienced fatigue primary aim.<br>Fatigue major focus. Fatigue mentioned >100 times	Standardized fatigue instrument: Checklist Individual Strength-20
Nap-van der Vlist, M., et al. 2019<br><br>Fatigue in childhood chronic disease<br><br>The Netherlands	to investigate: (1) the prevalence and extent of fatigue among various paediatric chronic diseases and (2) the effect of fatigue on health-related quality of life (HRQoL).	Children with CF (111, 54 boys, mean age 11.3), with autoimmune disease (211), post-cancer treatment (93) and control groups (558).	Prevalence of fatigue and factors associated with fatigue.	Cross-sectional questionnaire study	Fatigue discussed in introduction, no definition given.<br><br>Investigation of experienced fatigue primary aim.<br><br>Fatigue major focus. fatigue mentioned 165 times	Standardized fatigue instrument: Pediatric Quality of Life Inventory (PedsQL) multi-dimensional fatigue scale.
Orava, C., et al. 2018<br><br>Relationship between Physical Activity and Fatigue in Adults with Cystic Fibrosis	To examine the relationship between the amount of physical activity and level of fatigue in adults with cystic fibrosis	22 adults with cystic fibrosis (10 men, median age 33).	Prevalence and associations<br><br>Prevalence and level of fatigue and relationship between physical	Cross-sectional questionnaire study	Fatigue definition given in introduction: “In this study, we defined fatigue as a subjective lack of physical, mental, or emotional energy that an individual perceives as interfering with his or her usual and desired activities. This categorization	Standardized fatigue instrument: Multidimensional Fatigue Inventory

Canada			activity and level of fatigue		is based on the Multidimensional Fatigue Inventory (MFI) (Smets 1995).<br><br>Investigation of experienced fatigue one primary aim.<br>Fatigue major focus. Fatigue mentioned >95times.	
Wietlisbach, M.; et al. 2020<br><br>Perceptions towards physical activity in adult lung transplant recipients with cystic fibrosis<br><br>Switzerland	to explore perceptions towards physical activity (PA) in LTx recipients with CF to better understand their needs and preferences for regular PA.	111 lung transplant recipients with cystic fibrosis, 53 female, mean age 35 (29-41)	Prevalence of fatigue as perceived barrier to physical activity<br><br>And associations to fatigue	Cross-sectional online survey	Fatigue not mentioned in introduction, no definition given<br><br>Investigation of experienced fatigue one secondary aim/ outcome.<br>Fatigue minor focus. Fatigue mentioned 8 times	Study-specific questionnaire with questions on motives (i.e., factors that increase the likelihood of participating in PA) and barriers (i.e., factors that hinder participation in PA) towards PA, scored on a 0–6 Likert scale (0 = not at all relevant, 6 = highly relevant), and free text answers options.
Wolter, J.M., et al. 1997<br><br>Home intravenous therapy in cystic fibrosis: A prospective randomized trial examining clinical, quality of life and cost aspects<br><br>Australia	to determine if home intravenous antibiotic therapy in adult patients with cystic fibrosis (CF) is a feasible, effective and less costly alternative to hospitalization, and to assess the impact of home therapy on quality of life.	Seventeen CF patients, 38% male, 19–41 yrs (median 22 yrs)	Intervention effects	prospective, randomized, two-factor mixed design	Fatigue not mentioned in introduction, no definition given.<br><br>Investigation of experienced fatigue one secondary aim/ outcome.<br>Fatigue minor focus. Fatigue mentioned 5 times.	Standardized outcome instrument: The Chronic Respiratory Disease Questionnaire (CRDQ). This is a measure of change in dyspnoea, fatigue, emotion and mastery
Lymphangioleiomyelomatosis						
Bahmer, T., et al. 2016<br><br>Reduced physical activity in lymphangio-leiomyomatosis compared with COPD and healthy controls:	To study daily physical activity and clinical correlates in LAM patients.	34 female patients with LAM (mean age 52.7 years), 32 female patients with COPD (mean age 65.2 years),	Prevalence of fatigue and associations to physical activity	Cross-sectional survey	Fatigue briefly mentioned in introduction, no definition given.<br><br>Investigation of experienced fatigue one secondary aim/ outcome.	Standardized fatigue instrument: Multidimensional Fatigue Inventory, MFI-20

disease specific impact and clinical correlates<br><br>Germany		and 15 healthy women.			Fatigue minor focus. Fatigue mentioned 9 times	
Belkin, A., et al. 2014 "Getting stuck with LAM": patients perspectives on living with lymphangioleio-myomatosis<br><br>USA	To capture patients' perceptions of how LAM affects their lives.	37 women with LAM, median age 52 (range 34-68)	Views and experiences	Qualitative study	Fatigue not mentioned in introduction, no definition given.<br><br>Investigation of experienced fatigue one secondary aim/ outcome. Fatigue minor focus. Fatigue mentioned 7 times	Focus group interviews
Cohen, M.M., et al. 2009<br><br>Pregnancy experiences among women with lymphangioleio-myomatosis<br><br>USA, UK, Canada and other countries	to determine pregnancy and health outcomes in LAM to provide better evidence with which to council patients contemplating pregnancy.	328 women with LAM participated	Prevalence of fatigue	Questionnaire study	Fatigue briefly mentioned in introduction, no definition given<br><br>Investigation of experienced fatigue one secondary aim/ outcome. Fatigue minor focus. Fatigue mentioned 9 times	Study- specific questionnaire. Fatigue was assessed by the use of a 10 cm visual analogue scale (1= most fatigued/tired, 10= least fatigued/tired).
Rare genetic vascular diseases						
Ehlers-Danlos syndromes, (articles giving data on rare subtypes)						
Schubart, J. R. et al 2019 .<br>Cluster Analysis to Delineate Symptom Profiles in an Ehlers-Danlos Syndrome Patient Population.<br><br>USA	to seek evidence of phenotypic subgroups of patients with distinctive symptom profiles and describe these resulting subgroups.	175 EDS patents, at least 21 years old Vascular (51) Classical (26), Hypermobile (34), or Rare and Unclassified (64). Mean age 42 years, 77% female.	Prevalence of fatigue and association to EDS subtypes	Retrospective study. Data were extracted from a repository (registry) assembled 2001 to 2013	Fatigue briefly mentioned in introduction, no definition given.<br><br>Investigation of experienced fatigue one secondary aim/ outcome. Fatigue medium focus<br>Fatigue mentioned 38 times	Standardized fatigue instrument: Physical Fatigue (PF) and Mental Fatigue, measured by the Multidimensional Fatigue Inventory (MFI), using the PF and Mental Fatigue subscales.

Voermans, N. C. et al 2010.<br><br>Fatigue is a frequent and clinically relevant problem in Ehlers-Danlos Syndrome.<br><br>The Netherlands	To measure fatigue, its clinical relevance, and possible determinants in EDS	273 EDS patients: Vascular (11), classical (45), Kyphoscoliotic (2), hypermobile (162), unknown EDS type (53). 244 female, mean age 40.7 (16-89).	Prevalence of severe fatigue and associations (determining factors)	Cross sectional questionnaire study	Fatigue definition given in introduction: an overwhelming sense of tiredness, lack of energy, and feeling of exhaustion and is not the same as muscle weakness (Verkoulen JH 1994, Kalkman JS 2005)<br><br>Investigation of experienced fatigue one primary aim.<br>Fatigue major focus. Fatigue mentioned > 100 times	Standardized fatigue instrument: The Checklist Individual Strength (CIS)
Voermans, N.C. et al. 2011<br><br>Fatigue is associated with muscle weakness in Ehlers-Danlos syndrome: an explorative study<br><br>The Netherlands	to investigate the relationship between fatigue severity and subjective and objective measures of muscle weakness. Furthermore, the predictive value of muscle weakness for fatigue severity was determined, together with that of pain and physical activity.	Thirty EDS patients: Vascular (6), classical (11), , TNX-deficient (6), hypermobility (5), kyphoscoliotic (2). Mean age 34 (16 -36), 23 female.	Prevalence of severe fatigue and association to muscle weakness	An explorative, cross-sectional, observational study.	Fatigue mentioned in introduction, no definition given.<br><br>Investigation of experienced fatigue one primary aim/ outcome.<br><br>Fatigue major focus<br>Fatigue mentioned >70 times	Standardized fatigue instrument: The Checklist Individual Strength subscale fatigue
Marfan syndrome						
Bathen, T., et al. 2014<br><br>Fatigue in adults with Marfan syndrome, occurrence and associations to pain and other factors<br><br>Norway	to explore how fatigue affects MFS patients in their daily lives by examining the level of fatigue and prevalence of severe fatigue in adults with verified MFS.	72 persons with verified Marfan syndrome, 41 women (57%).<br><br>Mean age 44.2 (range 20-71)	Prevalence and associations	Cross-sectional questionnaire study	Fatigue definition given: it is often defined as "overwhelming sense of tiredness, lack of energy and feeling of exhaustion, mental, physical or both" [Dittner et al., 2004].<br><br>Investigation of experienced fatigue primary aim.<br>Fatigue major focus. Fatigue mentioned: > 100 times	Standardized fatigue instrument: Fatigue Severity Scale (FSS).
Benninghoven, D., et al. 2017<br><br>Inpatient rehabilitation for adult patients with Marfan syndrome: an	to confirm that our rehabilitation program was feasible and medically save. Another aim of our study was to apply standardized instruments to assess the impact of our rehabilitation program on physical fitness	18 adults with verified Marfan syndrome (17) and Loeys-Dietz syndrome (1), 14 women. Mean age 46,7 years.	Intervention effects	Observational pilot study	Fatigue definition not given.<br><br>Investigation of experienced fatigue one secondary aim/ outcome.<br>Fatigue minor focus. Fatigue mentioned 11 times	Standardized fatigue instrument: Fatigue Severity Scale (FSS).

observational pilot study<br><br>Germany	and psychological wellbeing of participants.					
Percheron, G., et al. 2007<br><br>Muscle strength and body composition in adult women with Marfan syndrome<br><br>France	to assess skeletal muscle function and body composition in a group of women with Marfan syndrome compared with matched controls.	21 women with verified Marfan syndrome , mean age 34.5 (20.9 – 53.7) and 19 healthy controls	Prevalence of fatigue	Cross-sectional study with muscle testing and questionnaires	Muscle fatigue briefly mentioned in introduction. No definition given for experienced fatigue.<br><br>Investigation of experienced fatigue one secondary aim/ outcome. Fatigue moderate focus. Fatigue mentioned 19 times.	Standardized fatigue instrument: Fatigue severity scale
Peters, K.F. et al 2001a<br><br>Living with Marfan syndrome I. Perceptions of the condition. USA	to understand the relationship between respondents' clinical features and family history and their perceptions of the condition as conceptualized by the self-regulatory model of illness perception.	174 patients with Marfan syndrome (73 male, mean age 39.8),	Prevalence of fatigue and associated factors	Cross-sectional questionnaire study	Fatigue not described in introduction or in methods.<br><br>Investigation of experienced fatigue one secondary aim/ outcome. Fatigue minor focus. Fatigue mentioned 14 times .	Standardized outcome instrument: The Illness Perception Questionnaire (IPQ), with five subscales, (36 items), Participants respond to each IPQ-identity item using a 4-point Likert scale (1, never; 4, all of the time). Fatigue is one symptom patients are asked to rate.
Peters, K.F. et al. 2001b<br><br>Living with Marfan syndrome II. Medication adherence and physical activity modification<br><br>USA<br><br>Clinical Genetics	to understand how clinical features of Marfan syndrome, specifically cardiovascular history, as well as the respondents' subjective views of the condition and its treatment would influence adherence to prescribed medication regimens and conformity with modifications of physical activity.	174 patients with Marfan syndrome (73 male, mean age 39.8), Recruited from genetics clinic and patient association (87.4%).	Associated factors (Correlation between fatigue and medication use) .	Cross-sectional questionnaire study	Fatigue not described in introduction or in methods.<br><br>Investigation of experienced fatigue one secondary aim/ outcome. Fatigue minor focus. Fatigue mentioned 7 times.	Standardized outcome instrument: The Illness Perception Questionnaire (IPQ), with five subscales, (36 items), Participants respond to each IPQ-identity item using a 4-point Likert scale (1, never; 4, all of the time). Fatigue is one symptom patients are asked to rate.

Data from the same study as reported above						
Rand-Hendriksen, S., et al. 2007<br><br>Fatigue, cognitive functioning and psychological distress in Marfan syndrome, a pilot study<br><br>Norway	To assess self-reported fatigue, cognitive function and psychological distress, correlations among them and correlations to visual acuity, joint hypermobility and use of beta-blockers	16 patients with verified Marfan syndrome, three male, mean age 24	Prevalence and associated factors	Cross sectional observational study	Fatigue briefly mentioned in introduction, no definition given.<br><br>Investigation of experienced fatigue primary aim.<br>Fatigue major focus. Fatigue mentioned 30 times.	Standardized fatigue instruments:<br><br>Fatigue Severity Scale<br>Fatigue Questionnaire
Rao, S.S., et al. 2016<br><br>Quantifying Health Status and Function in Marfan Syndrome<br><br>US	To understand the self-perception of physical and mental well-being in patients with MFS.	230 patients with verified Marfan syndrome, 97 male, mean age 44 (14-82)	Prevalence	Cross-sectional questionnaire study	Fatigue not mentioned in introduction, no definition given<br><br>Investigation of experienced fatigue one secondary aim/ outcome.<br><br>Fatigue minor focus.<br>Fatigue mentioned 8 times	Study-specific questionnaire using VAS scale 0 – 10 to ask for main problems as perceived by the patients, fatigue being one of them.<br>Fatigue perceived as a problem if patient rated higher than 6 on the VAS scale.
Van Dijk, N., et al. 2008<br><br>Is fatigue in Marfan syndrome related to orthostatic intolerance?<br><br>The Netherlands	To investigate 1, the relationship between symptoms of orthostatic tolerance and fatigue in patients with Marfan syndrome, and 2, whether termination of beta-blocker therapy improves orthostatic tolerance	59 adults with Marfan syndrome, 32 males, mean age 35 (23-68)	Prevalence of fatigue and associations (relationship to orthostatic tolerance)	Questionnaire study	Fatigue mentioned in introduction , no definition given.<br><br>Investigation of experienced fatigue one primary aim/ outcome.<br><br>Fatigue moderate focus. Fatigue mentioned 30 times	Standardized fatigue instrument:<br>Multidimensional fatigue inventory MFI-20
Velvin, G., et al. 2015<br><br>Work participation in adults with Marfan syndrome: Demographic	to investigate work participation in adults in the age of 20–67 years with verified MFS diagnosis and explore how health-related consequences of MFS and	70 adults with MFS, 40 women, mean age 43 (20-67)	Prevalence of fatigue and relationship – associations to work participation	Cross-sectional postal questionnaire study	Fatigue briefly mentioned in introduction, no definition given<br><br>Investigation of experienced fatigue one secondary aim/ outcome.	Standardized fatigue instrument: Fatigue Severity Scale (FSS).<br>FSS score of ≤4 indicated no fatigue, and a mean FSS

characteristics, MFS related health symptoms, chronic pain, and fatigue<br><br>Norway	other factors influence work participation.				Fatigue moderate focus. Fatigue mentioned 54 times	score of ≥5 indicated severe fatigue
Velvin, G. et al 2016<br><br>Satisfaction with life in adults with Marfan syndrome (MFS): associations with health-related consequences of MFS, pain, fatigue, and demographic factors.<br><br>Norway	to explore satisfaction with life (SWL) among adults with Marfan syndrome (MFS) compared to the general Norwegian population and other patient groups and further to examine the associations between SWL and demographic factors, contact with social and health services, MFS-related health problems, chronic pain, and fatigue.	73 adults with Marfan syndrome, 57% women, mean age 44.2.	Prevalence of fatigue and associations (relationship- to satisfaction with life)	Cross-sectional postal questionnaire study	Fatigue briefly mentioned in introduction, no definition given<br><br>Investigation of experienced fatigue one secondary aim/ outcome. Fatigue moderate focus. Fatigue mentioned 28 times	Standardized fatigue instrument: Fatigue Severity Scale (FSS).
Warnink-Kavelaars, J. et al 2019<br>Marfan syndrome in childhood: parents' perspectives of the impact on daily functioning of children, parents and family; a qualitative study.<br><br>The Netherlands	The aim of this qualitative study was to explore parents' perspectives on the impact of MFS on daily functioning of children with MFS aged 4-12 years, themselves and family regarding functional performance, activities, participation, personal and environmental factors, and disease burden.	N= 10 in individual interviews, and 3 focus groups (n = 5, n = 5 and n = 6). All participants were parents of a child with MFS aged 4–12 years.	Views and experiences Parents views experiences of how the child with MFS perceive their condition. Fatigue one limiting symptom	Qualitative study	Fatigue briefly mentioned in introduction, no definition given,<br><br>Investigation of experienced fatigue one secondary aim/ outcome. Fatigue minor focus. Fatigue mentioned 6 times	Semi-structured individual interviews and focus group interviews.
