# Supplementary material for: Experienced fatigue in people with rare disorders: a scoping review on characteristics of existing research
Source: Orphanet J Rare Dis. 2022 Jan 10;17:14. doi: 10.1186/s13023-021-02169-6 (PMC8751355; doi:10.1186/s13023-021-02169-6)
Supplement: Supplementary file 4 — Additional file 4. Reference list of included articles.pdf. First, a list of all included articles is given, then references from some specific themes (secondary research articles – reviews, articles on diagnostics – development/ validation of fatigue assessment tools, articles on treatment effects, articles on patient’s views and experiences). [file 13023_2021_2169_MOESM4_ESM.pdf]

Additional file 4. Reference list of included articles

First, a list of all included articles is given, then references from some specific themes (secondary research articles – reviews, articles on diagnostics – development/ validation of fatigue assessment tools, articles on treatment effects, articles on patient's views and experiences).

References all included articles, in alphabetical order

Ahlström G, Gunnarsson LG, Kihlgren A, Arvill A, Sjöden PO. Respiratory function, electrocardiography and quality of life in individuals with muscular dystrophy. Chest. 1994;106(1):173-9.

Akintoye SO, Kelly MH, Brillante B, Cherman N, Turner S, Butman JA, et al. Pegvisomant for the treatment of gsp-mediated growth hormone excess in patients with McCune-Albright syndrome. Journal of Clinical Endocrinology & Metabolism. 2006;91(8):2960-6.

Alemdaroglu I, Karaduman AA, Yilmaz O. Acute effects of different exercises on hemodynamic responses and fatigue in Duchenne muscular dystrophy. Fizyoterapi Rehabilitasyon. 2012;23(1):10-6.

Alemdaroglu-Gurbuz I, Bulut N, Bozgeyik S, Ulug N, Arslan SS, Yilmaz O, et al. Reliability and validity of the turkish translation of pedsqlTM multidimensional Fatigue scale in Duchenne Muscular Dystrophy. Neurosciences. 2019;24(4):302-10.

Alschuler KN, Jensen MP, Goetz MC, Smith AE, Verrall AM, Molton IR. Effects of pain and fatigue on physical functioning and depression in persons with muscular dystrophy. Disability & Health Journal. 2012;5(4):277-83.

Andersen G, Heje K, Buch AE, Vissing J. High-intensity interval training in facioscapulohumeral muscular dystrophy type 1: a randomized clinical trial. J Neurol. 2017;264(6):1099-106.

Anens E, Emtner M, Hellstrom K. Exploratory study of physical activity in persons with Charcot-Marie-Tooth disease. Archives of Physical Medicine & Rehabilitation. 2015;96(2):260-8.

Arponen H, Waltimo-Siren J, Valta H, Makitie O. Fatigue and disturbances of sleep in patients with osteogenesis imperfecta - a cross-sectional questionnaire study. BMC Musculoskeletal Disorders. 2018;19(1):3.

Bahmer T, Watz H, Waschki B, Gramm M, Magnussen H, Rabe KF, et al. Reduced physical activity in lymphangioleiomyomatosis compared with COPD and healthy controls: disease-specific impact and clinical correlates. Thorax. 2016;71(7):662-3.

Baldanzi S, Ricci G, Bottari M, Chico L, Simoncini C, Siciliano G. The proposal of a clinical protocol to assess central and peripheral fatigue in myotonic dystrophy type 1. Archives Italiennes de Biologie. 2017;155(1):43-53.

Ballard LM, Jenkinson E, Byrne CD, Child JC, Davies JH, Inskip H, et al. Lived experience of Silver-Russell syndrome: implications for management during childhood and into adulthood. Archives of Disease in Childhood. 2018;104(1):76-82.

Bankole LC, Millet GY, Temesi J, Bachasson D, Ravelojaona M, Wuyam B, et al. Safety and efficacy of a 6-month home-based exercise program in patients with facioscapulohumeral muscular dystrophy: A randomized controlled trial. Medicine. 2016;95(31):e4497.

Bates MG, Newman JH, Jakovljevic DG, Hollingsworth KG, Alston CL, Zalewski P, et al. Defining cardiac adaptations and safety of endurance training in patients with m.3243A>G-related mitochondrial disease. International Journal of Cardiology. 2013;168(4):3599-608.

Bathen T, Fredwall S, Steen U, Svendby EB. Fatigue and pain in children and adults with multiple osteochondromas in Norway, a cross-sectional study. International Journal of Orthopaedic and Trauma Nursing. 2019;34:28-35.

Bathen T, Velvin G, -Hendriksen S, Robinson HS. Fatigue in adults with Marfan syndrome, occurrence and associations to pain and other factors. American Journal of Medical Genetics Part A. 2014;164(8):1931-9.

Belkin A, Albright K, Fier K, Desserich J, Swigris JJ. "Getting stuck with LAM": patients perspectives on living with lymphangioleiomyomatosis. Health & Quality of Life Outcomes. 2014;12:79.

Benninghoven D, Hamann D, von Kodolitsch Y, Rybczynski M, Lechinger J, Schroeder F, et al. Inpatient rehabilitation for adult patients with Marfan syndrome: an observational pilot study. Orphanet Journal Of Rare Diseases. 2017;12(1):127.

Berntsson SG, Gauffin H, Melberg A, Holtz A, tblom AM. Inherited Ataxia and Intrathecal Baclofen for the Treatment of Spasticity and Painful Spasms. Stereotactic & Functional Neurosurgery. 2019;97(1):18-23.

Boentert M, Dziewas R, Heidbreder A, Happe S, Kleffner I, Evers S, et al. Fatigue, reduced sleep quality and restless legs syndrome in Charcot-Marie-Tooth disease: a web-based survey. Journal of Neurology. 2010;257(4):646-52.

Bogart KR, Dermody SS. Relationship of rare disorder latent clusters to anxiety and depression symptoms. Health Psychology. 2020;39(4):307-15.

Bogart KR, Irvin VL. Health-related quality of life among adults with diverse rare disorders. Orphanet journal of rare diseases. 2017;12(1):1-9.

Bossie HM, Willingham TB, Schoick RAV, O'Connor PJ, McCully KK. Mitochondrial capacity, muscle endurance, and low energy in friedreich ataxia. Muscle & Nerve. 2017;56(4):773-9.

Boström K, Ahlström G. Living with a chronic deteriorating disease: the trajectory with muscular dystrophy over ten years. Disability & Rehabilitation. 2004;26(23):1388-98.

Bradley J, Dempster M, Wallace E, Elborn S. The adaptations of a quality of life questionnaire for routine use in clinical practice: the Chronic Respiratory Disease Questionnaire in cystic fibrosis. Quality of Life Research. 1999;8(1):65-71.

Bronisch O, Stauch T, Haverkamp T, Beykirch MK, Petrides PE. Acute porphyrias: a German monocentric study of the biochemical, molecular genetic, and clinical data of 62 families. Annals of Hematology. 2019;98(12):2683-91.

Brusse E, Brusse-Keizer MGJ, Duivenvoorden HJ, van Swieten JC. Fatigue in spinocerebellar ataxia Patient self-assessment of an early and disabling symptom. Neurology. 2011;76(11):953-9.

Cohen MM, Freyer AM, Johnson SR. Pregnancy experiences among women with lymphangioleiomyomatosis. Respiratory Medicine. 2009;103(5):766-72.

Colson SS, Benchortane M, Tanant V, Faghan JP, Fournier-Mehouas M, Benaim C, et al. Neuromuscular electrical stimulation training: a safe and effective treatment for facioscapulohumeral muscular dystrophy patients. Archives of Physical Medicine & Rehabilitation. 2010;91(5):697-702.

Cook KF, Bamer AM, Amtmann D, Molton IR, Jensen MP. Six patient-reported outcome measurement information system short form measures have negligible age- or diagnosis-related differential item functioning in individuals with disabilities. Archives of Physical Medicine & Rehabilitation. 2012;93(7):1289-91.

Crescimanno G, Greco F, Abbate A, Canino M, Bertini M, Marrone O. Subjective sleep quality in adult patients affected by Duchenne muscular dystrophy. Beyond nocturnal hypoventilation. Sleep Medicine. 2020;69:168-71.

Crescimanno G, Greco F, D'Alia R, Messina L, Marrone O. Quality of life in long term ventilated adult patients with Duchenne muscular dystrophy. Neuromuscular Disorders. 2019;29(8):569-75.

Croonen EA, Harmsen M, Van der Burgt I, Draaisma JM, Noordam K, Essink M, et al. Perceived motor problems in daily life: Focus group interviews with people with Noonan syndrome and their relatives. American Journal of Medical Genetics Part A. 2016;170(9):2349-56.

Dai S, Dieterich K, Jaeger M, Wuyam B, Jouk PS, Perennou D. Disability in adults with arthrogryposis is severe, partly invisible, and varies by genotype. Neurology. 2018;90(18):e1596-e604.

Dancey DR, Tullis ED, Heslegrave R, Thornley K, Hanly PJ. Sleep quality and daytime function in adults with cystic fibrosis and severe lung disease. European Respiratory Journal. 2002;19(3):504-10.

de Jong W, van Aalderen WM, Kraan J, Koeter GH, van der Schans CP. Inspiratory muscle training in patients with cystic fibrosis. Respiratory Medicine. 2001;95(1):31-6.

Duruoz MT, Unal C, Bingul DK, Ulutatar F. Fatigue in familial Mediterranean fever and its relations with other clinical parameters. Rheumatology International. 2018;38(1):75-81.

Dwyer TJ, Robbins L, Kelly P, Piper AJ, Bell SC, Bye PT. Non-invasive ventilation used as an adjunct to airway clearance treatments improves lung function during an acute exacerbation of cystic fibrosis: a randomised trial. Journal of Physiotherapy. 2015;61(3):142-7.

El-Aloul B, Speechley KN, Wei Y, Wilk P, Campbell C. Fatigue in young people with Duchenne muscular dystrophy. Developmental Medicine & Child Neurology. 2020;62(2):245-51.

ElMhandi L, Millet GY, Calmels P, Richard A, Oullion R, Gautheron V, et al. Benefits of interval-training on fatigue and functional capacities in Charcot-Marie-Tooth disease. Muscle & Nerve. 2008;37(5):601-10.

Epstein E, Farmer JM, Tsou A, Perlman S, Subramony SH, Gomez CM, et al. Health related quality of life measures in Friedreich Ataxia. Journal of the Neurological Sciences. 2008;272(1):123-8.

Favejee MM, van den Berg LE, Kruijshaar ME, Wens SC, Praet SF, Pim Pijnappel WW, et al. Exercise training in adults with Pompe disease: the effects on pain, fatigue, and functioning. Archives of Physical Medicine & Rehabilitation. 2015;96(5):817-22.

Ferizovic N, Marshall J, Williams AE, Mughal MZ, Shaw N, Mak C, et al. Exploring the Burden of X-Linked Hypophosphataemia: An Opportunistic Qualitative Study of Patient Statements Generated During a Technology Appraisal. Advances in Therapy. 2020;37(2):770-84.

Forsyth A, Gregory M, Nugent D, Garrido C, Pilgaard T, Cooper D, et al. Haemophilia Experiences, Results and Opportunities (HERO) Study: survey methodology and population demographics. Haemophilia. 2014;20(1):44-51.

Fujino H, Shingaki H, Suwazono S, Ueda Y, Wada C, Nakayama T, et al. Cognitive impairment and quality of life in patients with myotonic dystrophy type 1. Muscle & Nerve. 2018;57(5):742-8.

Gagnon C, Mathieu J, Jean S, Laberge L, Perron M, Veillette S, et al. Predictors of disrupted social participation in myotonic dystrophy type 1. Archives of Physical Medicine & Rehabilitation. 2008;89(7):1246-55.

Gallais B, Gagnon C, Forgues G, Cote I, Laberge L. Further evidence for the reliability and validity of the Fatigue and Daytime Sleepiness Scale. Journal of the Neurological Sciences. 2017;375:23-6.

Gallais B, Montreuil M, Gargiulo M, Eymard B, Gagnon C, Laberge L. Prevalence and correlates of apathy in myotonic dystrophy type 1. BMC Neurology. 2015;15:148.

Gelrud A, Williams KR, Hsieh A, Gwosdow AR, Gilstrap A, Brown A. The burden of familial chylomicronemia syndrome from the patients' perspective. Expert Review of Cardiovascular Therapy. 2017;15(11):879-87.

Gliem C, Minnerop M, Roeske S, Gartner H, Schoene-Bake JC, Adler S, et al. Tracking the brain in myotonic dystrophies: A 5-year longitudinal follow-up study. PLoS ONE [Electronic Resource]. 2019;14(3):e0213381.

Gonzalez-Perez P, Smith C, Sebetka WL, Gedlinske A, Perlman S, Mathews KD. Clinical and electrophysiological evaluation of myasthenic features in an alpha-dystroglycanopathy cohort (FKRP-predominant). Neuromuscular Disorders. 2020;25(3):25.

Gorman GS, Elson JL, Newman J, Payne B, McFarl, R, et al. Perceived fatigue is highly prevalent and debilitating in patients with mitochondrial disease. Neuromuscular Disorders. 2015;25(7):563-6.

Goselink RJM, Schreuder THA, van Alfen N, de Groot IJM, Jansen M, Lemmers R, et al. Facioscapulohumeral Dystrophy in Childhood: A Nationwide Natural History Study. Annals of Neurology. 2018;84(5):627-37.

Guffon N, Fouilhoux A. Clinical benefit in Fabry patients given enzyme replacement therapy--a case series. Journal of Inherited Metabolic Disease. 2004;27(2):221-7.

Gungor D, de Vries JM, Brusse E, Kruijshaar ME, Hop WC, Murawska M, et al. Enzyme replacement therapy and fatigue in adults with Pompe disease. Molecular Genetics & Metabolism. 2013;109(2):174-8.

Hagemans ML, van Schie SP, Janssens AC, van Doorn PA, Reuser AJ, van der Ploeg AT. Fatigue: an important feature of late-onset Pompe disease. Journal of Neurology. 2007;254(7):941-5.

Hagemans ML, Winkel LP, Van Doorn PA, Hop WJ, Loonen MC, Reuser AJ, et al. Clinical manifestation and natural course of late-onset Pompe's disease in 54 Dutch patients. Brain. 2005;128(Pt 3):671-7.

Hagerman KA, Howe SJ, Heatwole CR. The myotonic dystrophy experience: a North American cross-sectional study. Muscle and Nerve. 2019;59(4):457-64.

Hajjar J, Guffey D, Minard CG, Orange JS. Increased Incidence of Fatigue in Patients with Primary Immunodeficiency Disorders: Prevalence and Associations Within the US Immunodeficiency Network Registry. Journal of Clinical Immunology. 2017;37(2):153-65.

Haller C, Song W, Cimms T, Chen CY, Whitley CB, Wang RY, et al. Individual heat map assessments demonstrate vestronidase alfa treatment response in a highly heterogeneous mucopolysaccharidosis VII study population. Jimd Reports. 2019;49(1):53-62.

Hamed A, Curran C, Gwaltney C, DasMahapatra P. Mobility assessment using wearable technology in patients with late-onset Pompe disease. Npj Digital Medicine. 2019;2:70.

Hamel J, Johnson N, Tawil R, Martens WB, Dilek N, McDermott MP, et al. Patient-Reported Symptoms in Facioscapulohumeral Muscular Dystrophy (PRISM-FSHD). Neurology. 2019;93(12):e1180-e92.

Harmatz P, Whitley CB, Wang RY, Bauer M, Song W, Haller C, et al. A novel Blind Start study design to investigate vestronidase alfa for mucopolysaccharidosis VII, an ultra-rare genetic disease. Molecular Genetics & Metabolism. 2018;123(4):488-94.

Harsevoort AGJ, Gooijer K, van Dijk FS, van der Grijn D, Franken AAM, Dommisse AMV, et al. Fatigue in adults with Osteogenesis Imperfecta. BMC Musculoskeletal Disorders. 2020;21(1):6.

Hayes R, Grinzaid K, Duffey E, Elsas L. The impact of Gaucher disease and its treatment on quality of life. Quality of Life Research. 1998;7(6):521-34.

Heatwole C, Bode R, Johnson N, Quinn C, Martens W, McDermott MP, et al. Patient-reported impact of symptoms in myotonic dystrophy type 1 (PRISM-1). Neurology. 2012;79(4):348-57.

Heatwole C, Johnson N, Bode R, Dekdebrun J, Dilek N, Hilbert JE, et al. Patient-Reported Impact of Symptoms in Myotonic Dystrophy Type 2 (PRISM-2). Neurology. 2015;85(24):2136-46.

Hendriksz CJ, Lavery C, Coker M, Ucar SK, Jain M, Bell L, et al. Burden of disease in patients with Morquio A syndrome: results from an international patient-reported outcomes survey. Orphanet Journal Of Rare Diseases. 2014;9:32.

Hermans MC, Merkies IS, Laberge L, Blom EW, Tennant A, Faber CG. Fatigue and daytime sleepiness scale in myotonic dystrophy type 1. Muscle & Nerve. 2013;47(1):89-95.

Hillmen P, Young NS, Schubert J, Brodsky RA, Socié G, Muus P, et al. The complement inhibitor eculizumab in paroxysmal nocturnal hemoglobinuria. New England Journal of Medicine. 2006;355(12):1233-43.

Ivleva A, Weith E, Mehta A, Hughes DA. The Influence of Patient-Reported Joint Manifestations on Quality of Life in Fabry Patients. Jimd Reports. 2018;41:37-45.

Jacques MF, Stockley RC, Onambele-Pearson GL, Reeves ND, Stebbings GK, Dawson EA, et al. Quality of life in adults with muscular dystrophy. Health & Quality of Life Outcomes. 2019;17(1):121.

Jagersma E, Jeukens-Visser M, van Paassen BW, Meester-Delver A, Nollet F. Severe fatigue and reduced quality of life in children with hereditary motor and sensory neuropathy 1A. Journal of Child Neurology. 2013;28(4):429-34.

Jamieson N, Fitzgerald D, Singh-Grewal D, Hanson CS, Craig JC, Tong A. Children's experiences of cystic fibrosis: a systematic review of qualitative studies. Pediatrics. 2014;133(6):e1683-97.

Jarad NA, Sequeiros IM, Patel P, Bristow K, Sund Z. Fatigue in cystic fibrosis: a novel prospective study investigating subjective and objective factors associated with fatigue. Chronic Respiratory Disease. 2012;9(4):241-9.

Johansen H, Bathen T, Andersen LO, -Hendriksen S, Ostlie K. Education and work participation among adults with congenital unilateral upper limb deficiency in Norway: A cross-sectional study. PLoS ONE [Electronic Resource]. 2018;13(12):e0207846.

Johansen H, Bathen T, Andersen LO, -Hendriksen S, Ostlie K. Chronic pain and fatigue in adults with congenital unilateral upper limb deficiency in Norway. A cross-sectional study. PLoS ONE [Electronic Resource]. 2018;13(1):e0190567.

Johnson NE, Ekstrom AB, Campbell C, Hung M, Adams HR, Chen W, et al. Parent-reported multi-national study of the impact of congenital and childhood onset myotonic dystrophy. Developmental Medicine & Child Neurology. 2016;58(7):698-705.

Johnson NE, Heatwole CR, Dilek N, Sowden J, Kirk CA, Shereff D, et al. Quality-of-life in Charcot-Marie-Tooth disease: the patient's perspective. Neuromuscular Disorders. 2014;24(11):1018-23.

Johnson NE, Quinn C, Eastwood E, Tawil R, Heatwole CR. Patient-identified disease burden in facioscapulohumeral muscular dystrophy. Muscle & Nerve. 2012;46(6):951-3.

Johnston BC, Miller PA, Agarwal A, Mulla S, Khokhar R, De Oliveira K, et al. Limited responsiveness related to the minimal important difference of patient-reported outcomes in rare diseases. Journal of Clinical Epidemiology. 2016;79:10-21.

Kalkman JS, Schillings ML, van der Werf SP, Padberg GW, Zwarts MJ, van Engelen BG, et al. Experienced fatigue in facioscapulohumeral dystrophy, myotonic dystrophy, and HMSN-I. Journal of Neurology, Neurosurgery & Psychiatry. 2005;76(10):1406-9.

Kalkman JS, Schillings ML, Zwarts MJ, van Engelen BG, Bleijenberg G. Influence of relatives on fatigue experienced by patients with facioscapulohumeral dystrophy, myotonic dystrophy and HMSN-I. European Neurology. 2006;56(1):24-30.

Kalkman JS, Schillings ML, Zwarts MJ, van Engelen BG, Bleijenberg G. The development of a model of fatigue in neuromuscular disorders: a longitudinal study. Journal of Psychosomatic Research. 2007;62(5):571-9.

Kalkman JS, Schillings ML, Zwarts MJ, van Engelen BG, Bleijenberg G. Psychiatric disorders appear equally in patients with myotonic dystrophy, facioscapulohumeral dystrophy, and hereditary motor and sensory neuropathy type I. Acta Neurologica Scandinavica. 2007;115(4):265-70.

Kalkman JS, Zwarts MJ, Schillings ML, van Engelen BG, Bleijenberg G. Different types of fatigue in patients with facioscapulohumeral dystrophy, myotonic dystrophy and HMSN-I. Experienced fatigue and physiological fatigue. Neurological Sciences. 2008;29:S238-40.

Kanters TA, Redekop WK, Rutten-Van Molken MP, Kruijshaar ME, Gungor D, van der Ploeg AT, et al. A conceptual disease model for adult Pompe disease. Orphanet Journal Of Rare Diseases. 2015;10:112.

Karaa A, Haas R, Goldstein A, Vockley J, Cohen BH. A randomized crossover trial of elamipretide in adults with primary mitochondrial myopathy. Journal of Cachexia, Sarcopenia and Muscle. 2020;25:25.

Kerstens H, Satink T, Nijkrake MJ, De Swart BJM, Van Lith BJH, Geurts ACH, et al. Stumbling, struggling, and shame due to spasticity: a qualitative study of adult persons with hereditary spastic paraplegia. Disability & Rehabilitation. 2019:1-8.

Kierkegaard M, Harms-Ringdahl K, Holmqvist LW, Tollback A. Functioning and disability in adults with myotonic dystrophy type 1. Disability & Rehabilitation. 2011;33(19):1826-36.

Knak KL, Sheikh AM, Witting N, Vissing J. Physical activity in myotonic dystrophy type 1. Journal of Neurology. 2020;17(6):17.

Koene S, Wortmann SB, de Vries MC, Jonckheere AI, Morava E, de Groot IJ, et al. Developing outcome measures for pediatric mitochondrial disorders: which complaints and limitations are most burdensome to patients and their parents? Mitochondrion. 2013;13(1):15-24.

Koopman FS, Brehm MA, Beelen A, Voet N, Bleijenberg G, Geurts A, et al. Cognitive behavioural therapy for reducing fatigue in post-polio syndrome and in facioscapulohumeral dystrophy: A comparison. Journal of Rehabilitation Medicine. 2017;49(7):585-90.

Kuo A, Todd JJ, Witherspoon JW, Lawal TA, Elliott J, Chrismer IC, et al. Reliability and Validity of Self-Report Questionnaires as Indicators of Fatigue in RYR1-Related Disorders. Journal of neuromuscular diseases. 2019;6(1):133-41.

Laberge L, Dauvilliers Y, Begin P, Richer L, Jean S, Mathieu J. Fatigue and daytime sleepiness in patients with myotonic dystrophy type 1: to lump or split? Neuromuscular Disorders. 2009;19(6):397-402.

Laberge L, Gagnon C, Jean S, Mathieu J. Fatigue and daytime sleepiness rating scales in myotonic dystrophy: a study of reliability. Journal of Neurology, Neurosurgery & Psychiatry. 2005;76(10):1403-5.

Laberge L, Gallais B, Auclair J, Dauvilliers Y, Mathieu J, Gagnon C. Predicting daytime sleepiness and fatigue: a 9-year prospective study in myotonic dystrophy type 1. Journal of Neurology. 2020;267(2):461-8.

Laberge L, Mathieu J, Auclair J, Gagnon E, Noreau L, Gagnon C. Clinical, psychosocial, and central correlates of quality of life in myotonic dystrophy type 1 patients. European Neurology. 2013;70(5):308-15.

Lai JS, Jensen SE, Charrow J, Listernick R. Patient Reported Outcomes Measurement Information System and Quality of Life in Neurological Disorders Measurement System to Evaluate Quality of Life for Children and Adolescents with Neurofibromatosis Type 1 Associated Plexiform Neurofibroma. Journal of Pediatrics. 2019;206:190-6.

Lam EM, Shepard PW, St Louis EK, Dueffert LG, Slocumb N, McCarter SJ, et al. Restless legs syndrome and daytime sleepiness are prominent in myotonic dystrophy type 2. Neurology. 2013;81(2):157-64.

Landfeldt E, Nikolenko N, Jimenez-Moreno C, Cumming S, Monckton DG, Gorman G, et al. Disease burden of myotonic dystrophy type 1. Journal of Neurology. 2019;266(4):998-1006.

Lecordier D, Cartron E, Jovic L. Understanding people with Steinert's disease to better care for them. Recherche En Soins Infirmiers. 2017(131):13-28.

Lindsay S, Cagliostro E, McAdam L. Meaningful occupations of young adults with muscular dystrophy and other neuromuscular disorders. Canadian Journal of Occupational Therapy - Revue Canadienne d Ergotherapie. 2019;86(4):277-88.

Lo SH, Lachmann R, Williams A, Piglowska N, Lloyd AJ. Exploring the burden of X-linked hypophosphatemia: a European multi-country qualitative study. Quality of Life Research. 2020;29(7):1883–93.

Looman WS, Thurmes AK, O'Conner-Von SK. Quality of life among children with velocardiofacial syndrome. Cleft Palate-Craniofacial Journal. 2010;47(3):273-83.

MacDonald JR, Hill JD, Tarnopolsky MA. Modafinil reduces excessive somnolence and enhances mood in patients with myotonic dystrophy. Neurology. 2002;59(12):1876-80.

Magerl M, Doumoulakis G, Kalkounou I, Weller K, Church MK, Kreuz W, et al. Characterization of prodromal symptoms in a large population of patients with hereditary angio-oedema. Clinical & Experimental Dermatology. 2014;39(3):298-303.

Martens AM, Gorter H, Wassink RG, Rietman H. Physical activity of children with a mitochondrial disease compared to children who are healthy. Pediatric Physical Therapy. 2014;26(1):19-26.

Martí-Carvajal AJ, An, V, Cardona AF, Solà I. Eculizumab for treating patients with paroxysmal nocturnal hemoglobinuria. Cochrane Database of Systematic Reviews. 2014(10):CD010340.

Mazar I, Stokes J, Ollis S, Love E, Espensen A, Barth PG, et al. Understanding the life experience of Barth syndrome from the perspective of adults: a qualitative one-on-one interview study. Orphanet Journal Of Rare Diseases. 2019;14(1):243.

Meilleur KG, Jain MS, Hynan LS, Shieh CY, Kim E, Waite M, et al. Results of a two-year pilot study of clinical outcome measures in collagen VI- and laminin alpha2-related congenital muscular dystrophies. Neuromuscular Disorders. 2015;25(1):43-54.

Messina S, Vita GL, Sframeli M, Mondello S, Mazzone E, D'Amico A, et al. Health-related quality of life and functional changes in DMD: A 12-month longitudinal cohort study. Neuromuscular Disorders. 2016;26(3):189-96.

Micallef J, Attarian S, Dubourg O, Gonnaud P-M, Hogrel J-Y, Stojkovic T, et al. Effect of ascorbic acid in patients with Charcot–Marie–Tooth disease type 1A: a multicentre, randomised, double-blind, placebo-controlled trial. The Lancet Neurology. 2009;8(12):1103-10.

Minis MA, Kalkman JS, Akkermans RP, Engels JA, Huijbregts PA, Bleijenberg G, et al. Employment status of patients with neuromuscular diseases in relation to personal factors, fatigue and health status: a secondary analysis. Journal of Rehabilitation Medicine. 2010;42(1):60-5.

Minis MAH, Satink T, Kinebanian A, Engels JA, Heerkens YF, van Engelen BGM, et al. How Persons with a Neuromuscular Disease Perceive Employment Participation: A Qualitative Study. Journal of Occupational Rehabilitation. 2014;24(1):52-67.

Minnerop M, Weber B, Schoene-Bake JC, Roeske S, Mirbach S, Anspach C, et al. The brain in myotonic dystrophy 1 and 2: evidence for a predominant white matter disease. Brain. 2011;134(Pt 12):3530-46.

Murray CB, Palermo TM, Holmbeck GN. A Multimethod, Case-Controlled Study of Sleep-Wake Disturbances in Adolescents With Spina Bifida. Journal of Pediatric Psychology. 2018;43(6):601-12.

Naik H, Overbey JR, Montgomery GH, Winkel G, Balwani M, Anderson KE, et al. Evaluating the Patient-Reported Outcomes Measurement Information System scales in acute intermittent porphyria. Genetics in Medicine. 2020;22(3):590-7.

Naik H, Stoecker M, erson SC, Balwani M, Desnick RJ. Experiences and concerns of patients with recurrent attacks of acute hepatic porphyria: A qualitative study. Molecular Genetics & Metabolism. 2016;119(3):278-83.

Nap-van der Vlist MM, Burghard M, Hulzebos HJ, Doeleman WR, Heijerman HGM, van der Ent CK, et al. Prevalence of severe fatigue among adults with cystic fibrosis: A single center study. Journal of Cystic Fibrosis. 2018;17(3):368-74.

Nap-van der Vlist MM, Dalmeijer GW, Grootenhuis MA, van der Ent CK, van den Heuvel-Eibrink MM, Wulffraat NM, et al. Fatigue in childhood chronic disease. Archives of Disease in Childhood. 2019;104(11):1090-5.

Nielsen J, Pelsen B, Sorensen K. Follow-up of 30 Klinefelter males treated with testosterone. Clinical Genetics. 1988;33(4):262-9.

O'Donoghue FJ, Borel JC, Dauvilliers Y, Levy P, Tamisier R, Pepin JL. Effects of 1-month withdrawal of ventilatory support in hypercapnic myotonic dystrophy type 1. Respirology. 2017;22(7):1416-22.

Okkersen K, Jimenez-Moreno C, Wenninger S, Daidj F, Glennon J, Cumming S, et al. Cognitive behavioural therapy with optional graded exercise therapy in patients with severe fatigue with myotonic dystrophy type 1: a multicentre, single-blind, randomised trial. Lancet Neurology. 2018;17(8):671-80.

Oladapo AO, Ito D, Hibbard C, Bean SE, Krupnick RN, Ewenstein BM. Patient Experience with Congenital (Hereditary) Thrombotic Thrombocytopenic Purpura: A Conceptual Framework of Symptoms and Impacts. The Patient: Patient-Centered Outcomes Research. 2019;12(5):503-12.

Orava C, Fitzgerald J, Figliomeni S, Lam D, Naccarato A, Szego E, et al. Relationship between Physical Activity and Fatigue in Adults with Cystic Fibrosis. Physiotherapy Canada. 2018;70(1):42-8.

Pangalila RF, van den Bos GA, Bartels B, Bergen M, Stam HJ, Roebroeck ME. Prevalence of fatigue, pain, and affective disorders in adults with duchenne muscular dystrophy and their

associations with quality of life. Archives of Physical Medicine & Rehabilitation. 2015;96(7):1242-7.

Pareyson D, Reilly MM, Schenone A, Fabrizi GM, Cavallaro T, Manganelli L, et al. Ascorbic acid in charcot-marie-tooth disease type 1A (CMTTRIAAL and CMT-TRAUK): a double-blind randomised trial. Lancet neurology. 2011;10(4):320-8.

Parikh S, Galioto R, Lapin B, Haas R, Hirano M, Koenig MK, et al. Fatigue in primary genetic mitochondrial disease: No rest for the weary. Neuromuscular Disorders. 2019;29(11):895-902.

Paulsen EK, Friedman LS, Myers LM, Lynch DR. Health-related quality of life in children with Friedreich ataxia. Pediatric Neurology. 2010;42(5):335-7.

Percheron G, Fayet G, Ningler T, Le Parc JM, Denot-Ledunois S, Leroy M, et al. Muscle strength and body composition in adult women with Marfan syndrome. Rheumatology. 2007;46(6):957-62.

Peric M, Peric S, Stevanovic J, Milovanovic S, Basta I, Nikolic A, et al. Quality of life in adult patients with limb-girdle muscular dystrophies. Acta Neurologica Belgica. 2018;118(2):243-50.

Peric S, Bjelica B, Bozovic I, Pesovic J, Paunic T, Banovic M, et al. Fatigue in myotonic dystrophy type 1: a seven-year prospective study. Acta Myologica. 2019;38(4):239-44.

Peric S, Pavlovic A, Ralic V, Dobricic V, Basta I, Lavrnic D, et al. Transcranial sonography in patients with myotonic dystrophy type 1. Muscle & Nerve. 2014;50(2):278-82.

Peric S, Stojanovic VR, Basta I, Peric M, Milicev M, Pavlovic S, et al. Influence of multisystemic affection on health-related quality of life in patients with myotonic dystrophy type 1. Clinical Neurology & Neurosurgery. 2013;115(3):270-5.

Peters KF, Horne R, Kong F, Francomano CA, Biesecker BB. Living with Marfan syndrome II. Medication adherence and physical activity modification. Clinical Genetics. 2001;60(4):283-92.

Peters KF, Kong F, Horne R, Francomano CA, Biesecker BB. Living with Marfan syndrome I. Perceptions of the condition. Clinical Genetics. 2001;60(4):273-82.

Pickard AS, Huynh L, Ivanova JI, Totev T, Graham S, Muhlbacher AC, et al. Value of transfusion independence in severe aplastic anemia from patients' perspectives - a discrete choice experiment. Journal of Patientreported Outcomes. 2017;2(1):13.

Pincherle A, Patruno V, Raimondi P, Moretti S, Dominese A, Martinelli-Boneschi F, et al. Sleep breathing disorders in 40 Italian patients with Myotonic dystrophy type 1. Neuromuscular Disorders. 2012;22(3):219-24.

Quera Salva MA, Blumen M, Jacquette A, Dur, MC, Andre S, et al. Sleep disorders in childhood-onset myotonic dystrophy type 1. Neuromuscular Disorders. 2006;16(9):564-70.

Rakocevic Stojanovic V, Peric S, Paunic T, Pesovic J, Vujnic M, Peric M, et al. Quality of life in patients with myotonic dystrophy type 2. Journal of the Neurological Sciences. 2016;365:158-61.

Rakocevic-Stojanovic V, Peric S, Madzarevic R, Dobricic V, Ralic V, Ilic V, et al. Significant impact of behavioral and cognitive impairment on quality of life in patients with myotonic dystrophy type 1. Clinical Neurology & Neurosurgery. 2014;126:76-81.

Rakocevic-Stojanovic V, Peric S, Savic-Pavicevic D, Pesovic J, Mesaros S, Lavrnic D, et al. Brain sonography insight into the midbrain in myotonic dystrophy type 2. Muscle & Nerve. 2016;53(5):700-4.

Ramaswami U, Whybra C, Parini R, Pintos-Morell G, Mehta A, Sunder-Plassmann G, et al. Clinical manifestations of Fabry disease in children: data from the Fabry Outcome Survey. Acta Paediatrica. 2006;95(1):86-92.

Ramdharry GM, Pollard AJ, Marsden JF, Reilly MM. Comparing gait performance of people with Charcot-Marie-Tooth disease who do and do not wear ankle foot orthoses. Physiotherapy Research International. 2012;17(4):191-9.

Ramdharry GM, Thornhill A, Mein G, Reilly MM, Marsden JF. Exploring the experience of fatigue in people with Charcot-Marie-Tooth disease. Neuromuscular Disorders. 2012;22:S208-13.

Rand-Hendriksen S, Sorensen I, Holmstrom H, Andersson S, Finset A. Fatigue, cognitive functioning and psychological distress in Marfan syndrome, a pilot study. Psychology Health & Medicine. 2007;12(3):305-13.

Rao SS, Venuti KD, Dietz HC, 3rd, Sponseller PD. Quantifying Health Status and Function in Marfan Syndrome. Journal of Surgical Orthopaedic Advances. 2016;25(1):34-40.

Rattay TW, Boldt A, Volker M, Wiethoff S, Hengel H, Schule R, et al. Non-motor symptoms are relevant and possibly treatable in hereditary spastic paraplegia type 4 (SPG4). Journal of Neurology. 2020;267(2):369-79.

Reynaud V, Conforto I, Givron P, Clavelou P, Cornut-Chauvinc C, Taithe F, et al. Multidimensional evaluation is necessary to assess hand function in patients with Charcot-Marie-Tooth disease type 1A. Annals of Physical & Rehabilitation Medicine. 2020;25:25.

Salek MS, Ionova T, Johns JR, Oliva EN. Appraisal of patient-reported outcome measures in analogous diseases and recommendations for use in phase II and III clinical trials of pyruvate kinase deficiency. Quality of Life Research. 2019;28(2):399-410.

Samuels N, Elstein D, Lebel E, Zimran A, Oberbaum M. Acupuncture for symptoms of Gaucher disease. Complementary therapies in medicine. 2012;20(3):131-4.

Sansone VA, Ricci C, Montanari M, Apolone G, Rose M, Meola G, et al. Measuring quality of life impairment in skeletal muscle channelopathies. European Journal of Neurology. 2012;19(11):1470-6.

Savas M, Wester VL, Dykgraaf RHM, van den Akker ELT, Roos-Hesselink JW, Dessens AB, et al. Long-term cortisol exposure and associations with height and comorbidities in Turner syndrome. Journal of Clinical Endocrinology & Metabolism. 2019;11:11.

Scheidegger O, Leupold D, Sauter R, Findling O, Rosler KM, Hundsberger T. 36-Months follow-up assessment after cessation and resuming of enzyme replacement therapy in late onset Pompe disease: data from the Swiss Pompe Registry. Journal of Neurology. 2018;265(12):2783-8.

Schillings ML, Kalkman JS, Janssen HM, van Engelen BG, Bleijenberg G, Zwarts MJ. Experienced and physiological fatigue in neuromuscular disorders. Clinical Neurophysiology. 2007;118(2):292-300.

Schipper K, Bakker M, Abma T. Fatigue in facioscapulohumeral muscular dystrophy: a qualitative study of people's experiences. Disability & Rehabilitation. 2017;39(18):1840-6.

Schmitz B, Thorwesten L, Lenders M, Duning T, Stypmann J, Br, et al. Physical Exercise in Patients with Fabry Disease - A Pilot Study. International Journal of Sports Medicine. 2016;37(13):1066-72.

Schoser B, Bilder DA, Dimmock D, Gupta D, James ES, Prasad S. The humanistic burden of Pompe disease: are there still unmet needs? A systematic review. BMC Neurology. 2017;17(1):202.

Schrezenmeier H, Muus P, Socie G, Szer J, Urbano-Ispizua A, Maciejewski JP, et al. Baseline characteristics and disease burden in patients in the International Paroxysmal Nocturnal Hemoglobinuria Registry. Haematologica. 2014;99(5):922-9.

Schubart JR, Schaefer E, Hakim AJ, Francomano CA, Bascom R. Use of Cluster Analysis to Delineate Symptom Profiles in an Ehlers-Danlos Syndrome Patient Population. Journal of Pain & Symptom Management. 2019;58(3):427-36.

Schubert J, Hillmen P, Röth A, Young NS, Elebute MO, Szer J, et al. Eculizumab, a terminal complement inhibitor, improves anaemia in patients with paroxysmal nocturnal haemoglobinuria. British journal of haematology. 2008;142(2):263-72.

Servelhere KR, Faber I, Martinez A, Nickel R, Moro A, Germiniani FMB, et al. Botulinum toxin for hereditary spastic paraplegia: effects on motor and non-motor manifestations. Arquivos de Neuro-Psiquiatria. 2018;76(3):183-8.

Servelhere KR, Faber I, Saute JA, Moscovich M, D'Abreu A, Jardim LB, et al. Non-motor symptoms in patients with hereditary spastic paraplegia caused by SPG4 mutations. European Journal of Neurology. 2016;23(2):408-11.

Simon A, Pompilus F, Querbes W, Wei A, Strzok S, Penz C, et al. Patient Perspective on Acute Intermittent Porphyria with Frequent Attacks: A Disease with Intermittent and Chronic Manifestations. The Patient: Patient-Centered Outcomes Research. 2018;11(5):527-37.

Smith AE, McMullen K, Jensen MP, Carter GT, Molton IR. Symptom burden in persons with myotonic and facioscapulohumeral muscular dystrophy. American Journal of Physical Medicine & Rehabilitation. 2014;93(5):387-95.

Smits BW, Fermont J, Delnooz CC, Kalkman JS, Bleijenberg G, van Engelen BG. Disease impact in chronic progressive external ophthalmoplegia: more than meets the eye. Neuromuscular Disorders. 2011;21(4):272-8.

Statland JM, Bundy BN, Wang Y, Rayan DR, Trivedi JR, Sansone VA, et al. Mexiletine for symptoms and signs of myotonia in nondystrophic myotonia: a randomized controlled trial. Jama. 2012;308(13):1357-65.

Sumpter R, Dorris L, Brannan G, Carachi R. Quality of life and behavioural adjustment in childhood hydrocephalus. Scottish Medical Journal. 2012;57(1):18-25.

Symonds T, Randall JA, Campbell P. Review of patient-reported outcome measures for use in myotonic dystrophy type 1 patients. Muscle & Nerve. 2017;56(1):86-92.

Talaei-Khoei M, Riklin E, Merker VL, Sheridan MR, Jordan JT, Plotkin SR, et al. First use of patient reported outcomes measurement information system (PROMIS) measures in adults with neurofibromatosis. Journal of Neuro-Oncology. 2017;131(2):413-9.

Tarnopolsky M, Katzberg H, Petrof BJ, Sirrs S, Sarnat HB, Myers K, et al. Pompe Disease: Diagnosis and Management. Evidence-Based Guidelines from a Canadian Expert Panel. Canadian Journal of Neurological Sciences. 2016;43(4):472-85.

Ter Haar NM, Jeyaratnam J, Lachmann HJ, Simon A, Brogan PA, Doglio M, et al. The Phenotype and Genotype of Mevalonate Kinase Deficiency: A Series of 114 Cases From the Eurofever Registry. Arthritis & Rheumatology. 2016;68(11):2795-805.

Theodore-Oklota C, Bonner N, Spencer H, Arbuckle R, Chen CY, Skrinar A. Qualitative Research to Explore the Patient Experience of X-Linked Hypophosphatemia and Evaluate the Suitability of the BPI-SF and WOMAC R as Clinical Trial End Points. Value in Health. 2018;21(8):973-83.

Tieleman AA, Jenks KM, Kalkman JS, Borm G, van Engelen BG. High disease impact of myotonic dystrophy type 2 on physical and mental functioning. Journal of Neurology. 2011;258(10):1820-6.

Tieleman AA, Knoop H, van de Logt AE, Bleijenberg G, van Engelen BG, Overeem S. Poor sleep quality and fatigue but no excessive daytime sleepiness in myotonic dystrophy type 2. Journal of Neurology, Neurosurgery & Psychiatry. 2010;81(9):963-7.

Tolboom N, Cats EA, Helders PJ, Pruijs JE, Engelbert RH. Osteogenesis imperfecta in childhood: effects of spondylodesis on functional ability, ambulation and perceived competence. European Spine Journal. 2004;13(2):108-13.

Tosi LL, Oetgen ME, Floor MK, Huber MB, Kennelly AM, McCarter RJ, et al. Initial report of the osteogenesis imperfecta adult natural history initiative. Orphanet journal of rare diseases. 2015;10(1):1-12.

Trip J, de Vries J, Drost G, Ginjaar HB, van Engelen BG, Faber CG. Health status in non-dystrophic myotonias: close relation with pain and fatigue. Journal of Neurology. 2009;256(6):939-47.

Van Brussel M, Takken T, Uiterwaal CS, Pruijs HJ, Van der Net J, Helders PJ, et al. Physical training in children with osteogenesis imperfecta. Journal of Pediatrics. 2008;152(1):111-6, 6.e.

van de Loo KFE, Custers JAE, Koene S, Klein IL, Janssen MCH, Smeitink JAM, et al. Psychological functioning in children suspected for mitochondrial disease: the need for care. Orphanet Journal Of Rare Diseases. 2020;15(1):76.

van den Hoven AT, Bons LR, Dykgraaf RHM, Dessens AB, Pastoor H, de Graaff LCG, et al. A value-based healthcare approach: Health-related quality of life and psychosocial functioning in women with Turner syndrome. Clinical Endocrinology. 2020;31(5):31.

van der Kooi EL, Kalkman JS, Lindeman E, Hendriks JC, van Engelen BG, Bleijenberg G, et al. Effects of training and albuterol on pain and fatigue in facioscapulohumeral muscular dystrophy. Journal of Neurology. 2007;254(7):931-40.

van der Linden MH, Kalkman JS, Hendricks HT, Schillings ML, Zwarts MJ, Bleijenberg G, et al. Ambulatory disabilities and the use of walking aids in patients with hereditary motor and sensory neuropathy type I (HMSN I). Disability & Rehabilitation Assistive Technology. 2007;2(1):35-41.

van der Meijden JC, Gungor D, Kruijshaar ME, Muir AD, Broekgaarden HA, van der Ploeg AT. Ten years of the international Pompe survey: patient reported outcomes as a reliable tool for studying treated and untreated children and adults with non-classic Pompe disease. Journal of Inherited Metabolic Disease. 2015;38(3):495-503.

van der Sluijs BM, Knoop H, Bleijenberg G, van Engelen BG, Voermans NC. The Dutch patients' perspective on oculopharyngeal muscular dystrophy: A questionnaire study on fatigue, pain and impairments. Neuromuscular Disorders. 2016;26(3):221-6.

van der Werf S, Kalkman J, Bleijenberg G, van Engelen B, Schillings M, Zwarts M, et al. The relation between daytime sleepiness, fatigue, and reduced motivation in patients with adult onset myotonic dystrophy. Journal of Neurology, Neurosurgery & Psychiatry. 2003;74(1):138-9.

van Dijk N, Boer MC, Mulder BJ, van Montfrans GA, Wieling W. Is fatigue in Marfan syndrome related to orthostatic intolerance? Clinical Autonomic Research. 2008;18(4):187-93.

van Dorst M, Okkersen K, Kessels RPC, Meijer FJA, Monckton DG, van Engelen BGM, et al. Structural white matter networks in myotonic dystrophy type 1. NeuroImage Clinical. 2019;21:101615.

Van Heugten C, Meuleman S, Hellebrekers D, Kruitwagen-van Reenen E, Visser-Meily J. Participation and the Role of Neuropsychological Functioning in Myotonic Dystrophy Type 1. Journal of neuromuscular diseases. 2018;5(2):205-14.

van Ruitenbeek E, Custers JAE, Verhaak C, Snoeck M, Erasmus CE, Kamsteeg EJ, et al. Functional impairments, fatigue and quality of life in RYR1-related myopathies: A questionnaire study. Neuromuscular Disorders. 2019;29(1):30-8.

Vassallo G, Mughal Z, Robinson L, Weisberg D, Roberts SA, Hupton E, et al. Perceived fatigue in children and young adults with neurofibromatosis type 1. Journal of Paediatrics & Child Health. 2020;9(6):09.

Veenhuizen Y, Cup EHC, Jonker MA, Voet NBM, van Keulen BJ, Maas DM, et al. Self-management program improves participation in patients with neuromuscular disease: A randomized controlled trial. Neurology. 2019;93(18):e1720-e31.

Velvin G, Bathen T, -Hendriksen S, Geirdal AO. Work participation in adults with Marfan syndrome: Demographic characteristics, MFS related health symptoms, chronic pain, and fatigue. American Journal of Medical Genetics Part A. 2015;167(12):3082-90.

Velvin G, Bathen T, -Hendriksen S, Geirdal AO. Satisfaction with life in adults with Marfan syndrome (MFS): associations with health-related consequences of MFS, pain, fatigue, and demographic factors. Quality of Life Research. 2016;25(7):1779-90.

Verderese CL, Graham OC, Holder-McShane CA, Harnett NE, Barton NW. Gaucher's disease: a pilot study of the symptomatic responses to enzyme replacement therapy. The Journal of neuroscience nursing: journal of the American Association of Neuroscience Nurses. 1993;25(5):296-301.

Vergaelen E, Claes S, Kempke S, Swillen A. High prevalence of fatigue in adults with a 22q11.2 deletion syndrome. American Journal of Medical Genetics Part A. 2017;173(4):858-67.

Verhaak C, de Laat P, Koene S, Tibosch M, Rodenburg R, de Groot I, et al. Quality of life, fatigue and mental health in patients with the m.3243A > G mutation and its correlates with genetic characteristics and disease manifestation. Orphanet Journal Of Rare Diseases. 2016;11:25.

Vincent KA, Carr AJ, Walburn J, Scott DL, Rose MR. Construction and validation of a quality of life questionnaire for neuromuscular disease (INQoL). Neurology. 2007;68(13):1051-7.

Voermans NC, Knoop H, Bleijenberg G, van Engelen BG. Fatigue is associated with muscle weakness in Ehlers-Danlos syndrome: an explorative study. Physiotherapy. 2011;97(2):170-4.

Voermans NC, Knoop H, van de Kamp N, Hamel BC, Bleijenberg G, van Engelen BG. Fatigue is a frequent and clinically relevant problem in Ehlers-Danlos Syndrome. Seminars in Arthritis & Rheumatism. 2010;40(3):267-74.

Voet N, Bleijenberg G, Hendriks J, de Groot I, Padberg G, van Engelen B, et al. Both aerobic exercise and cognitive-behavioral therapy reduce chronic fatigue in FSHD: an RCT. Neurology. 2014;83(21):1914-22.

Wang RY, da Silva Franco JF, Lopez-Valdez J, Martins E, Sutton VR, Whitley CB, et al. The long-term safety and efficacy of vestronidase alfa, rhGUS enzyme replacement therapy, in subjects with mucopolysaccharidosis VII. Molecular Genetics & Metabolism. 2020;129(3):219-27.

Warnink-Kavelaars J, Beelen A, Dekker S, Nollet F, Menke LA, Engelbert RHH. Marfan syndrome in childhood: parents' perspectives of the impact on daily functioning of children, parents and family; a qualitative study. BMC Pediatrics. 2019;19(1):262.

Wei Y, Speechley KN, Zou G, Campbell C. Factors Associated With Health-Related Quality of Life in Children With Duchenne Muscular Dystrophy. Journal of Child Neurology. 2016;31(7):879-86.

Werlauff U, Hojberg A, Firla-Holme R, Steffensen BF, Vissing J. Fatigue in patients with spinal muscular atrophy type II and congenital myopathies: evaluation of the fatigue severity scale. Quality of Life Research. 2014;23(5):1479-88.

White CM, van Doorn PA, Garssen MP, Stockley RC. Interventions for fatigue in peripheral neuropathy. Cochrane Database of Systematic Reviews. 2014(12):CD008146.

White M. Patient Input to Inform the Development of Central Nervous System Outcome Measures in Myotonic Dystrophy. Therapeutic Innovation & Regulatory Science. 2020;22:22.

Wietlisbach M, Benden C, Koutsokera A, Jahn K, Soccal PM, Radtke T. Perceptions towards physical activity in adult lung transplant recipients with cystic fibrosis. PLoS ONE [Electronic Resource]. 2020;15(2):e0229296.

Winblad S, Lindberg C. Perceived fatigue in myotonic dystrophy type 1: a case-control study. BMC Neurology. 2019;19(1):45.

Wolter JM, Bowler SD, Nolan PJ, McCormack JG. Home intravenous therapy in cystic fibrosis: A prospective randomized trial examining clinical, quality of life and cost aspects. European Respiratory Journal. 1997;10(4):896-900.

Wood L, Cordts I, Atalaia A, Marini-Bettolo C, Maddison P, Phillips M, et al. The UK Myotonic Dystrophy Patient Registry: facilitating and accelerating clinical research. Journal of Neurology. 2017;264(5):979-88.

Xiong E, Lynch AE, Corben LA, Delatycki MB, Subramony SH, Bushara K, et al. Health related quality of life in Friedreich Ataxia in a large heterogeneous cohort. Journal of the Neurological Sciences. 2020;410:116642.

Youssof S, Romero-Clark C, Warner T, Plowman E. Dysphagia-related quality of life in oculopharyngeal muscular dystrophy: Psychometric properties of the SWAL-QOL instrument. Muscle & Nerve. 2017;56(1):28-35.

Zion YC, Pappadopulos E, Wajnrajch M, berg DE, Rosenbaum H. Rethinking fatigue in Gaucher disease. Molecular Genetics and Metabolism. 2016;111(1):S30-S1.

Zweers H, Smit D, Leij S, Wanten G, Janssen MCH. Individual dietary intervention in adult patients with mitochondrial disease due to the m.3243 A>G mutation. Nutrition. 2020;69:110544.

References included articles – secondary research articles – reviews, in alphabetical order

Jamieson N, Fitzgerald D, Singh-Grewal D, Hanson CS, Craig JC, Tong A. Children's experiences of cystic fibrosis: a systematic review of qualitative studies. Pediatrics. 2014;133(6):e1683-97.

Johnston BC, Miller PA, Agarwal A, Mulla S, Khokhar R, De Oliveira K, et al. Limited responsiveness related to the minimal important difference of patient-reported outcomes in rare diseases. Journal of Clinical Epidemiology. 2016;79:10-21.

Martí-Carvajal AJ, An, V, Cardona AF, Solà I. Eculizumab for treating patients with paroxysmal nocturnal hemoglobinuria. Cochrane Database of Systematic Reviews. 2014(10):CD010340.

Schoser B, Bilder DA, Dimmock D, Gupta D, James ES, Prasad S. The humanistic burden of Pompe disease: are there still unmet needs? A systematic review. BMC Neurology. 2017;17(1):202.

Salek MS, Ionova T, Johns JR, Oliva EN. Appraisal of patient-reported outcome measures in analogous diseases and recommendations for use in phase II and III clinical trials of pyruvate kinase deficiency. Quality of Life Research. 2019;28(2):399-410.

Symonds T, Randall JA, Campbell P. Review of patient-reported outcome measures for use in myotonic dystrophy type 1 patients. Muscle & Nerve. 2017;56(1):86-92.

Tarnopolsky M, Katzberg H, Petrof BJ, Sirrs S, Sarnat HB, Myers K, et al. Pompe Disease: Diagnosis and Management. Evidence-Based Guidelines from a Canadian Expert Panel. Canadian Journal of Neurological Sciences. 2016;43(4):472-85.

Zion YC, Pappadopulos E, Wajnrajch M, berg DE, Rosenbaum H. Rethinking fatigue in Gaucher disease. Molecular Genetics and Metabolism. 2016;111(1):S30-S1.

White CM, van Doorn PA, Garssen MP, Stockley RC. Interventions for fatigue in peripheral neuropathy. Cochrane Database of Systematic Reviews. 2014(12):CD008146.

References included articles on diagnostics – validation/ development of fatigue assessment tools, in alphabetical order

Alemdaroglu-Gurbuz, I., Bulut, N., Bozgeyik, S., Ulug, N., Arslan, S. S., Yilmaz, O., & Karaduman, A. (2019). Reliability and validity of the turkish translation of pedsqlTM multidimensional Fatigue scale in Duchenne Muscular Dystrophy. Neurosciences, 24(4), 302-310.

Bradley, J., Dempster, M., Wallace, E., & Elborn, S. (1999). The adaptations of a quality of life questionnaire for routine use in clinical practice: the Chronic Respiratory Disease Questionnaire in cystic fibrosis. Quality of Life Research, 8(1), 65-71. doi:10.1023/a:1026437214170

Cook, K. F., Bamer, A. M., Amtmann, D., Molton, I. R., & Jensen, M. P. (2012). Six patient-reported outcome measurement information system short form measures have negligible age- or diagnosis-related differential item functioning in individuals with disabilities. Archives of Physical Medicine & Rehabilitation, 93(7), 1289-1291. doi:10.1016/j.apmr.2011.11.022

Gallais, B., Gagnon, C., Forgues, G., Cote, I., & Laberge, L. (2017). Further evidence for the reliability and validity of the Fatigue and Daytime Sleepiness Scale. Journal of the Neurological Sciences, 375, 23-26. doi:10.1016/j.jns.2017.01.032

Hermans, M. C., Merkies, I. S., Laberge, L., Blom, E. W., Tennant, A., & Faber, C. G. (2013). Fatigue and daytime sleepiness scale in myotonic dystrophy type 1. Muscle & Nerve, 47(1), 89-95. doi:10.1002/mus.23478

Koene, S., Wortmann, S. B., de Vries, M. C., Jonckheere, A. I., Morava, E., de Groot, I. J., & Smeitink, J. A. (2013). Developing outcome measures for pediatric mitochondrial disorders: which complaints and limitations are most burdensome to patients and their parents? Mitochondrion, 13(1), 15-24. doi:10.1016/j.mito.2012.11.002

Kuo, A., Todd, J. J., Witherspoon, J. W., Lawal, T. A., Elliott, J., Chrismer, I. C., . . . Meilleur, K. G. (2019). Reliability and Validity of Self-Report Questionnaires as Indicators of Fatigue in RYR1-Related Disorders. Journal of neuromuscular diseases, 6(1), 133-141. doi:10.3233/JND-180335

Laberge, L., Gagnon, C., Jean, S., & Mathieu, J. (2005). Fatigue and daytime sleepiness rating scales in myotonic dystrophy: a study of reliability. Journal of Neurology, Neurosurgery & Psychiatry, 76(10), 1403-1405. doi:10.1136/jnnp.2004.043455

Meilleur, K. G., Jain, M. S., Hynan, L. S., Shieh, C. Y., Kim, E., Waite, M., . . . Bonnemann, C. G. (2015). Results of a two-year pilot study of clinical outcome measures in collagen VI- and laminin alpha2-related congenital muscular dystrophies. Neuromuscular Disorders, 25(1), 43-54. doi:10.1016/j.nmd.2014.09.010

Naik, H., Overbey, J. R., Montgomery, G. H., Winkel, G., Balwani, M., Anderson, K. E., . . . Desnick, R. J. (2020). Evaluating the Patient-Reported Outcomes Measurement Information System scales in acute intermittent porphyria. Genetics in Medicine, 22(3), 590-597. doi:10.1038/s41436-019-0683-y

Parikh, S., Galioto, R., Lapin, B., Haas, R., Hirano, M., Koenig, M. K., . . . Karaa, A. (2019). Fatigue in primary genetic mitochondrial disease: No rest for the weary. Neuromuscular Disorders, 29(11), 895-902. doi:10.1016/j.nmd.2019.09.012

Paulsen, E. K., Friedman, L. S., Myers, L. M., & Lynch, D. R. (2010). Health-related quality of life in children with Friedreich ataxia. Pediatric Neurology, 42(5), 335-337. doi:https://dx.doi.org/10.1016/j.pediatrneurol.2010.01.002

Vincent, K. A., Carr, A. J., Walburn, J., Scott, D. L., & Rose, M. R. (2007). Construction and validation of a quality of life questionnaire for neuromuscular disease (INQoL). *Neurology, 68*(13), 1051-1057. doi:10.1212/01.wnl.0000257819.47628.41

Werlauff, U., Hojberg, A., Firla-Holme, R., Steffensen, B. F., & Vissing, J. (2014). Fatigue in patients with spinal muscular atrophy type II and congenital myopathies: evaluation of the fatigue severity scale. *Quality of Life Research, 23*(5), 1479-1488. doi:10.1007/s11136-013-0565-8

Youssof, S., Romero-Clark, C., Warner, T., & Plowman, E. (2017). Dysphagia-related quality of life in oculopharyngeal muscular dystrophy: Psychometric properties of the SWAL-QOL instrument. *Muscle & Nerve, 56*(1), 28-35. doi:10.1002/mus.25441

References included articles on intervention effects, in alphabetical order

Medical/ drug interventions

Akintoye, S. O., Kelly, M. H., Brillante, B., Cherman, N., Turner, S., Butman, J. A., . . . Collins, M. T. (2006). Pegvisomant for the treatment of gsp-mediated growth hormone excess in patients with McCune-Albright syndrome. Journal of Clinical Endocrinology & Metabolism, 91(8), 2960-2966.

Berntsson, S. G., Gauffin, H., Melberg, A., Holtz, A., & tblom, A. M. (2019). Inherited Ataxia and Intrathecal Baclofen for the Treatment of Spasticity and Painful Spasms. Stereotactic & Functional Neurosurgery, 97(1), 18-23. doi:https://dx.doi.org/10.1159/000497165

Dwyer, T. J., Robbins, L., Kelly, P., Piper, A. J., Bell, S. C., & Bye, P. T. (2015). Non-invasive ventilation used as an adjunct to airway clearance treatments improves lung function during an acute exacerbation of cystic fibrosis: a randomised trial. Journal of Physiotherapy, 61(3), 142-147. doi:10.1016/j.jphys.2015.05.019

Guffon, N., & Fouilhoux, A. (2004). Clinical benefit in Fabry patients given enzyme replacement therapy--a case series. Journal of Inherited Metabolic Disease, 27(2), 221-227. doi:10.1023/B:BOLI.0000028726.11177.8b

Gungor, D., de Vries, J. M., Brusse, E., Kruijshaar, M. E., Hop, W. C., Murawska, M., . . . van der Ploeg, A. T. (2013). Enzyme replacement therapy and fatigue in adults with Pompe disease. Molecular Genetics & Metabolism, 109(2), 174-178. doi:10.1016/j.ymgme.2013.03.016

Haller, C., Song, W., Cimms, T., Chen, C. Y., Whitley, C. B., Wang, R. Y., . . . Harmatz, P. (2019). Individual heat map assessments demonstrate vestronidase alfa treatment response in a highly heterogeneous mucopolysaccharidosis VII study population. Jimd Reports, 49(1), 53-62. doi:10.1002/jmd2.12043

Harmatz, P., Whitley, C. B., Wang, R. Y., Bauer, M., Song, W., Haller, C., & Kakkis, E. (2018). A novel Blind Start study design to investigate vestronidase alfa for mucopolysaccharidosis VII, an ultra-rare genetic disease. Molecular Genetics & Metabolism, 123(4), 488-494. doi:10.1016/j.ymgme.2018.02.006

Hillmen, P., Young, N. S., Schubert, J., Brodsky, R. A., Socié, G., Muus, P., . . . Nakamura, R. (2006). The complement inhibitor eculizumab in paroxysmal nocturnal hemoglobinuria. New England Journal of Medicine, 355(12), 1233-1243. Retrieved from https://www.nejm.org/doi/pdf/10.1056/NEJMoa061648?articleTools=true

Karaa, A., Haas, R., Goldstein, A., Vockley, J., & Cohen, B. H. (2020). A randomized crossover trial of elamipretide in adults with primary mitochondrial myopathy. Journal of Cachexia, Sarcopenia and Muscle, 25, 25. doi:10.1002/jcsm.12559

MacDonald, J. R., Hill, J. D., & Tarnopolsky, M. A. (2002). Modafinil reduces excessive somnolence and enhances mood in patients with myotonic dystrophy. Neurology, 59(12), 1876-1880. doi:10.1212/01.wnl.0000037481.08283.51

Micallef, J., Attarian, S., Dubourg, O., Gonnaud, P.-M., Hogrel, J.-Y., Stojkovic, T., . . . Vacherot, F. (2009). Effect of ascorbic acid in patients with Charcot–Marie–Tooth disease type 1A: a multicentre, randomised, double-blind, placebo-controlled trial. The Lancet Neurology, 8(12), 1103-1110. Retrieved from https://www.thelancet.com/journals/laneur/article/PIIS1474-4422(09)70260-1/fulltext

O'Donoghue, F. J., Borel, J. C., Dauvilliers, Y., Levy, P., Tamisier, R., & Pepin, J. L. (2017). Effects of 1-month withdrawal of ventilatory support in hypercapnic myotonic dystrophy type 1. Respirology, 22(7), 1416-1422. doi:10.1111/resp.13068

Pareyson, D., Reilly, M. M., Schenone, A., Fabrizi, G. M., Cavallaro, T., Manganelli, L., . . . et al. (2011). Ascorbic acid in charcot-marie-tooth disease type 1A (CMTTRIAAL and CMT-TRAUK): a double-blind randomised trial. Lancet Neurology, 10(4), 320-328. Retrieved from https://www.cochranelibrary.com/central/doi/10.1002/central/CN-00785980/fullhttps://www.thelancet.com/pdfs/journals/laneur/PIIS1474-4422(11)70025-4.pdf

Scheidegger, O., Leupold, D., Sauter, R., Findling, O., Rosler, K. M., & Hundsberger, T. (2018). 36-Months follow-up assessment after cessation and resuming of enzyme replacement therapy in late onset Pompe disease: data from the Swiss Pompe Registry. Journal of Neurology, 265(12), 2783-2788. doi:10.1007/s00415-018-9065-7

Schubert, J., Hillmen, P., Röth, A., Young, N. S., Elebute, M. O., Szer, J., . . . Geller, R. (2008). Eculizumab, a terminal complement inhibitor, improves anaemia in patients with paroxysmal nocturnal haemoglobinuria. British Journal of Haematology, 142(2), 263-272. Retrieved from https://onlinelibrary.wiley.com/doi/pdfdirect/10.1111/j.1365-2141.2008.07183.x?download=true

Servelhere, K. R., Faber, I., Martinez, A., Nickel, R., Moro, A., Germiniani, F. M. B., . . . Franca, M. C., Jr. (2018). Botulinum toxin for hereditary spastic paraplegia: effects on motor and non-motor manifestations. Arquivos de Neuro-Psiquiatria, 76(3), 183-188. doi:10.1590/0004-282x20180013

Statland, J. M., Bundy, B. N., Wang, Y., Rayan, D. R., Trivedi, J. R., Sansone, V. A., . . . Consortium for Clinical Investigation of Neurologic, C. (2012). Mexiletine for symptoms and signs of myotonia in nondystrophic myotonia: a randomized controlled trial. Jama, 308(13), 1357-1365. doi:10.1001/jama.2012.12607

Verderese, C. L., Graham, O. C., Holder-McShane, C. A., Harnett, N. E., & Barton, N. W. (1993). Gaucher's disease: a pilot study of the symptomatic responses to enzyme replacement therapy. The Journal of neuroscience nursing: journal of the American Association of Neuroscience Nurses, 25(5), 296-301. Retrieved from https://europepmc.org/article/med/8270810

Wang, R. Y., da Silva Franco, J. F., Lopez-Valdez, J., Martins, E., Sutton, V. R., Whitley, C. B., . . . Harmatz, P. (2020). The long-term safety and efficacy of vestronidase alfa, rhGUS enzyme replacement therapy, in subjects with mucopolysaccharidosis VII. Molecular Genetics & Metabolism, 129(3), 219-227. doi:10.1016/j.ymgme.2020.01.003

Wolter, J. M., Bowler, S. D., Nolan, P. J., & McCormack, J. G. (1997). Home intravenous therapy in cystic fibrosis: A prospective randomized trial examining clinical, quality of life and cost aspects. European Respiratory Journal, 10(4), 896-900. Retrieved from <Go to ISI>://WOS:A1997WW35700022

Rehabilitation interventions

Alemdaroglu, I., Karaduman, A. A., & Yilmaz, O. (2012). Acute effects of different exercises on hemodynamic responses and fatigue in Duchenne muscular dystrophy. Fizyoterapi Rehabilitasyon, 23(1), 10-16.

Andersen, G., Heje, K., Buch, A. E., & Vissing, J. (2017). High-intensity interval training in facioscapulohumeral muscular dystrophy type 1: a randomized clinical trial. J Neurol, 264(6), 1099-1106. doi:10.1007/s00415-017-8497-9

Bankole, L. C., Millet, G. Y., Temesi, J., Bachasson, D., Ravelojaona, M., Wuyam, B., . . . Feasson, L. (2016). Safety and efficacy of a 6-month home-based exercise program in patients with facioscapulohumeral muscular dystrophy: A randomized controlled trial. Medicine, 95(31), e4497. doi:10.1097/MD.0000000000004497

Bates, M. G., Newman, J. H., Jakovljevic, D. G., Hollingsworth, K. G., Alston, C. L., Zalewski, P., . . . Gorman, G. S. (2013). Defining cardiac adaptations and safety of endurance training in patients with m.3243A>G-related mitochondrial disease. International Journal of Cardiology, 168(4), 3599-3608. doi:10.1016/j.ijcard.2013.05.062

Benninghoven, D., Hamann, D., von Kodolitsch, Y., Rybczynski, M., Lechinger, J., Schroeder, F., . . . Hoberg, E. (2017). Inpatient rehabilitation for adult patients with Marfan syndrome: an observational pilot study. Orphanet Journal Of Rare Diseases, 12(1), 127. doi:10.1186/s13023-017-0679-0

Colson, S. S., Benchortane, M., Tanant, V., Faghan, J. P., Fournier-Mehouas, M., Benaim, C., . . . Sacconi, S. (2010). Neuromuscular electrical stimulation training: a safe and effective treatment for facioscapulohumeral muscular dystrophy patients. Archives of Physical Medicine & Rehabilitation, 91(5), 697-702. doi:10.1016/j.apmr.2010.01.019

de Jong, W., van Aalderen, W. M., Kraan, J., Koeter, G. H., & van der Schans, C. P. (2001). Inspiratory muscle training in patients with cystic fibrosis. Respiratory Medicine, 95(1), 31-36. doi:10.1053/rmed.2000.0966

ElMhandi L, Millet GY, Calmels P, Richard A, Oullion R, Gautheron V, et al. Benefits of interval-training on fatigue and functional capacities in Charcot-Marie-Tooth disease. Muscle & Nerve. 2008;37(5):601-10.

Favejee, M. M., van den Berg, L. E., Kruijshaar, M. E., Wens, S. C., Praet, S. F., Pim Pijnappel, W. W., . . . van der Ploeg, A. T. (2015). Exercise training in adults with Pompe disease: the effects on pain, fatigue, and functioning. Archives of Physical Medicine & Rehabilitation, 96(5), 817-822. doi:10.1016/j.apmr.2014.11.020

Okkersen, K., Jimenez-Moreno, C., Wenninger, S., Daidj, F., Glennon, J., Cumming, S., . . . consortium, O. (2018). Cognitive behavioural therapy with optional graded exercise therapy in patients with severe fatigue with myotonic dystrophy type 1: a multicentre, single-blind, randomised trial. Lancet Neurology, 17(8), 671-680. doi:10.1016/S1474-4422(18)30203-5

Schmitz, B., Thorwesten, L., Lenders, M., Duning, T., Stypmann, J., Br, . . . , S. M. (2016). Physical Exercise in Patients with Fabry Disease - A Pilot Study. International Journal of Sports Medicine, 37(13), 1066-1072. doi:10.1055/s-0042-110205

Van Brussel, M., Takken, T., Uiterwaal, C. S., Pruijs, H. J., Van der Net, J., Helders, P. J., & Engelbert, R. H. (2008). Physical training in children with osteogenesis imperfecta. Journal of Pediatrics, 152(1), 111-116, 116.e111. doi:10.1016/j.jpeds.2007.06.029

van der Kooi, E. L., Kalkman, J. S., Lindeman, E., Hendriks, J. C., van Engelen, B. G., Bleijenberg, G., & Padberg, G. W. (2007). Effects of training and albuterol on pain and fatigue in facioscapulohumeral muscular dystrophy. Journal of Neurology, 254(7), 931-940. doi:10.1007/s00415-006-0432-4

Veenhuizen, Y., Cup, E. H. C., Jonker, M. A., Voet, N. B. M., van Keulen, B. J., Maas, D. M., . . . Geurts, A. C. H. (2019). Self-management program improves participation in patients with neuromuscular disease: A randomized controlled trial. Neurology, 93(18), e1720-e1731. doi:https://dx.doi.org/10.1212/WNL.0000000000008393

Voet, N., Bleijenberg, G., Hendriks, J., de Groot, I., Padberg, G., van Engelen, B., & Geurts, A. (2014). Both aerobic exercise and cognitive-behavioral therapy reduce chronic fatigue in FSHD: an RCT. Neurology, 83(21), 1914-1922. doi:10.1212/WNL.0000000000001008

Other interventions

Samuels, N., Elstein, D., Lebel, E., Zimran, A., & Oberbaum, M. (2012). Acupuncture for symptoms of Gaucher disease. *Complementary therapies in medicine, 20*(3), 131-134. Retrieved from https://www.sciencedirect.com/science/article/abs/pii/S096522991100166X?via%3Dihub

Zweers, H., Smit, D., Leij, S., Wanten, G., & Janssen, M. C. H. (2020). Individual dietary intervention in adult patients with mitochondrial disease due to the m.3243 A>G mutation. *Nutrition, 69*, 110544. doi:10.1016/j.nut.2019.06.025

References included articles, on patient’s views and experiences, in alphabetical order

Ballard, L. M., Jenkinson, E., Byrne, C. D., Child, J. C., Davies, J. H., Inskip, H., . . . Fenwick, A. (2018). Lived experience of Silver-Russell syndrome: implications for management during childhood and into adulthood. Archives of Disease in Childhood, 104(1), 76-82.

Belkin, A., Albright, K., Fier, K., Desserich, J., & Swigris, J. J. (2014). "Getting stuck with LAM": patients perspectives on living with lymphangioleiomyomatosis. Health & Quality of Life Outcomes, 12, 79. doi:10.1186/1477-7525-12-79

Boström, K., & Ahlström, G. (2004). Living with a chronic deteriorating disease: the trajectory with muscular dystrophy over ten years. Disability & Rehabilitation, 26(23), 1388-1398. doi:10.1080/0963-8280400000898

Croonen, E. A., Harmsen, M., Van der Burgt, I., Draaisma, J. M., Noordam, K., Essink, M., . . . en, M. W. (2016). Perceived motor problems in daily life: Focus group interviews with people with Noonan syndrome and their relatives. American Journal of Medical Genetics. Part A, 170(9), 2349-2356. doi:10.1002/ajmg.a.37814

Ferizovic, N., Marshall, J., Williams, A. E., Mughal, M. Z., Shaw, N., Mak, C., . . . Upadhyaya, S. (2020). Exploring the Burden of X-Linked Hypophosphataemia: An Opportunistic Qualitative Study of Patient Statements Generated During a Technology Appraisal. Advances in Therapy, 37(2), 770-784. doi:10.1007/s12325-019-01193-0

Gelrud, A., Williams, K. R., Hsieh, A., Gwosdow, A. R., Gilstrap, A., & Brown, A. (2017). The burden of familial chylomicronemia syndrome from the patients' perspective. Expert Review of Cardiovascular Therapy, 15(11), 879-887. doi:10.1080/14779072.2017.1372193

Hayes, R., Grinzaid, K., Duffey, E., & Elsas, L. (1998). The impact of Gaucher disease and its treatment on quality of life. Quality of Life Research, 7(6), 521-534. Retrieved from https://link.springer.com/article/10.1023/A:1008878425167

Johnson, N. E., Quinn, C., Eastwood, E., Tawil, R., & Heatwole, C. R. (2012). Patient-identified disease burden in facioscapulohumeral muscular dystrophy. Muscle & Nerve, 46(6), 951-953. doi:10.1002/mus.23529

Kerstens, H., Satink, T., Nijkrake, M. J., De Swart, B. J. M., Van Lith, B. J. H., Geurts, A. C. H., . . . en, M. W. G. (2019). Stumbling, struggling, and shame due to spasticity: a qualitative study of adult persons with hereditary spastic paraplegia. Disability & Rehabilitation, 1-8. doi:10.1080/09638288.2019.1610084

Lecordier, D., Cartron, E., & Jovic, L. (2017). Understanding people with Steinert's disease to better care for them. Recherche En Soins Infirmiers(131), 13-28. Retrieved from https://www.cairn-int.info/article-E_RSI_131_0013--understanding-people-with-steinert-s.htm#

Lindsay, S., Cagliostro, E., & McAdam, L. (2019). Meaningful occupations of young adults with muscular dystrophy and other neuromuscular disorders. Canadian Journal of Occupational Therapy - Revue Canadienne d Ergotherapie, 86(4), 277-288. doi:10.1177/0008417419832466

Lo, S. H., Lachmann, R., Williams, A., Piglowska, N., & Lloyd, A. J. (2020). Exploring the burden of X-linked hypophosphatemia: a European multi-country qualitative study. Quality of Life Research, 29(7), 1883–1893. doi:10.1007/s11136-020-02465-x

Mazar, I., Stokes, J., Ollis, S., Love, E., Espensen, A., Barth, P. G., . . . Shields, A. L. (2019). Understanding the life experience of Barth syndrome from the perspective of adults: a qualitative one-on-one interview study. Orphanet Journal Of Rare Diseases, 14(1), 243. doi:https://dx.doi.org/10.1186/s13023-019-1200-8

Minis, M. A. H., Satink, T., Kinebanian, A., Engels, J. A., Heerkens, Y. F., van Engelen, B. G. M., & Nijhuis-van der Sanden, M. W. G. (2014). How Persons with a Neuromuscular Disease Perceive Employment Participation: A Qualitative Study. Journal of Occupational Rehabilitation, 24(1), 52-67. Retrieved from <Go to ISI>://WOS:000332020000006

https://link.springer.com/article/10.1007%2Fs10926-013-9447-8

https://link.springer.com/article/10.1007/s10926-013-9447-8

Naik, H., Stoecker, M., erson, S. C., Balwani, M., & Desnick, R. J. (2016). Experiences and concerns of patients with recurrent attacks of acute hepatic porphyria: A qualitative study. Molecular Genetics & Metabolism, 119(3), 278-283. doi:10.1016/j.ymgme.2016.08.006

Nielsen, J., Pelsen, B., & Sorensen, K. (1988). Follow-up of 30 Klinefelter males treated with testosterone. Clinical Genetics, 33(4), 262-269. doi:10.1111/j.1399-0004.1988.tb03447.x

Oladapo, A. O., Ito, D., Hibbard, C., Bean, S. E., Krupnick, R. N., & Ewenstein, B. M. (2019). Patient Experience with Congenital (Hereditary) Thrombotic Thrombocytopenic Purpura: A Conceptual Framework of Symptoms and Impacts. The Patient: Patient-Centered Outcomes Research, 12(5), 503-512. doi:10.1007/s40271-019-00365-y

Ramdharry, G. M., Thornhill, A., Mein, G., Reilly, M. M., & Marsden, J. F. (2012). Exploring the experience of fatigue in people with Charcot-Marie-Tooth disease. Neuromuscular Disorders, 22, S208-213. doi:https://dx.doi.org/10.1016/j.nmd.2012.10.016

Schipper, K., Bakker, M., & Abma, T. (2017). Fatigue in facioscapulohumeral muscular dystrophy: a qualitative study of people's experiences. Disability & Rehabilitation, 39(18), 1840-1846. doi:10.1080/09638288.2016.1212109

Simon, A., Pompilus, F., Querbes, W., Wei, A., Strzok, S., Penz, C., . . . Marquis, P. (2018). Patient Perspective on Acute Intermittent Porphyria with Frequent Attacks: A Disease with Intermittent and Chronic Manifestations. The Patient: Patient-Centered Outcomes Research, 11(5), 527-537. doi:10.1007/s40271-018-0319-3

Theodore-Oklota, C., Bonner, N., Spencer, H., Arbuckle, R., Chen, C. Y., & Skrinar, A. (2018). Qualitative Research to Explore the Patient Experience of X-Linked Hypophosphatemia and Evaluate the Suitability of the BPI-SF and WOMAC R as Clinical Trial End Points. Value in Health, 21(8), 973-983. doi:10.1016/j.jval.2018.01.013

Warnink-Kavelaars, J., Beelen, A., Dekker, S., Nollet, F., Menke, L. A., & Engelbert, R. H. H. (2019). Marfan syndrome in childhood: parents' perspectives of the impact on daily functioning of

children, parents and family; a qualitative study. BMC Pediatrics, 19(1), 262. doi:10.1186/s12887-019-1612-6

White, M. (2020). Patient Input to Inform the Development of Central Nervous System Outcome Measures in Myotonic Dystrophy. Therapeutic Innovation & Regulatory Science, 22, 22. doi:10.1007/s43441-020-00117-3
