# Supplementary material for: Experienced fatigue in people with rare disorders: a scoping review on characteristics of existing research
Source: Orphanet J Rare Dis. 2022 Jan 10;17:14. doi: 10.1186/s13023-021-02169-6 (PMC8751355; doi:10.1186/s13023-021-02169-6)
Supplement: Supplementary file 5 — Additional file 5. Reference list excluded articles.pdf. List of excluded references with reason for exclusion. [file 13023_2021_2169_MOESM5_ESM.pdf]

Additional file 5. Excluded articles, list of references with reason for exclusion

Reference	Reason for exclusion
Abresch RT, Carter GT, Jensen MP, Kilmer DD. Assessment of pain and health-related quality of life in slowly progressive neuromuscular disease. The American journal of hospice & palliative care. 2002;19(1):39-48.	No separate data on experienced fatigue
Acquaye AA, Vera-Bolanos E, Armstrong TS, Bekele BN, Gilbert MR. Symptom profiles in adult patients with ependymoma: Report from the Ependymoma Outcomes (EO) Project. Neuro-Oncology. 2010;12:iv101.	Conference abstract
Afable MG, 2nd, Lyon DE. Severe fatigue: could it be aplastic anemia? Clinical Journal of Oncology Nursing. 2008;12(4):569-73.	Case study
Ali AS, Saeed S, Masood Y, Asghar N, Rashid A, Faizan M, et al. Bisphosphonate therapy improves biochemical, radiological and clinical parameters in children with osteogenesis imperfecta. Pakistan Paediatric Journal. 2010;34(3):148-53.	Article not available
Amtmann D, Bamer AM, Nery-Hurwit MB, Liljenquist KS, Yorkston K. Factors associated with disease self-efficacy in individuals aging with a disability. Psychology Health & Medicine. 2019;24(10):1171-81.	No separate data on experienced fatigue in rare disorder
Andrews JA, Paganoni S, Braastad C, Cudkowicz M, Atassi N. Disease burden in upper motor neuron syndromes: A survey of patient perspectives. Journal of Clinical Neuromuscular Disease. 2014;16(2):104-5.	No separate data on experienced fatigue in rare disorder
Angelini C, Tasca E. Fatigue in muscular dystrophies. Neuromuscular Disorders. 2012;22:S214-20.	No separate data on experienced fatigue
Angelini C, Tasca E. Fatigue in muscular dystrophy and metabolic myopathy. Clinical Neurophysiology. 2016;127:e90.	Conference abstract
Arca M, Hsieh A, Soran H, Rosenblit P, O'Dea L, Stevenson M. The effect of volanesorsen treatment on the burden associated with familial chylomicronemia syndrome: the results of the ReFOCUS study. Expert Review of Cardiovascular Therapy. 2018;16(7):537-46	No separate data on experienced fatigue
Arnold P, Boulat O, Maire R, Kuntzer T. Expanding view of phenotype and oxidative stress in Friedreich's ataxia patients with and without idebenone. Schweizer Archiv fur Neurologie und Psychiatrie. 2006;157(4):169-76.	No separate data on experienced fatigue
Arponen H, Bachour A, Back L, Valta H, Makitie A, Waltimo-Siren J, et al. Is sleep apnea underdiagnosed in adult patients	No separate data on experienced fatigue

with osteogenesis imperfecta? -a single-center cross-sectional study. Orphanet Journal Of Rare Diseases. 2018;13(1):231.	
Azar M, Rice DB, Kwakkenbos L, Carrier ME, Shrier I, Bartlett SJ, et al. Exercise habits and factors associated with exercise in systemic sclerosis: a Scleroderma Patient-centered Intervention Network (SPIN) cohort study. Disability & Rehabilitation. 2018;40(17):1997-2003.	Not included diagnosis
Bahmer T, Watz H, Develaska M, Waschki B, Rabe KF, Magnussen H, et al. Physical Activity and Fatigue in Patients with Sarcoidosis. Respiration. 2018;95(1):18-26.	Not included diagnosis
Balaguer A, González de Dios J. Home versus hospital intravenous antibiotic therapy for cystic fibrosis. Cochrane Database of Systematic Reviews. 2015(12):CD001917.	No separate data on experienced fatigue
Battalio SL, Glette M, Alschuler KN, Jensen MP. Anxiety, depression, and function in individuals with chronic physical conditions: A longitudinal analysis. Rehabilitation Psychology. 2018;63(4):532-41.	No separate data on experienced fatigue in rare disorder
Battalio SL, Silverman AM, Ehde DM, Amtmann D, Edwards KA, Jensen MP. Resilience and Function in Adults With Physical Disabilities: An Observational Study. Archives of Physical Medicine & Rehabilitation. 2017;98(6):1158-64.	No separate data on experienced fatigue in rare disorder
Berger KI, Kanters S, Jansen JP, Stewart A, Sparks S, Haack KA, et al. Forced vital capacity and cross-domain late-onset Pompe disease outcomes: an individual patient-level data meta-analysis. Journal of Neurology. 2019;266(9):2312-21.	No separate data on experienced fatigue
Bowen JM, Sobey GJ, Burrows NP, Colombi M, Lavallee ME, Malfait F, et al. Ehlers-Danlos syndrome, classical type. American Journal of Medical Genetics, Part C: Seminars in Medical Genetics. 2017;175(1):27-39.	No separate data on experienced fatigue
Bozovic I, Peric S, Basta I, Kacar A, Nikolic A, Belanovic B, et al. Quality of life in patients with multifocal motor neuropathy from Serbia. Journal of the Neurological Sciences. 2019;399:151-4.	Not included diagnosis
Brod M, Waldmann LT, Smith A, Karpf D. Assessing the Patient Experience of Hypoparathyroidism Symptoms: Development of the Hypoparathyroidism Patient Experience Scale-Symptom (HPES-Symptom). 2020; 13(2):151-162.	Not included diagnosis
Charmari E, Kino T, Ichijo T, Chrousos GP. Generalized glucocorticoid resistance: clinical aspects, molecular mechanisms, and implications of a rare genetic disorder. Journal of Clinical Endocrinology & Metabolism. 2008;93(5):1563-72.	No separate data on experienced fatigue

Cinar FI, Unver V, Yilmaz S, Cinar M, Yilmaz F, Simsek I, et al. Living with scleroderma: Patients' perspectives, a phenomenological study. Rheumatology International. 2012;32(11):3573-9.	Not included diagnosis
Colson SS, Benchortane M, Tanant V, Faghan JP, Fournier-Mehouas M, Benaim C, et al. Electrical stimulation of shoulder girdle and quadriceps femoris in focioscapulohumeral muscular dystrophy patients. Annals of Physical and Rehabilitation Medicine. 2010;53:e109.	Conference abstract
Cook KF. Fatigue in Spinal Cord Injury (SCI), Muscular Dystrophy (MD), Post-Polio Syndrome (PPS) and Multiple Sclerosis (MS): Age Group Comparisons to US Norms. Quality of Life Research. 2010;19:87-8.	Conference abstract
Cook KF, Molton IR, Jensen MP. Fatigue and aging with a disability. Archives of Physical Medicine & Rehabilitation. 2011;92(7):1126-33.	No separate data on experienced fatigue in rare disorder
Cooper D, Guelcher C, Recht M, Sidonio R, Clark D, Batt K. Anxiety and depression in adults with mild-to-severe hemophilia: Insights from the Hemophilia Experiences, Results and Opportunities (HERO), Pain, Functional Impairment, and Quality of Life (P-FiQ), and Bridging Hemophilia B Experiences, Results and Opportunities into Solutions (B-HERO-S) studies. Haemophilia. 2018;24:82-3.	Conference abstract
da Silva CB, Chevis CF, D'Abreu A, Lopes-Cendes I, Franca MC, Jr. Fatigue is frequent and multifactorial in Friedreich's ataxia. Parkinsonism & Related Disorders. 2013;19(8):766-7.	Letter to editor
Dahlqvist J, Orlen H, Matsson H, Dahl N, Lonnerholm T, Gustavson KH. Multiple epiphyseal dysplasia. Acta Orthopaedica. 2009;80(6):711-5.	No separate data on experienced fatigue
de Barcelos IP, Emmanuele V, Hirano M. Advances in primary mitochondrial myopathies. Current Opinion in Neurology. 2019;32(5):715-21.	No separate data on experienced fatigue in rare disorder
Edwards KA, Alschuler KA, Ehde DM, Battalio SL, Jensen MP. Changes in Resilience Predict Function in Adults With Physical Disabilities: A Longitudinal Study. Archives of Physical Medicine and Rehabilitation. 2017;98(2):329-36.	No separate data on experienced fatigue in rare disorder
Efthymiadou O, Mossman J, Kanavos P. Health related quality of life aspects not captured by EQ-5D-5L: Results from an international survey of patients. Health Policy. 2019;123(2):159-65.	No separate data on experienced fatigue in rare disorder
Ershova MV, Illarioshkin SN, Sukhorukov VS. The use of noben for correction of mitochondrial disorders in Friedrich's	Not included language, Russian

disease. Zhurnal Nevrologii i Psikhiatrii Imeni SS Korsakova. 2007;107(9):32-7.	
Feasson L, Camdessanche JP, M E, hi L, Calmels P, Millet GY. Fatigue and neuromuscular diseases. Annales de Readaptation et de Medecine Physique. 2006;49(6):289-300, 75-284.	Not included language, French
Fjermestad KW. Health complaints and work experiences among adults with neurofibromatosis 1. Occupational Medicine (Oxford). 2019;69(7):504.510.	No separate data on experienced fatigue
Fortuyn HAD, Fronczek R, Smitshoek M, Overeem S, Lappenschaar M, Kalkman J, et al. Severe fatigue in narcolepsy with cataplexy. Journal of Sleep Research. 2012;21(2):163-9.	Not included diagnosis
Franco R. The sleep/wake cycle: Fatigue, sleep, insomnia and their effects on cystic fibrosis health; tackling sleep issues in the adult with CF. Pediatric Pulmonology. 2016;51:136-8.	Conference abstract
Fujino H, Matsumura T, Saito T, Shingaki H, Takahashi MP, Nakayama T, et al. Executive function may affect the gap between subjective and objective evaluation of symptom severity in patients with myotonic dystrophy. Journal of the Neurological Sciences. 2017;381:274-5.	Conference abstract
Gagnon DM, Pergament E and Fine BA. Demographic studies from a National Gaucher Disease Screening Program. Journal of genetic counseling. 1998; 7(5): 385-399	No separate data on experienced fatigue in rare disorder
Gaisl T, Giunta C, Bratton DJ, Sutherl, K, Schlatzer C, et al. Obstructive sleep apnoea and quality of life in Ehlers-Danlos syndrome: a parallel cohort study. Thorax. 2017;72(8):729-35.	No separate data on experienced fatigue in rare disorder
Gelauff JM, Kingma EM, Kalkman JS, Bezemer R, van Engelen BGM, Stone J, et al. Fatigue, not self-rated motor symptom severity, affects quality of life in functional motor disorders. Journal of Neurology. 2018;265(8):1803-9.	No separate data on experienced fatigue in rare disorder
Glorieux FH. Bisphosphonate therapy for severe osteogenesis imperfecta. Journal of Pediatric Endocrinology & Metabolism. 2000;13:989-92.	Conference abstract
Glorieux FH, Bishop NJ, Plotkin H, Chabot G, Lanoue G, Travers R. Cyclic administration of pamidronate in children with severe osteogenesis imperfecta. New England Journal of Medicine. 1998;339(14):947-52.	No separate data on experienced fatigue in rare disorder
Gluud C, Christensen E. Ursodeoxycholic acid for primary biliary cirrhosis. Cochrane Database of Systematic Reviews. 2002(1):CD000551.	Not included diagnosis

Graham CD, Rose MR, Grunfeld EA, Kyle SD, Weinman J. A systematic review of quality of life in adults with muscle disease. Journal of Neurology. 2011;258(9):1581-92.	No separate data on experienced fatigue in rare disorder
Graham CD, Simmons Z, Stuart SR, Rose MR. The potential of psychological interventions to improve quality of life and mood in muscle disorders. Muscle & Nerve. 2015;52(1):131-6.	No separate data on experienced fatigue in rare disorder
Gruet M. Fatigue in Chronic Respiratory Diseases: Theoretical Framework and Implications For Real-Life Performance and Rehabilitation. Frontiers in Physiology. 2018;9:1285.	No separate data on experienced fatigue in rare disorder
Guilleminault C, Primeau M, Chiu HY, Yuen KM, Leger D, Metlaine A. Sleep-disordered breathing in Ehlers-Danlos syndrome: a genetic model of OSA. Chest. 2013;144(5):1503-11.	No separate data on experienced fatigue in rare disorder
Gutierrez-Rivas E, Illa I, Pascual-Pascual SI, Perez-Lopez J, Vilchez-Padilla JJ, Bautista-Lorite J, et al. Guidelines for monitoring late-onset Pompe disease.Sociedad Espanola de Medicina Interna (SEMI), Sociedad Espanola de Neurologia (SEN) y Sociedad Espanola de Neumologia y CirugiaToracica (SEPAR). Revista de Neurologia. 2015;60(7):321-8.	Not included language, Spanish
Haller C, Song W, Cimms T, Chen CY, Whitley CB, Wang RY, et al. Individual heat map assessments demonstrate ert treatment response in highly heterogeneous mps vii study population. Journal of Inborn Errors of Metabolism and Screening. 2017;5:268-9.	Conference abstract
Hamonet C, Mazaltarine G, Deparcy D. Ehlers-Danlos (EDS), a syndrome that is often overlooked and associated with: Fatigue, pain and proprioception problems. Specific support from rehabilitation medicine. Lettre de Medecine Physique et de Readaptation. 2011;27(4):196-202.	Not included language, French
Hamonet C, Vienne M, Leroux C, Letinaud MP, Paumier J, Dehecq B, et al. Respiratory manifestations in Ehlers-Danlos syndrome (EDS). New treatments options. Journal de Readaptation Medicale. 2016;36(1):56-61.	Not included language, French
Hannan LM, Dominelli GS, Chen YW, Darlene Reid W, Road J. Systematic review of non-invasive positive pressure ventilation for chronic respiratory failure. Respiratory Medicine. 2014;108(2):229-43.	No separate data on experienced fatigue in rare disorder
Harms L, Sieb JP, Williams AE, Graham R, Shlaen R, Claus V, et al. Long-term disease history, clinical symptoms, health status, and healthcare utilization in patients suffering from Lambert Eaton myasthenic syndrome: Results of a patient interview survey in Germany. Journal of Medical Economics. 2012;15(3):521-30.	No separate data on experienced fatigue in rare disorder

Hershenfeld SA, Wasim S, McNiven V, Parikh M, Majewski P, Faghfoury H, et al. Psychiatric disorders in Ehlers-Danlos syndrome are frequent, diverse and strongly associated with pain. Rheumatology International. 2016;36(3):341-8.	No separate data on experienced fatigue in rare disorder
Hilberink SR, van der Slot WMA, Klem M. Health and participation problems in older adults with long-term disability. Disability and Health Journal. 2017;10(2):361-6.	No separate data on experienced fatigue in rare disorder
Hollingsworth KG, Newton JL, Taylor R, McDonald C, Palmer JM, Blamire AM, et al. Pilot Study of Peripheral Muscle Function in Primary Biliary Cirrhosis: Potential Implications for Fatigue Pathogenesis. Clinical Gastroenterology and Hepatology. 2008;6(9):1041-8.	Not included diagnosis
Ireland PJ, Pacey V, Zankl A, Edwards P, Johnston LM, Savarirayan R. Optimal management of complications associated with achondroplasia. Application of Clinical Genetics. 2014;7:117-25.	No separate data on experienced fatigue
Iudici M, Vettori S, Russo B, Giacco V, Capocotta D, Valentini G. Outcome of a glucocorticoid discontinuation regimen in patients with inactive systemic sclerosis. Clinical Rheumatology. 2016;35(8):1985-91.	Not included diagnosis
Johansen H. Når kreftene ikke strekker til. Ergoterapeuten (Oslo). 2002;45(4):18-22.	Nor peer reviewed
Johnson CD, Arbuckle R, Bonner N, Connett G, Dominguez-Munoz E, Levy P, et al. Qualitative Assessment of the Symptoms and Impact of Pancreatic Exocrine Insufficiency (PEI) to Inform the Development of a Patient-Reported Outcome (PRO) Instrument. The Patient: Patient-Centered Outcomes Research. 2017;10(5):615-28.	No separate data on experienced fatigue in rare disorder
Kacar A, Bjelica B, Bozovic I, Peric S, Nikolic A, Cobeljic M, et al. Neuromuscular disease-specific questionnaire to assess quality of life in patients with chronic inflammatory demyelinating polyradiculoneuropathy. Journal of the Peripheral Nervous System. 2018;23(1):11-6.	Not included diagnosis
Khanna D, Denton CP, Jahreis A, van Laar JM, Frech TM, Anderson ME, et al. Safety and efficacy of subcutaneous tocilizumab in adults with systemic sclerosis (faSScinate): a phase 2, randomised, controlled trial. Lancet. 2016;387(10038):2630-40.	Not included diagnosis
Kim J, Chung H, Amtmann D, Salem R, Park R, Askew RL. Symptoms and quality of life indicators among children with chronic medical conditions. Disability and Health Journal. 2014;7(1):96-104.	No separate data on experienced fatigue in rare disorder

Kluger N, Jokinen M, Krohn K, Ranki A. What is the burden of living with autoimmune polyendocrinopathy candidiasis ectodermal dystrophy (APECED) in 2012? A health-related quality-of-life assessment in Finnish patients. Clinical Endocrinology. 2013;79(1):134-41.	No separate data on experienced fatigue in rare disorder
Kuo A, Kuo A, Bowlus CL. Management of symptom complexes in primary biliary cholangitis. Current Opinion in Gastroenterology. 2016;32(3):204-9.	Book chapter
Kuys SS, Hall K, Peasey M, Wood M, Cobb R, Bell SC. Gaming console exercise and cycle or treadmill exercise provide similar cardiovascular demand in adults with cystic fibrosis: a randomised cross-over trial. Journal of Physiotherapy. 2011;57(1):35-40.	No separate data on experienced fatigue
Laffan M, Stephensen D, Camp C, Carroll L, Collins P, Eleston D, et al. The top 10 research priorities in bleeding disorders: A james lind alliance priority setting partnership. Haemophilia. 2019;25:73.	No separate data on experienced fatigue
Lai JS, Nowinski C, Victorson D, Bode R, Podrabsky T, McKinney N, et al. Quality-of-life measures in children with neurological conditions: pediatric Neuro-QOL. Neurorehabilitation & Neural Repair. 2012;26(1):36-47.	No separate data on experienced fatigue in rare disorder
Lee AL, Hill CJ, Cecins N, Jenkins S, McDonald CF, Burge AT, et al. The short and long term effects of exercise training in non-cystic fibrosis bronchiectasis--a randomised controlled trial. Respiratory Research. 2014;15:44.	Not included diagnosis
Lopez Rubio M, Morado M, Gaya A, Alonso Rosa D, Ojeda E, Munoz JA, et al. Paroxysmal nocturnal hemoglobinuria therapy with eculizumab: Spanish experience. Medicina Clinica. 2011;137(1):8-13.	Not included language, Spanish
Maeland S, Assmus J, Berglund B. Subjective health complaints in individuals with Ehlers-Danlos syndrome: a questionnaire study. International Journal of Nursing Studies. 2011;48(6):720-4.	No separate data on experienced fatigue in rare EDS types
Magerl M, Rae W, Aygoren-Pursun E, Bygum A, Panovska VG, Steiner UC, et al. BCX7353 improves health-related quality of life in hereditary angioedema with C1-inhibitor deficiency (C1-INH-HAE): Findings from the APeX-1 study. Allergy: European Journal of Allergy and Clinical Immunology. 2018;73:724.	Conference abstract
Maggi G, Bragadin MM, Padua L, Fiorina E, Bellone E, Gr, et al. Outcome Measures and Rehabilitation Treatment in Patients Affected by Charcot-Marie-Tooth Neuropathy. American	No separate data on experienced fatigue

Journal of Physical Medicine & Rehabilitation. 2011;90(8):628-37.	
Mancuso M, Angelini C, Bertini E, Carelli V, Comi GP, Minetti C, et al. Fatigue and exercise intolerance in mitochondrial diseases. Literature revision and experience of the Italian Network of mitochondrial diseases. Neuromuscular Disorders. 2012;22:S226-9.	No separate data on experienced fatigue
Mathieu F, Begaux F, Suetens C, De Maertelaer V, Hinsenkamp M. Anthropometry and clinical features of Kashin-Beck disease in central Tibet. International Orthopaedics. 2001;25(3):138-41.	No separate data on experienced fatigue in rare disorder
Mazaltarine G, Hamonet C. Pain clinic: Early experience with Ehlers-Danlos patients in a physical rehabilitation center. Journal de Readaptation Medicale. 2008;28(1):33-9.	Not included language, French
Mesa RA, Su Y, Woolfson A, Prchal JT, Turnbull K, Jabbour E, et al. Development of a symptom assessment in patients with myelofibrosis: Qualitative study findings. Health and Quality of Life Outcomes Vol 17 2019, ArtID 61. 2019;17(1):61.	Not included diagnosis
Montali L, Frigerio A, Riva P, Invernizzi P. 'It's as if PBC didn't exist': the illness experience of women affected by primary biliary cirrhosis. Psychology & Health. 2011;26(11):1429-45.	Not included diagnosis
Mosquera RA, Koenig MK, Adejumo RB, Chevallier J, Hashmi SS, Mitchell SE, et al. Sleep disordered breathing in children with mitochondrial disease. Pulmonary Medicine. 2014; 467576	No separate data on experienced fatigue
Newton J, Poyner E, Jones DE. Serum anti-mitochondrial antibodies and fatigue in primary biliary cirrhosis. Liver International. 2009;29(8):1285-6.	Not included diagnosis
Newton, J. L., et al. Fatigue in adult patients with primary immune thrombocytopenia. European Journal of Haematology. 2011:86(5):420-429	Not included diagnosis
Niedeggen C, Singer S, Groth M, Petermann-Meyer A, Roth A, Schrezenmeier H, et al. Design and development of a disease-specific quality of life tool for patients with aplastic anaemia and/or paroxysmal nocturnal haemoglobinuria (QLQ-AA/PNH)-a report on phase III. Annals of Hematology. 2019;98(7):1547-59.	No separate data on experienced fatigue in rare disorder
Niederau C., et al.. Glucocerebrosidase for treatment of Gaucher's disease: first German long-term results. J Hepatol. 1994; 21(4):610-617.	No separate data on experienced fatigue

Palamar D, Güler H, Hancı M, Sucuoğlu H, Sanus GZ, Tüzün Ş. Posturographic examination of body balance in patients with Chiari type I malformation and correlation with the presence of syringomyelia and degree of cerebellar ectopia. Turkish Journal of Physical Medicine & Rehabilitation (2587-0823). 2019;65(1):74-9.	Not included diagnosis
Palomo-Toucedo IC, Vazquez-Bautista C, Munuera-Martinez PV, Dominguez-Maldonado G, Castillo-Lopez JM, Reina-Bueno M. Podiatry alterations in Ehlers-Danlos syndrome. Medicina Clinica. 2020;154(3):94-7.	No separate data on experienced fatigue in rare EDS types
Patel S, Cole AD, Nolan CM, Barker RE, Jones SE, Kon S, et al. Pulmonary rehabilitation in bronchiectasis: a propensity-matched study. European Respiratory Journal. 2019;53(1).	Not included diagnosis
Piepers S, van den Berg LH, Brugman F, Scheffer H, Ruiterkamp-Versteeg M, van Engelen BG, et al. A natural history study of late onset spinal muscular atrophy types 3b and 4. Journal of Neurology. 2008;255(9):1400-4.	No separate data on experienced fatigue
Pinquart M. Psychological Health of Children with Chronic Physical Illness and their Parents - Results from Meta-Analyses. Praxis der Kinderpsychologie und Kinderpsychiatrie. 2017;66(9):656-71.	No separate data on experienced fatigue in rare disorder
Plewa J, Surampalli A, Wencel M, Milad M, Donkervoort S, Caiozzo VJ, et al. A cross-sectional analysis of clinical evaluation in 35 individuals with mutations of the valosin-containing protein gene. Neuromuscular Disorders. 2018;28(9):778-86.	No separate data on experienced fatigue
Plotkin H, Glorieux FH. Medical treatment of osteogenesis imperfecta. Drug Development Research. 2000;49(3):141-5.	No separate data on experienced fatigue
Ponte C, Gray D, Barrett J, Turner A, Teare H, Hogg J, et al. United Kingdom research registry for rare bone, joint and blood vessel diseases (Rudy): Analysis of the first 133 patients. Annals of the Rheumatic Diseases. 2015;74:318.	Conference abstract
Radtke T, Nevitt SJ, Hebestreit H, Kriemler S. Physical exercise training for cystic fibrosis. Cochrane Database of Systematic Reviews. 2017;2017(11):CD002768.	No separate data on experienced fatigue in rare disorder
Raphael JC, Dazord A, Jaillard P, Andronikof-Sanglade A, Benony H, Kovess V, et al. Assessment of quality of life for home ventilated patients with Duchenne muscular dystrophy. Revue Neurologique. 2002;158(4):453-60.	Not included language,<br><br>French
Rasmussen M, Scheie D, Breivik N, Mork M, Lindal S. Clinical and muscle biopsy findings in Norwegian paediatric patients	No separate data on experienced fatigue

with limb girdle muscular dystrophy 2I. Acta Paediatrica. 2014;103(5):553-8.	
Rector Jr WG, Fortuin NJ, Conley CL. Non-hematologic effects of chronic iron deficiency. A study of patients with polycythemia vera treated solely with venesections. Medicine. 1982;61(6):382-9.	Not included diagnosis
Reina-Bueno M, Vazquez-Bautista C, Palomo-Toucedo IC, Dominguez-Maldonado G, Castillo-Lopez JM, Munuera-Martinez PV. Custom-Made Foot Orthoses Reduce Pain and Fatigue in Patients with Ehlers-Danlos Syndrome. A Pilot Study. International Journal of Environmental Research & Public Health [Electronic Resource]. 2020;17(4):20.	No separate data on experienced fatigue in rare EDS types
Richardson D, Thompson AJ. Management of spasticity in hereditary spastic paraplegia. Physiotherapy Research International. 1999;4(1):68-76.	Case study
Roebroeck ME, Jahnsen R, Carona C, Kent RM, Chamberlain MA. Adult outcomes and lifespan issues for people with childhood-onset physical disability. Developmental Medicine & Child Neurology. 2009;51(8):670-8.	No separate data on experienced fatigue in rare disorder
Rofail D, Maguire L, Kissner M, Colligs A, Abetz-Webb L. A review of the social, psychological, and economic burdens experienced by people with spina bifida and their caregivers. Neurology & Therapy. 2013;2(1):1-12.	No separate data on experienced fatigue
Rowe PC, Barron DF, Calkins H, Maumenee IH, Tong PY, Geraghty MT. Orthostatic intolerance and chronic fatigue syndrome associated with Ehlers-Danlos syndrome. Journal of Pediatrics. 1999;135(4):494-9.	No separate data on experienced fatigue in rare EDS types
Sacri AS, Chambaraud T, Ranchin B, Florkin B, See H, Decramer S, et al. Clinical characteristics and outcomes of childhood-onset ANCA-associated vasculitis: a French nationwide study. Nephrology Dialysis Transplantation. 2015;30:i104-12.	Not included diagnosis
Salehpour S, Tavakkoli S. Cyclic pamidronate therapy in children with osteogenesis imperfecta. Journal of Pediatric Endocrinology & Metabolism. 2010;23(1):73-80.	No separate data on experienced fatigue
Salem R, Bamer AM, Alschuler KN, Johnson KL, Amtmann D. Obesity and symptoms and quality of life indicators of individuals with disabilities. Disability & Health Journal. 2014;7(1):124-30.	No separate data on experienced fatigue in rare disorder
Salonini E, Gambazza S, Meneghelli I, Tridello G, Sanguanini M, Cazzarolli C, et al. Active Video Game Playing in Children and Adolescents With Cystic Fibrosis: Exercise or Just Fun? Respiratory Care. 2015;60(8):1172-9.	No separate data on experienced fatigue

Sawicki GS, Sellers DE, Robinson WM. Self-reported physical and psychological symptom burden in adults with cystic fibrosis. Journal of Pain & Symptom Management. 2008;35(4):372-80.	No separate data on experienced fatigue
Scheinberg P, Shore E, Grp PTS. A pilot study of the safety and efficacy of tobramycin solution for inhalation in patients with severe bronchiectasis. Chest. 2005;127(4):1420-6.	Not included diagnosis
Semplicini C, Angelini C. Clinical scales for the evaluation of neuromuscular patients. In: Angelini C, editor. Muscular Dystrophy: Causes and Management: Nova Science publishers, Inc.; 2013. p. 55-66.	Book chapter
Shapiro S, Stephenson D, Camp C, Carroll L, Collins P, Elston D, et al. The top 10 research priorities in bleeding disorders: A james lind alliance priority setting partnership. British Journal of Haematology. 2019;185(4):131.	No separate data on experienced fatigue
Shepard P, Lam EM, St Louis EK, Dominik J. Sleep disturbances in myotonic dystrophy type 2. European Neurology. 2012;68(6):377-80.	No separate data on experienced fatigue
Shoffner J, Hyams LC, Langley GN. Mitochondrial Disease in Adult and Pediatric Patients with Fatigue and Myalgias. Neurology. 2009;72(11):A469-A.	Conference abstract
Sitenga J, Aird G, Ahmed A, Silberstein PT. Impact of siltuximab on patient-related outcomes in multicentric Castleman's disease. Patient Related Outcome Measures. 2018;9:35-41.	Not included diagnosis
Sleiman I, Rozzini R, Trabucchi M. Rare diseases in elderly persons. Journal of the American Geriatrics Society. 2008;56(12):2365-6.	Letter to editor
Spinou A, Fragkos KC, Lee KK, Elston C, Siegert RJ, Loebinger MR, et al. The validity of health-related quality of life questionnaires in bronchiectasis: a systematic review and meta-analysis. Thorax. 2016;71(8):683-94.	Not included diagnosis
Stirnemann J., Rose C, Serratrice C, Dalbies F. et al.. Impact of imiglucerase supply constraint on the therapeutic management and course of disease in French patients with Gaucher disease type 1. Orphanet J Rare Dis. 2015:10(1):1-12	No separate data on experienced fatigue
Sulli A, Talarico R, Scire CA, Avcin T, Castori M, Ferraris A, et al. Ehlers-Danlos syndromes: state of the art on clinical practice guidelines. Rmd Open. 2018;4(Suppl 1):e000790.	No separate data on experienced fatigue in rare EDS types
Swillen A, Devriendt K, Legius E, Eyskens B, Dumoulin M, Gewillig M, et al. Intelligence and psychosocial adjustment in	No separate data on experienced fatigue

velo-cardio-facial syndrome: A study of 37 children and adolescents with VCFS. Genetic Counseling. 1997;8(3):260-1.	
Taivassalo T, De Stefano N, Argov Z, Matthews PM, Chen J, Genge A, et al. Effects of aerobic training in patients with mitochondrial myopathies. Neurology. 1998;50(4):1055-60.	No separate data on experienced fatigue
Tajima F, Nakamura T, Nishimura Y, Arakawa H, Kawasaki T, Ogawa T, et al. Rehabilitation of Charcot-Marie-Tooth Disease. Brain & Nerve / Shinkei Kenkyu no Shinpo. 2016;68(1):59-68.	Not included language, Japanese
Takahashi MP, Yamamoto R, Kubota T, Matsuura T, Ishigaki K, Sunada Y, et al. Study of care practices for patients with myotonic dystrophy in Japan-Nationwide patient survey. Rinsho Shinkeigaku - Clinical Neurology. 2020;60(2):130-6.	Not included language, Japanese
Tessier R, Pilewski JM, Tycon L, Richless C, Jeong K, Yabes J, et al. Discordance between pulmonary function and symptom burden in patients with cystic fibrosis. Pediatric Pulmonology. 2017;52:502.	Conference abstract
Teule DA, Elsinga A, Ruiter N, Meijer JWG. Evaluation of a group education programme for patients experiencing severe fatigue due to neuromuscular disorders or multiple sclerosis. Nederlands Tijdschrift Voor Fysiotherapie. 2009;119(5):148-52.	Not included language, Deutch
Thompson CS, Harrison S, Ashley J, Day K, Smith DL. Flutter valve or conventional physiotherapy in productive non-cystic bronchiectasis? - A randomised crossover study. Thorax. 2000;55:A71.	Conference abstract
Uhlenbusch N, Lowe B, Depping MK. Perceived burden in dealing with different rare diseases: A qualitative focus group study. BMJ Open. 2019; 9 (12):e033353.	No separate data on experienced fatigue
van Capelle CI, van der Meijden JC, van den Hout JM, Jaeken J, Baethmann M, Voit T, et al. Childhood Pompe disease: clinical spectrum and genotype in 31 patients. Orphanet Journal Of Rare Diseases. 2016;11(1):65.	No separate data on experienced fatigue
van Rhee F, Rothman M, Ho KF, Fleming S, Wong RS, Fossa A, et al. Patient-reported Outcomes for Multicentric Castleman's Disease in a Randomized, Placebo-controlled Study of Siltuximab. The Patient: Patient-Centered Outcomes Research. 2015;8(2):207-16.	Not included diagnosis
Van Rijswijk HNAJ, Vorselaars ADM, Ruven HJT, Keijsers RGM, Zanen P, Korenromp IHE, et al. Changes in disease activity, lung function and quality of life in patients with refractory sarcoidosis after anti-TNF treatment. Expert Opinion on Orphan Drugs. 2013;1(6):437-43.	Not included diagnosis

Vancheri C, Albera C, Harari S, Pesci A, Poletti V, Rottoli P, et al. Late breaking abstract-IPF symptoms' course during a 3-month observation: FIBRONET observational study's preliminary results. European Respiratory Journal Conference: European Respiratory Society International Congress, ERS. 2017;50(61).	Conference abstract
Voermans NC, Knoop H, Bleijenberg G, van Engelen BG. Pain in ehlers-danlos syndrome is common, severe, and associated with functional impairment. Journal of Pain & Symptom Management. 2010;40(3):370-8.	No separate data on experienced fatigue in rare EDS types
Wahl AK, Rustoen T, Gjengedal E, Homme J, Hanestad BR. Cystic fibrosis as seen from the patients' perspective. Tidsskrift for Den Norske Laegeforening. 2003;123(18):2580-2.	No separate data on experienced fatigue
Willgoss TG, Humphrey L, Blankenburg M. "I don't know how it happened or when everything changed. It's like i blinked and all of a sudden, I Didn't recognise my own body ": Using qualitative insights to develop a conceptual model to understand the lived experience of patients with systemic sclerosis. Value in Health. 2014;17:A535.	Conference abstract
Wilson CB, Jones PW, Oleary CJ, Cole PJ, Wilson R. Validation of the St. George's Respiratory Questionnaire in bronchiectasis. American Journal of Respiratory and Critical Care Medicine. 1997;156(2):536-41.	Not included diagnosis
Winkel LP, Van den Hout JM, Kamphoven JH, Disseldorp JA, Remmerswaal M, Arts WF, et al. Enzyme replacement therapy in late-onset Pompe's disease: a three-year follow-up. Annals of Neurology. 2004;55(4):495-502.	Case study
Witkop M, Lambing A, Divine G, Kachalsky E, Rushlow D, Dinnen J. A national study of pain in the bleeding disorders community: a description of haemophilia pain. Haemiophilia. 2012;18(3):e115-119.	No separate data on experienced fatigue
Witkop M, Lambing A, Divine G, Kachalsky E, Rushlow D, Dinnen J. A national study of pain in the bleeding disorders community: a description of haemophilia pain. Haemiophilia. 2012;18(3):e115-119.	No separate data on experienced fatigue
Wokke JHJ. Fatigue is part of the burden of neuromuscular diseases 2. Journal of Neurology. 2007;254(7):948-9.	Letter to editor
Wyatt K, Henley W, Anderson L, Anderson R, Nikolaou V, Stein K, et al. The effectiveness and cost-effectiveness of enzyme and substrate replacement therapies: a longitudinal cohort study of people with lysosomal storage disorders. Health Technology Assessment (Winchester, England). 2012;16(39):1-543.	Report

Yildiz S, Inal-Ince D, Calik-Kutukcu E, Vardar-Yagli N, Saglam M, Arikan H, et al. Clinical Determinants of Incremental Shuttle Walk Test in Adults with Bronchiectasis. Lung. 2018;196(3):343-9.	Not included diagnosis
Yurieva EA, Vozdvizhenskaya ES, Kushnareva MV, Semyachkina AN, Kharabadze MN, Sukhorukov VS. Hypoxic syndrome in hereditary diseases of connective tissue. Rossiyskiy Vestnik Perinatologii i Pediatrii. 2019;64(4):60-4.	Not included language, Russian
Zhao XY, Wang WW, Ou XJ, Wang TL, Jia JD. Clinical and pathological features of 27 cases of primary sclerosing cholangitis. Chung Hua Kan Tsang Ping Tsa Chih. 2010;18(9):685-8.	Not included language, Chinese
